# Redesigning donor–acceptor Stenhouse adduct photoswitches through a joint experimental and computational study†

**DOI:** 10.1039/d0sc06575g

**Published:** 2021-01-04

**Authors:** Romain Berraud-Pache, Eduardo Santamaría-Aranda, Bernardo de Souza, Giovanni Bistoni, Frank Neese, Diego Sampedro, Róbert Izsák

**Affiliations:** Max-Planck-Institut für Kohlenforschung Kaiser-Wilhelm Platz 1 45470 Mülheim an der Ruhr Germany robert.izsak@kofo.mpg.de; Sorbonne Université, Laboratoire d’Archéologie Moléculaire et Structurale, CNRS UMR 8220, UPMC – Tour 23, 3ème étage Couloir 23-33, BP 225, 4 place Jussieu 75005 Paris France; Departamento de Química, Centro de Investigación en Síntesis Química (CISQ), Universidad de La Rioja Madre de Dios 53 E-26006 Logroño Spain diego.sampedro@unirioja.es; FAccTs GmbH Rolandstrasse 67 50677 Köln Germany

## Abstract

Many studies have recently explored a new class of reversible photoswitching compounds named Donor–Acceptor Stenhouse Adducts (DASAs). Upon light irradiation, these systems evolve from a coloured open-chain to a colourless closed-ring form, while the thermal back-reaction occurs at room temperature. In order to fulfill the requirements for different applications, new molecules with specific properties need to be designed. For instance, shifting the activation wavelength towards the red part of the visible spectrum is of relevance to biological applications. By using accurate computational calculations, we have designed new DASAs and predicted some of their photophysical properties. Starting from well-studied donor and acceptor parts, we have shown that small chemical modifications can lead to substantial changes in both photophysical and photoswitching properties of the resulting DASAs. Furthermore, we have also analysed how these substitutions impact the electronic structure of the systems. Finally, some pertinent candidates have been successfully synthesized and their photoswitching properties have been characterized experimentally.

## Abstract 2

New photoswitch Donor–Acceptor Stenhouse Adducts (DASAs) have been synthesized thanks to accurate computational chemistry predictions. They possess good properties, notably red light activation.

## Graphical abstract

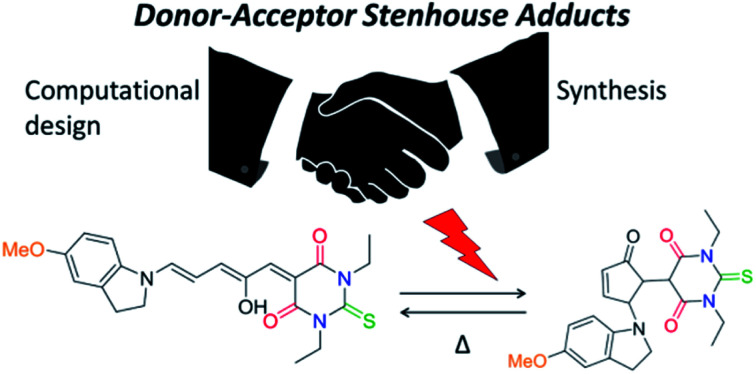

## Introduction

Many molecular systems rely on the interaction with light as an external stimulus to undergo a chemical transformation. Among them, molecular photoswitches are defined by their ability to alternate reversibly between two states upon irradiation. The photoswitching process leads to a complete reorganization of the geometric structure and largely impacts both physical and chemical properties. Nowadays, such molecules are used in a large variety of fields, from material science and supramolecular chemistry^1^ to medical applications including photopharmacology.^2–4^

In order to assess the range of applicability of a photoswitch, several properties have to be evaluated: the absorption wavelength; the quantum yield of photoswitching; the solubility in diverse solvents and the relative stability of the two states. In general, photoswitches can be sorted into two main groups. In the first one, a *Z*–*E* photoisomerization of carbon–carbon, carbon–nitrogen or nitrogen–nitrogen double bonds takes place, as in azobenzenes,^5^ stilbenes^6^ or hemithioindigo photoswitches.^7^ In the second one, the absorbed light is responsible for a cyclization/ring-opening reaction as in diarylethenes^8^ or spiropyrans.^9^

Recently, the so-called Donor–Acceptor Stenhouse Adducts (DASAs) have been reported as an exciting new class of photoswitches.^10,11^ In this case, the use of visible light induces the switching from a strongly colored linear form to a cyclic colorless product. In contrast, the reverse back-reaction is achieved thermally, usually at room temperature. Besides exhibiting negative photochromism,^12^ the photoswitching is also accompanied by large changes in the molecular structure and dipolar moment.

**Fig. 1:**
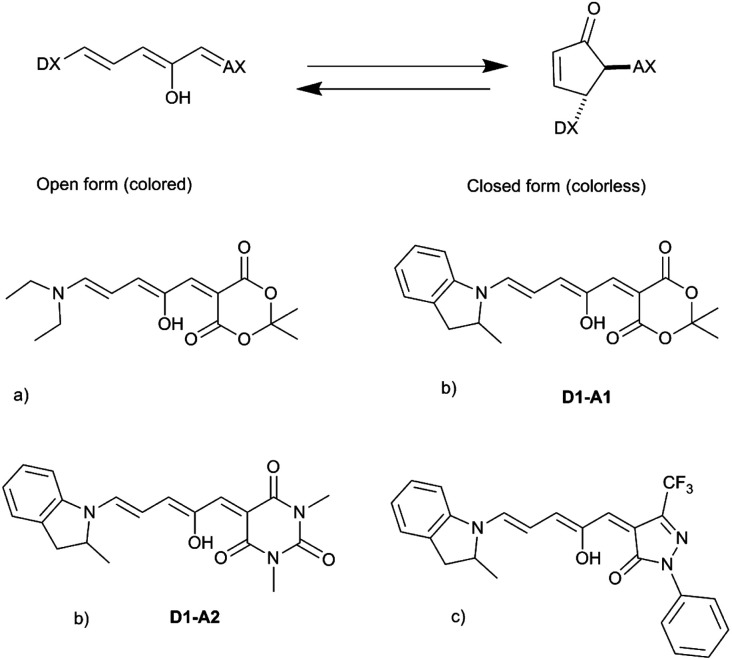
General photoswitching mechanism in DASAs and examples of synthesized DASAs from the first (a), second (b) and third (c) generations.

The synthesized DASAs are based on a push–pull system, in which a donor moiety is connected to an acceptor part through a triene bridge. Upon irradiation, the absorbed photon populates the lowest singlet excited state of the triene form (or open form), resulting in a *Z*–*E* isomerization of a double bond. Then, a thermal 4π electrocyclization is carried out to yield the cyclic form (or closed form) (see Fig. 1). This photoswitching mechanism has been extensively investigated in several recent publications.^13–20^ In DASAs, the donor and acceptor parts are mainly responsible for tuning the physical and chemical properties of the system. The first generation introduced in 2014 was designed with a secondary amine as a donor group and either Meldrum's acid (**A1**) or 1,3-dimethyl barbituric acid (**A2**) as the acceptor part.^11^ First-generation DASAs switch reversibly only in aromatic solvents such as toluene.^17^ The second generation focuses on substituting the donor part with secondary aniline-derivatives such as 2-methylindoline (**D1**).^21^ This induces a small red-shift of the absorption energy and increases the range of solvents in which the photoswitching mechanism is possible, enabling their reversible switching even in polymers.^21^ Finally, the third generation introduced new acceptor moieties, resulting in a larger red-shift and enhancing the stability of the open form. It also affects the rates of thermal equilibration by reducing the time needed to go from the closed to the open form.^22^

While many new DASAs are now available, it is still not clear how the donor and acceptor moieties influence the photophysical properties of the system and the photoswitching mechanism. This is especially true for the acceptor part, for which each substitution investigated induces major changes. In addition, some of these modifications can cause conflicting alterations in the photoswitching mechanism. In order to improve the efficacy of DASAs, the design of new acceptor moieties must be then performed in a more rational way. An important secondary goal would be to devise new DASAs that undergo photoswitching in the far-red region of the visible spectrum, which is especially important for biological applications and *in vivo* studies.^23^

In this paper, new DASAs will be investigated in a joint computational/experimental study. Starting from the known acceptor moieties, *i.e.* barbituric and Meldrum's acids, we explore new chemical structures with improved photophysical and photoswitching properties in order to rationalize their chemical behaviour.

We will first carry out a benchmark study of the recommended computational procedure to obtain vibrationally resolved spectra for DASAs. This will be followed by a frontier orbital analysis of the acceptor group that will allow us to identify and characterize new DASA candidates computationally. Finally, the synthesis of some of these compounds and their experimental characterization will be described. Our conclusions will be summarized at the end of this paper.

## Computational methods

All calculations were carried out using a development version of the ORCA quantum chemistry program package.^24^ The geometries and Hessians were obtained *in vacuo* using the B3LYP functional^25–27^ of density functional theory (DFT) with the **D3** dispersion correction.^28,29^ The same functional was used for time-dependent DFT (TDDFT) calculations. The DLPNO-STEOM-CCSD^30–35^ computations for excited states were carried out requesting five roots. The def2-TZVP basis set^36^ was used throughout with matching auxiliary basis sets.^37^ Vibrational contributions, including vibronic (Herzberg–Teller) effects, were computed using the Excited State Dynamics (ESD) module of ORCA.^38^ Further details on the various parameters and a recommended protocol are available in the ESI.†

To assess the effect of solvents, we computed DLPNO-STEOM-CCSD vertical excitation energies on 75 snapshots obtained from a molecular dynamics simulation^39^ of **D1–A1** in dichloromethane discussed in more detail in the ESI.† This explicit consideration of the solvent effect yielded results closer to the gas phase results than to results obtained with the implicit linear response Conductor-like Polarizable Continuum Model (C-PCM).^40^ Thus, gas phase results are reported throughout the rest of this paper. A similar approach has already been reported in another publication.^41^

## Results and discussion

### Benchmarking the recommended protocol

In order to get reliable predictions for the synthesis of new dyes, the use of an accurate computational method is needed. Recently, we have demonstrated the accuracy of a new wavefunction approach based on the EOM-CCSD scheme, named Domain-based Local Pair Natural Orbital Similarity Transformed Equation of Motion Coupled Clusters Singles and Doubles (DLPNO-STEOM-CCSD), for computing vertical excitation energies of organic dyes.^42,43^

**Fig. 2:**
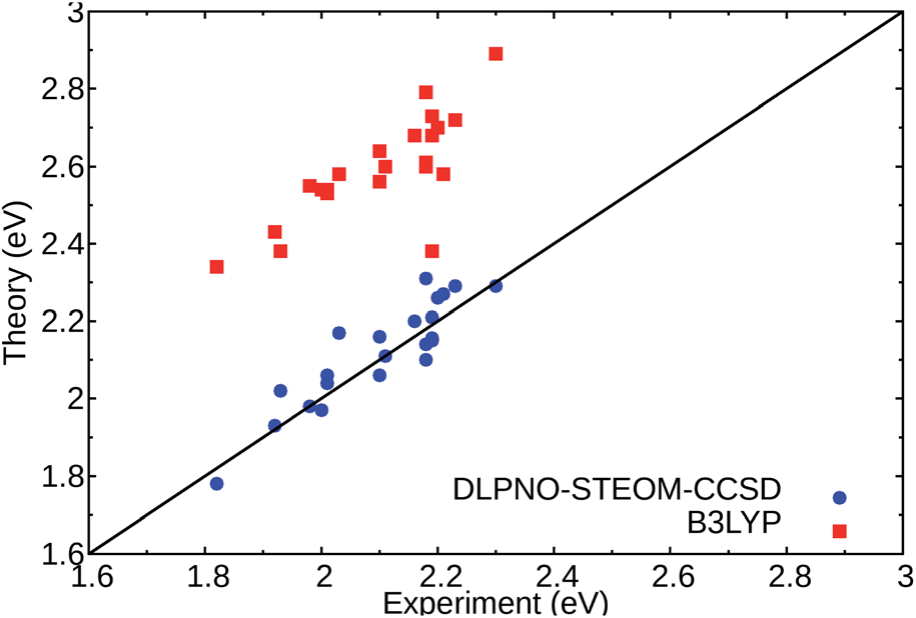
Comparison of the experimental absorption band maxima and the computed vertical excitations for the lowest **1A** → **2A** states of the selected DASAs^21,22^ (Fig. S1 and Table S1†) for DLPNO-STEOM-CCSD (blue circles) and TDDFT (B3LYP) (red squares) in gas phase. The black line signifies a perfect agreement between theory and experiment.

In this non-perturbative method, a similarity transformation is used on the EOM-CCSD Hamiltonian to reduce the computational cost without loss of accuracy.^32,33^ A class of triple excitations is also included in the method, resulting in a better description of charge-transfer systems.^32^ Finally, the use of the DLPNO scheme allows the computation of large molecules (more than 2000 basis functions) within a black box scheme. We have tested this method on a set of standard DASAs,^21,22,44^ with examples from the three generations. The predicted first vertical transitions are in good agreement with experimental measurements, with a mean average error of 0.049 eV and a standard deviation of 0.061 eV in gas phase environment. The results were also compared to a DFT functional, here B3LYP, and are shown in Fig. 2.

**Fig. 3:**
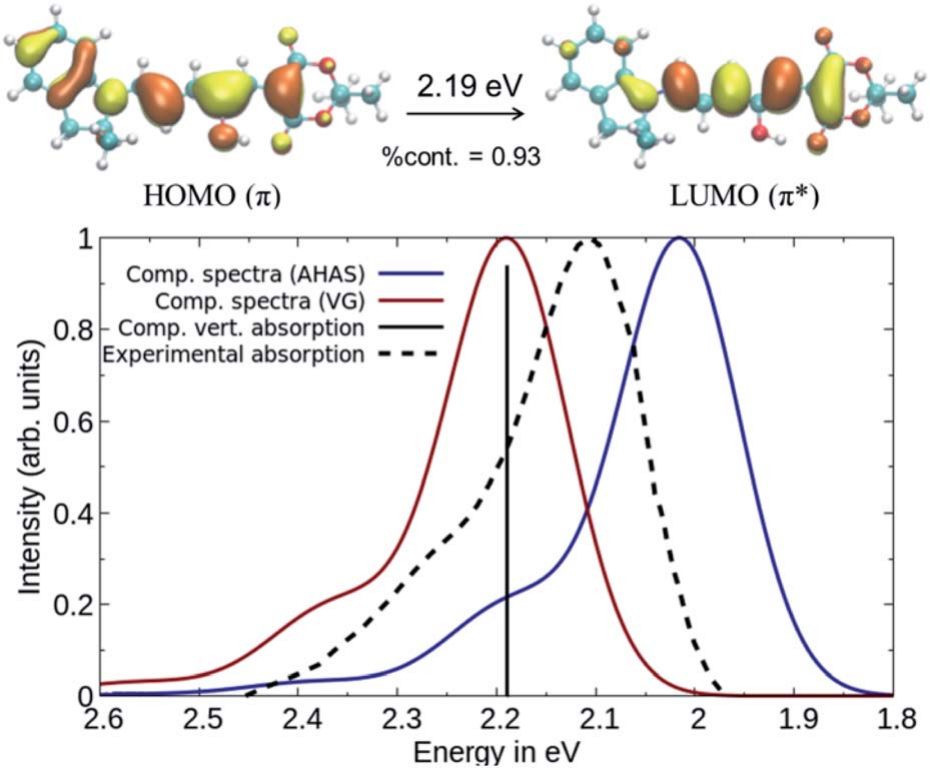
Comparison between computed and experimental absorption spectra of DASA **D1–A1**. The solid lines represent computed vibrationally resolved spectra while the dashed line corresponds to the experimental spectrum. The computed vertical excitation is represented by a vertical line.

While DLPNO-STEOM-CCSD provides a reliable description of the electronic part of the wavefunction, the vibrational contributions still need to be accounted for. Using DASA **D1–A1** as an example, the vibrationally resolved absorption spectrum corresponding to the **1A** → **2A** transition (*C*1 symmetry) between ground state (S_0_) and the first excited state (S_1_) was computed (see Fig. 3) using a time-dependent framework relying on DFT geometries and frequencies.^38^ This involves computing band shapes using the Fermi Golden Rule from wavefunctions incorporating the vibrational motion of nuclei and carrying out the integration over the radiation frequency efficiently by switching into the time domain. Based on our calculations, the first bright transition in DASAs corresponds to a delocalized π–π* excitation between S_0_ and S_1_ (see Fig. 3). The second transition is dark and higher in energy by about 2 eV. Thus, we will only compute the S_0_–S_1_ transition to model the spectrum, and spectral features will be reproduced entirely by the vibronic progression corresponding to this single electronic transition.^41^

The computed spectra in Fig. 3 were obtained using DLPNO-STEOM-CCSD transition energies and dipoles and assuming: a) the same B3LYP Hessian for both S_0_ and S_1_, but with the S_1_ geometry obtained from the S_0_ one after a single optimization step (called Vertical Gradient approach, VG) or b) different B3LYP Hessians, with the S_1_ Hessian computed at the new geometry (using the Adiabatic Hessian After Step, AHAS).^38^ In comparison with the experimental spectrum, the shape and the position of the bands are largely reproduced for both. We consider that a maximum deviation of 0.1 eV between the computed and experimental absorption is acceptable when modelling organic dyes, as written in our former papers.^42,43^ When taking into account the effect of the S_1_ Hessian, a larger red-shift of the absorption maximum is observed (0.18 eV compared to the vertical absorption). However, the absorption spectra using the VG approach are faster to compute than the AHAS variant. Thus, we have reported the VG spectra in the main text and the AHAS based spectra in Fig. S3 of the ESI.† In conclusion, the DLPNO-STEOM-CCSD method combined with a DFT level description of the vibrational contributions is an excellent tool for the prediction of the spectral properties of DASA compounds.

### Frontier orbital analysis of the acceptor group

Since the donor part of DASAs is much better studied, we decided to base our theoretical analysis on the acceptor part, while keeping the same donor moiety fixed. Our choice for the donor group has been 2-methylindoline (**D1**), often used in 2nd generation DASAs. Towards the end of our study, we will also investigate two modifications of the donor group.

To achieve a red shift, we will consider three rules of thumb here based on chemical intuition:

Rule 1. Increasing the number of double bonds in a conjugated system will lead to a red shift, in accordance with the particle in the box model.

Rule 2. Electron donating groups (EDG) destabilize and electron withdrawing groups (EWG) stabilize molecular orbitals, which can be used to affect the HOMO/LUMO gap.

Rule 3. Replacing the oxygen with an atom from the same family in a conjugated system (or in EWG/EDG that contains such a delocalized group) will lead to a destabilization/stabilization of the HOMO/LUMO.

**Fig. 4:**
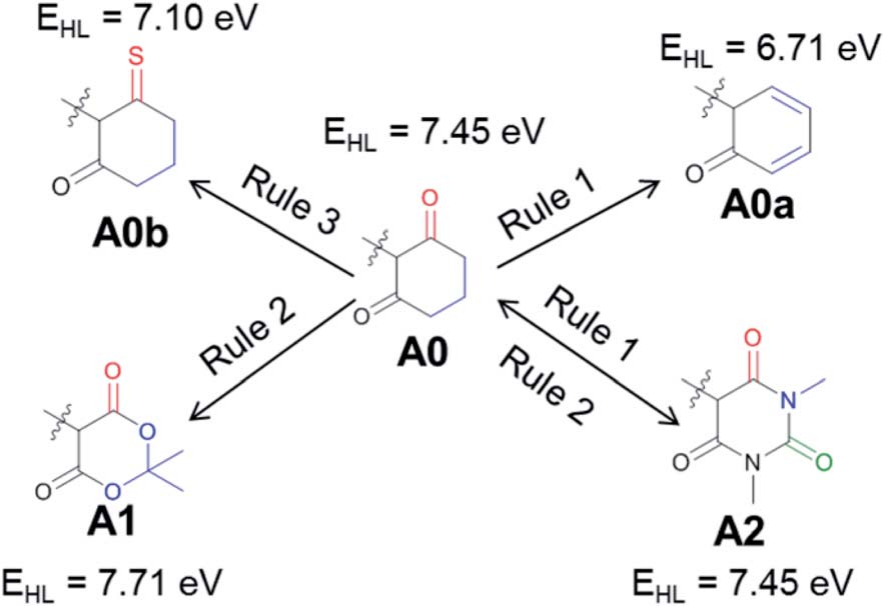
The commonly used acceptor groups **A1** and **A2** are derived from a hypothetical intermediate, **A0**, which is further modified (**A0a**, **A0b**) to illustrate general rules of achieving a red shift. *E*_HL_ designates the HOMO–LUMO gap.

**Fig. 5:**
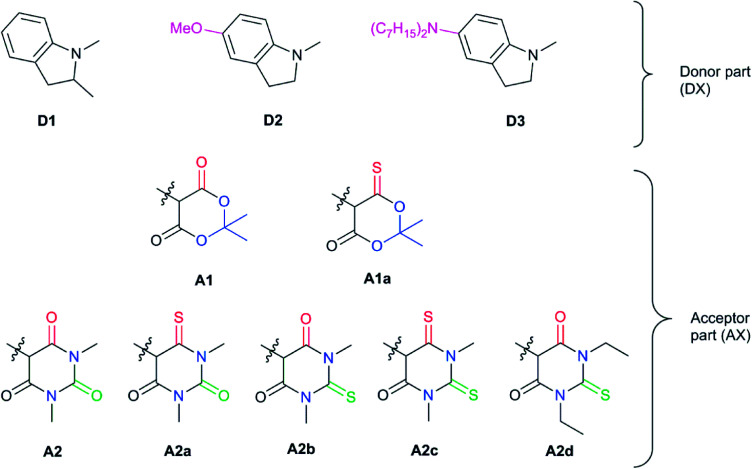
Chemical structures of Donor–Acceptor Stenhouse Adducts (DASAs) investigated in this paper. The donor parts are referenced as DX and the acceptor parts as AX with X being an index distinguishing between the species. The chemical groups modified have been highlighted using colored atoms.

Next, we will consider a model compound, **A0**, from which the common acceptor groups **A1** and **A2** can be derived in the hope that this analysis will provide insights for possible improvements. Fig. 4 illustrates how the application of Rule 1 and Rule 3 leads to a red shift in the model compound, yielding **A0a** (Rule 1) and **A0b** (Rule 3). However, when **A1** and **A2** are derived from **A0**, there is either no change (**A2**) or even a blue shift (**A1**) is observed with respect to this reference compound. For **A1**, the stabilization of the HOMO is dominant, and it is induced by the addition of an EWG. For **A2**, both orbitals are stabilized to a similar degree due to the combination of Rule 1 and Rule 2, *i.e*., the extension of the conjugated π system and the addition of an EWG. The importance of delocalization in these systems suggests the application of Rule 3 to **A1** and **A2**, yielding the compounds proposed for further investigation in Fig. 5.

In all these compounds, the carbonyl oxygen atoms have been replaced by sulfur atoms which resulted in a red shift of the HOMO–LUMO gap, suggesting the efficacy of Rule 3 in these systems. A full frontier orbital analysis of the compounds in Fig. 4 and 5 is provided in the ESI.†

### Computational design of new DASAs

Following the theoretical analysis, we are now investigating the compounds in Fig. 5, which derive from the established acceptor moieties **A1** and **A2** by the application of Rule 3. The excitation energies reported are the DLPNO-STEOM-CCSD vertical excitation energies (VEE), unless otherwise mentioned. As shown in Table 1, these values are almost identical to the maximum absorption energies (*E*_max_) computed using the VG scheme, the largest deviation being 0.03 eV. This is consistent with the small displacement found between S_1_ and S_0_ geometries in all molecules.

**Table 1:** The dominant vertical transition between the ground state and the first excited state of DASA dyes studied in the work (weight above 94%). The vertical excitation energies (VEE) have been computed using the DLPNO-STEOM-CCSD method together with the def2-TZVP basis set, requesting 5 roots. The computed maximum absorption energy (*E*_max_) corresponds to the maximum of the computed absorption spectrum, using the VG approximation. All the values listed are in eV

**1A** → **2A** transition	Comp. VEE (eV)	Comp. *E*_max_ (eV)	Exp. *E*_max_ (eV) in CH_2_Cl_2_
**D1–A1**	2.19	2.18	2.1
**D1–A1a**	1.88	1.89	—
**D1–A2**	2.00	1.97	2.02
**D1–A2a**	1.75	1.75	—
**D1–A2b**	1.89	1.89	—
**D1–A2c**	1.64	1.64	—
**D1–A2d**	1.84	1.85	1.92
**D2–A2d**	1.80	1.80	1.88
**D3–A2d** a	1.72	1.72	1.77

a For the computational results N(Me)_2_ was considered instead of N(C_7_H_15_)_2_ in the donor part.

Starting with the Meldrum's acid acceptor moiety (**A1**), substituting the appropriate oxygen by a sulfur atom yields **A1a**. The computed VEE value shows an impressive red-shift of nearly 0.3 eV (Table 1), while the difference density plots between S_1_ and S_0_ (DDP) (Fig. S4†) for **A1** and **A1a** show a better delocalization of the electronic density in **A1a**. The changes in the atomic Mulliken charges of the atoms in the acceptor moiety further corroborate Rule 3. In particular, the partial charge of the carbon bonded to the sulfur atom has decreased by about 0.2 e, indicating a more evenly distributed electronic density extending to the carbon atom. DASA **D1–A1a** seems to be a valuable candidate, but as will be discussed below, there are better candidates for synthesis.

**Fig. 6:**
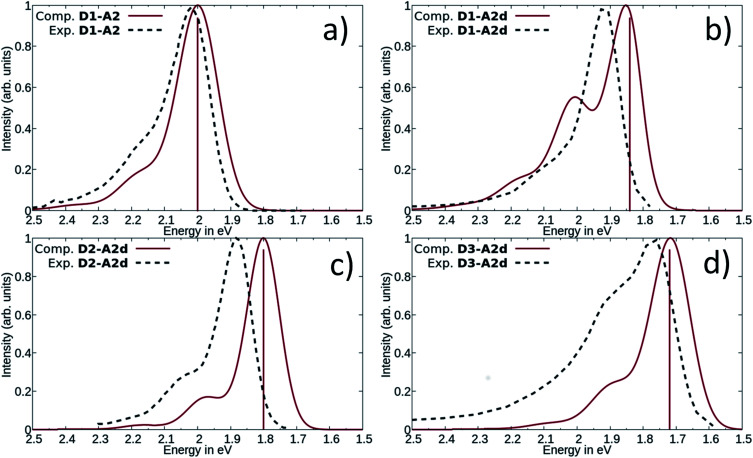
Comparison between computed and experimental absorption spectra of DASAs, **D1–A2** (a), **D1–A2d** (b), **D2–A2d** (c) and **D3–A2d** (d). The solid lines represent computed vibrationally resolved spectra while the dashed lines correspond to experimental spectra. The computed vertical excitation energies are represented by vertical lines.

Next, we have also investigated the well-studied barbituric acid acceptor moiety (**A2**). In comparison to **A1**, the conjugated π system is extended to the two nitrogen atoms, leading to a red-shift of the maximum absorption energy of 0.08 eV experimentally. For the computed *E*_max_ value, the red-shift is larger, about 0.17 eV (see Fig. 6 and Table 1). However, by looking at the DDP (Fig. S4†), it is evident that there is a competition between two electronic effects in the barbituric acid part, namely the length of the π-conjugated pathway and the presence of acceptor moieties (Rule 1 and Rule 2). The application of Rule 3 yields three new acceptor moieties in which we substitute either one oxygen atom in position 2 (**A2a**), one in position 4 (**A2b**), or both at the same time (**A2c**), see Fig. 5. The fourth acceptor moiety **A2d** has been synthesized and discussed later.

The substitution of oxygen by sulfur atoms by Rule 3 causes the density difference plots to change dramatically. Upon excitation, the electronic density increases on oxygen atoms while it decreases when they are substituted by sulfur atoms. This modification leads to a larger electron density on the carbons in the acceptor moiety and, thus, to a stabilization of the LUMO orbitals. Again, the changes in the electron density are reflected in partial charges which decrease by about 0.2 to 0.3 e on the carbon adjacent to the heteroatom, approaching neutrality. These effects are more important for DASA **D1–A2a** due to a closer proximity to the triene bridge (and the donor part). As a result, the computed DLPNO-STEOM-CCSD vertical excitation energies are red shifted upon substitution to an extent of about 0.25 eV when the oxygen atom in position 2 is substituted (**A2a**), 0.11 eV for the oxygen atom in position 4 (**A2b**) and 0.36 eV when both substitutions are made (**A2c**), which is the best red shift value among the species studied.

### Synthesis and photophysical properties of the newly designed DASAs

**Fig. 7:**
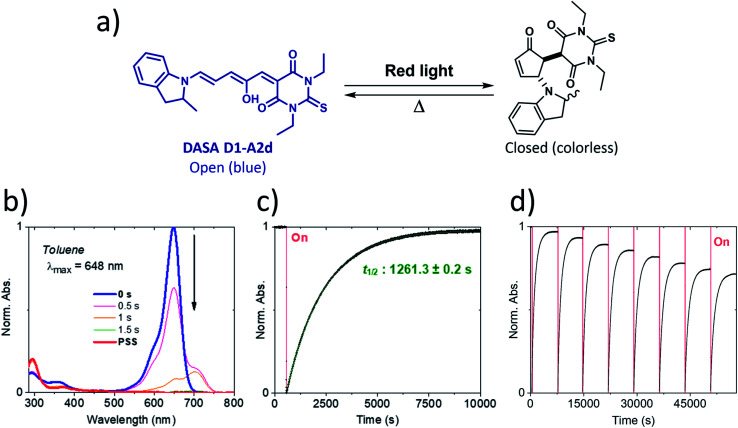
Photochromism of DASA **D1–A2d** in toluene. Irradiation with red light leads to a quantitative photobleaching followed by a nearly quantitative thermal back-reaction at 20 °C. (a) Chemical structures of both isomers. (b) Full absorption profile under red light irradiation until the photostationary state (PSS) is reached. (c) Photochromic behavior showing peak absorption over time (red light irradiation is represented by a red block). (d) Fatigue resistance study.

Our calculations showed that the replacement of oxygen by sulfur atoms (Rule 3) in 1,3-disubstituted barbituric acid (**A2**) provides a large red-shift of the absorption spectra of the resulting DASAs. In order to check the optical properties of these newly designed dyes, we chose the acceptor **A2d** as the precursor is a commercially available compound. The resulting DASA **D1–A2d** was efficiently synthesized and its photochromic behavior was studied in detail (see Fig. 7).

The measured experimental maximum absorption energy reaches 1.92 eV (647 nm) in dichloromethane, corresponding to a reasonable red-shift of 0.14 eV compared to the initial DASA **D1–A2** (615 nm). The computed VEE is in good agreement with the experiment as well as the computed vibrationally resolved spectra (see Fig. 6 and S3†) with a difference of 0.06 eV between the experimental and computed *E*_max_ values.

The photochromic behavior of DASA **D1–A2d** was studied by pump-probe absorption spectroscopy showing excellent photoswitching properties, especially when compared with its analogue **D1–A2** (see Fig. 7 for toluene and Fig. S10† for other solvents). In toluene, irradiation of both DASAs leads to full photobleaching followed by a near quantitative thermal relaxation for **D1–A2d** (≈97%, *t*_1/2_ ∼ 1261 s), whereas no return to the open isomer is observed for **D1–A2**.^22^ A video comparing the relaxation timing of the two compounds is available in ESI.† In terms of fatigue resistance, approximately 3–5% permanent bleaching is observed per cycle. It is a surprisingly good result as a former DASA featuring the 1,3-diethylthiobarbituric moiety was reported quite unstable.^45^

Encouraged by the interesting photoswitching properties of **D1–A2d**, we explored new thiobarbituric acid-based DASAs with different donor groups. More precisely, the incorporation of electron donating groups at the *para*-position of the indoline, such as methoxy (**D2**) or *N*,*N*-diheptylamine (**D3**), leads to larger bathochromic shifts compared to **D1** as well as an accelerated reverse reaction^21^ (see Table 2 and Fig. S6†). However, the higher basicity of these electron-rich indolenines limits the photochromism to less polar solvents.^21^ The measured *E*_max_ value is 1.88 eV (659 nm) for **D2–A2d** and 1.77 eV (702 nm) for **D3–A2d** in dichloromethane.

**Table 2:** Comparison of absorption maxima between the new thiobarbituric acid-based DASAs and the barbituric acid-based analogues (in dichloromethane)^21,22^

Experimental absorption maxima for **1A** → **2A** transition eV (nm)	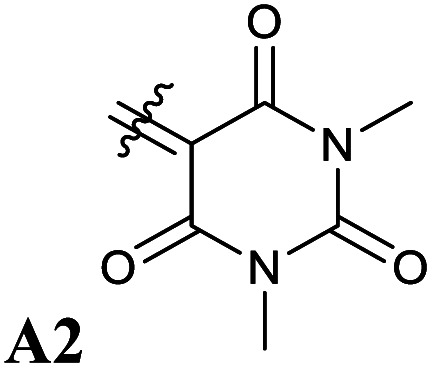	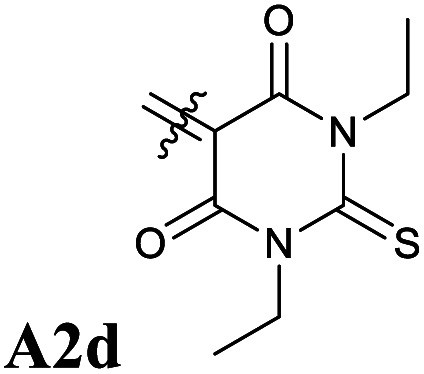
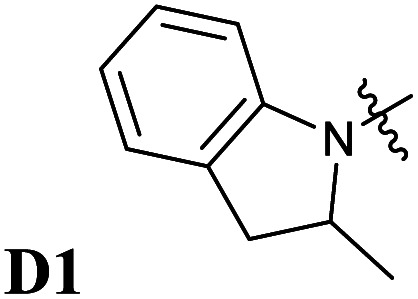	**D1–A2**	**D1–A2d**
	2.02 (615)	1.92 (647)
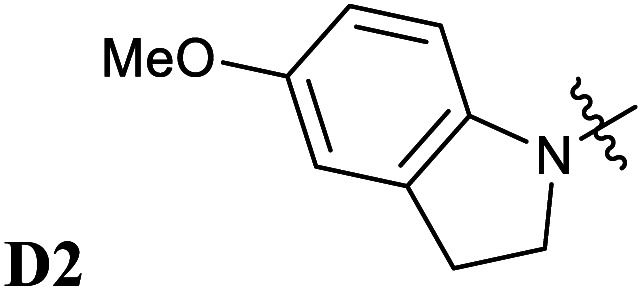	**D2–A2**	**D2–A2d**
	1.97 (629)	1.88 (659)
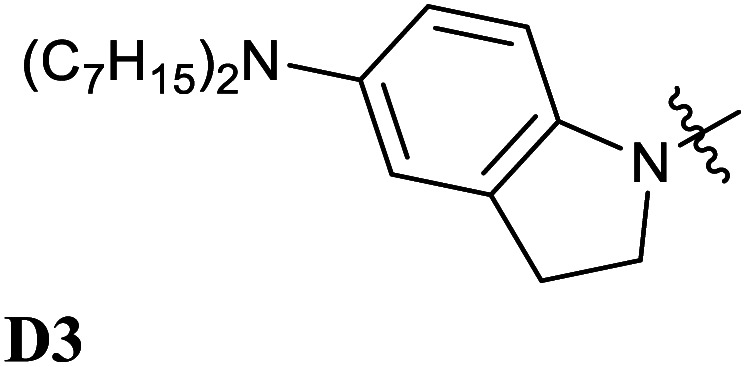	**D3–A2**	**D3–A2d**
	1.85 (669)	1.77 (702)

The computed absorption spectra are also in good agreement with the experimental spectra (see Fig. 6), and correctly reproduce the red-shift induced by the new donor moieties.

Thus, the newly designed acceptor moiety **A2d** causes a consistent red-shift in the absorption for every donor moiety considered. In terms of electronic structure, adding a methoxy or an amine group to the indoline part extends the conjugated π system of the DASA, and increases the electronic density transferred to the acceptor moiety upon light absorption. This can be seen on the DDP plot in Fig. S4.†

**Fig. 8:**
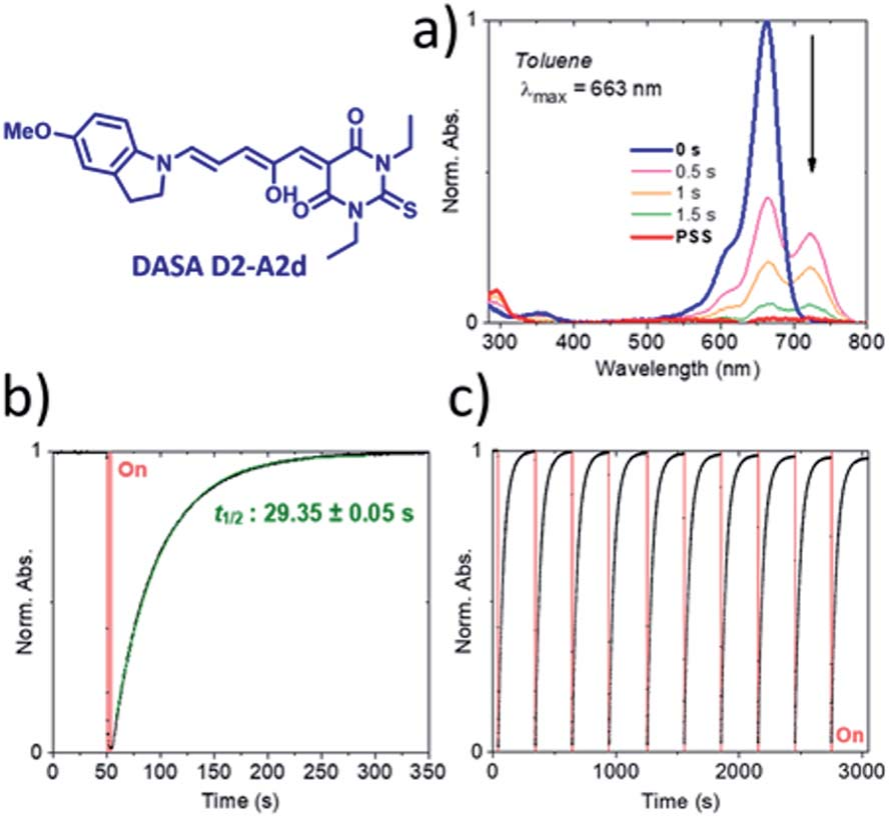
Pump–probe absorption spectroscopy of DASA **D2–A2d** in toluene at 20 °C. (a) Full absorption profile under red light irradiation until the photostationary state (PSS) is reached. (b) Peak absorption over time (red light irradiation is represented by a red block). (c) Cycling study showing less than a 3% decrease in absorption over ten photoswitching cycles.

The photochromic behavior of DASAs **D2–A2d** and **D3–A2d** was also studied using pump–probe absorption spectroscopy. Upon irradiation in toluene, DASA **D2–A2d** quantitatively cyclizes to the closed form and then rapidly opens to give back the linear isomer (≈100%, *t*_1/2_ ∼ 29 s) (see Fig. 8 for toluene and Fig. S12† for other solvents). Furthermore, DASA **D2–A2d** is very fatigue resistant showing less than 0.3% decomposition per cycle, which together with its absorption beyond 700 nm, makes it a very promising candidate for future applications.

**Fig. 9:**
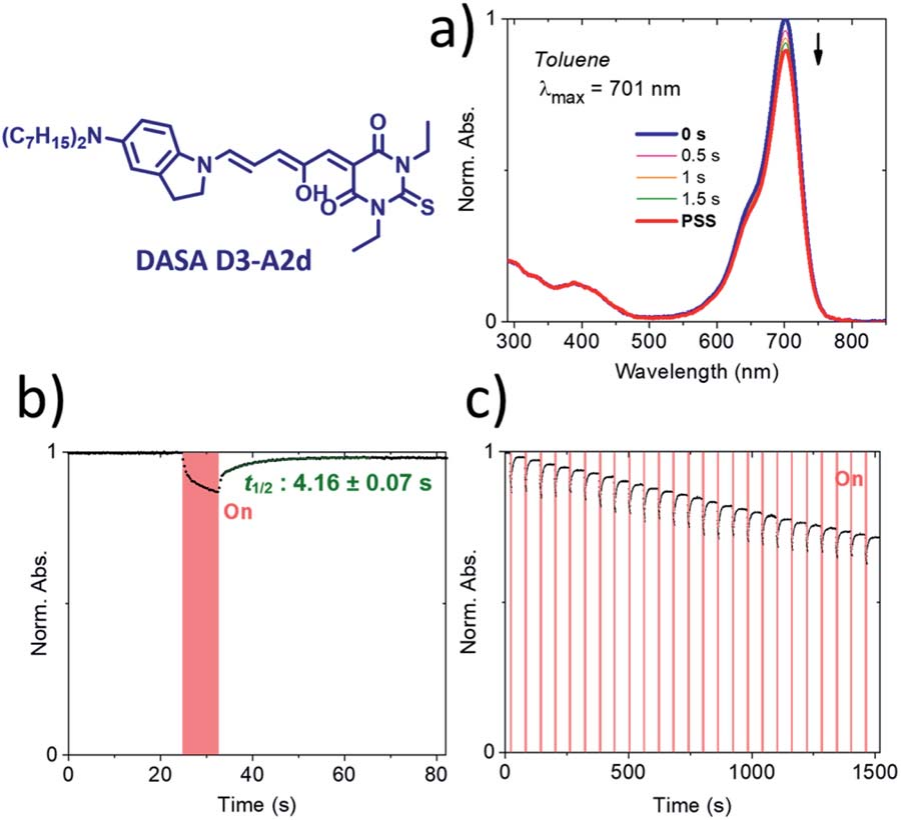
Pump–probe absorption spectroscopy of DASA **D3–A2d** in toluene showing a partial response to red light (20 °C). (a) Full absorption profile under red light irradiation until the photostationary state (PSS) is reached. (b) Peak absorption over time (red light irradiation is represented by a red block). (c) Cycling study.

To the best of our knowledge, compound **D3–A2d** is the second most red-shifted DASA synthesized to date.^22^ However, pump–probe absorption spectroscopy shows that both cyclization and thermal reversion are only partial (see Fig. 9 for toluene and Fig. S14† for other solvents). In contrast, **D2–A2d** features excellent photoswitching properties, although its band maximum is slightly blue-shifted. Finally, NMR studies show that the thermal equilibrium of the new synthesized DASAs are above 90% (100% for **D2–A2d** and **D3–A2d**) in favor of the open form in CD_2_Cl_2_. This is a clear improvement in comparison to previous DASAs incorporating the barbituric acid acceptor part.^21^

These results clearly underscore the relevant properties of the new acceptor moieties designed by this approach and its potentiality in the preparation and use of a new generation of DASAs.

## Conclusion

New DASAs with improved photoswitching properties and activity under red light were obtained thanks to a joint computational/experimental approach.

First, the computational procedure followed is able to accurately model and reproduce the photophysical properties of previously synthesized DASAs. Hence, we have investigated new red-shifted photoswitch compounds by modifying both acceptor and donor moieties. On the basis of a frontier orbital analysis, the well-studied barbituric acid acceptor part was modified by substituting one or several oxygen atoms with sulfur. Vibrationally resolved DLPNO-STEOM calculations were then used for the quantitative prediction of red shifts in these compounds. Then, DASA **D1–A2d** was synthesized and the experimental and computational results were found to agree. In addition, this compound showed an improved photochromism behavior compared to its analogue **D1–A2**.

We have also investigated the impact of modifying the electron-donor moiety on the donor part of DASAs. Two new DASAs were synthesized with an absorption reaching the near-infrared part of the visible spectrum. Interestingly, DASA **D2–A2d** shows excellent photoswitching properties combining a notably red-shifted absorption and a great response to light in toluene.

With the success of this joint approach, we plan to design new DASAs with improved properties to further expand the range of applicability of these dyes.

## Conflicts of interest

There are no conflicts to declare.

## Supplementary Material

SC-012-D0SC06575G-s001

SC-012-D0SC06575G-s002
