# Supplementary material for: Redesigning donor–acceptor Stenhouse adduct photoswitches through a joint experimental and computational study
Source: Chem Sci. 2021 Jan 4;12(8):2916–24. doi: 10.1039/d0sc06575g (PMC8179403; doi:10.1039/d0sc06575g)
Supplement: SC-012-D0SC06575G-s002 [file SC-012-D0SC06575G-s002.pdf]

Redesigning Donor-Acceptor Stenhouse Adduct Photoswitches through a joint experimental and computational study

Romain Berraud-Pache[ab,‡], Eduardo Santamaría-Aranda[c,‡], Bernardo De Souza[d], Giovanni Bistoni[a], Frank Neese[a], Diego Sampedro[c]* and Róbert Izsák[a]*

[a]Max-Planck-Institut für Kohlenforschung, Kaiser-Wilhelm Platz 1, 45470 Mülheim an der Ruhr, Germany

[b] Sorbonne Université, Laboratoire d'Archéologie Moléculaire et Structurale, CNRS UMR 8220, UPMC – Tour 23, 3ème étage, couloir 23-33, BP 225, 4 place Jussieu, 75005, Paris, France

[c]Departamento de Química, Centro de Investigación en Síntesis Química (CISQ), Universidad de La Rioja, Madre de Dios 53, E-26006 Logroño, Spain

[d]FAccTs GmbH, Rolandstrasse 67, 50677 Köln, Germany

‡ These two authors contributed equally.

Table of Contents

Computational Methods 3

Benchmarking the recommended protocol 3

AHAS vibronic spectra 8

Frontier Orbital Analysis of the Acceptor Group 9

Molecular Orbitals and Difference Densities Plots 16

Experimental procedures 22

General pump-probe absorption procedure 22

Light emitting diodes for irradiation 23

Synthetic procedure 24

Characterization data 27

Comparison of absorption spectra of DASAs D1-A2 and D1-A2d 27

Comparison of absorption spectra of DASAs D1-A2d, D2-A2d and D3-A2d 28

Solvatochromic effect 29

Molar absorptivity 29

Pump-probe absorption experiments of DASA D1-A2d 30

Pump-probe absorption experiments of DASA D2-A2d 32

Pump-probe absorption experiments of DASA D3-A2d 34

Thermodynamic equilibrium NMR studies 37

NMR and HRMS spectra 38

Cartesian Coordinates 46

References 56

Computational Methods

All calculations were carried out using a development version of the ORCA quantum chemistry program package.[1] The geometries of the ground state were optimized in vacuo using the B3LYP functional[2-3] with the def2-TZVP basis set[4] and the D3 dispersion correction.[5-6] The DLPNO-STEOM-CCSD[7-12] calculations were performed using the def2-TZVP basis set[4] with matching auxiliary basis sets[13] requesting 5 roots and with the following thresholds: "TightPNO" settings,[14] the $T_{CutPNOsingles}$ keyword set to $6.6x10^{-10}$ and the active space selection keywords "Othresh" and "Vthresh" set to $5.0x10^{-3}$. The TD-DFT calculations were performed using the B3LYP functional using the def2-TZVP basis set with matching auxiliary basis sets[13] requesting 3 roots. The Conductor-like Polarizable Continuum Model (C-PCM)[15] was used to model implicit solvent effects, *i.e.*, dichloromethane (CH_2Cl_2) and chloroform ($CHCl_3$) during DLPNO-STEOM-CCSD and TD-DFT calculations. For all dyes, the computed energies for the $S_0 \rightarrow S_1$ transitions are associated with the HOMO→LUMO transition (weight above 94%). The spectra were computed using the ESD module. The Adiabatic Hessian After Step (AHAS) and the Vertical Gradient (VG) approaches[16] were used to obtain the excited state geometries and Hessians in vacuum. The general workflow is: first the geometries of the ground state were optimized in a previous DFT calculation followed by a frequency calculation; then, the 1st excited states geometries (or 2nd for DASA **D1-A2d**) were predicted starting from the GS optimized one, performing one augmented Hessian step on the ES PES; and finally, for VG the GS frequencies were used to compute the vibronic progression while for AHAS the ES frequencies were computed at the new position. The DLPNO-STEOM-CCSD method was then used to compute the vertical excitations energies and transition dipoles on the ground state geometry.[17] The vibronic effects (or Herzberg-Teller effects, HT) were also included. Note that the size of the displacement vector is monitored during the computation and the Excited State Dynamics (ESD) calculation is aborted if it is too large. The spectra were modelled using a Gaussian line shape and at 298K. The INLINEW keyword was applied in order to match the experimental Full Width at Half Maximum (FWHM). The default settings were used for all other parameters.

The molecular dynamics (MD) simulation was performed using the semi-empirical extended tight-binding method GFN2-xTB method[18] and available in ORCA.[1] A box of 8 Å of dichloromethane molecules was added around the **D1-A1** DASA, yielding a system of 637 atoms. Then a MD was performed for 50 ps at 298 K using the default parameters. Due to equilibration, the snapshots were extracted after 12.5 ps of MD. No further optimization was performed and the vertical excitation energy was computed using the parameters listed above.

Benchmarking the recommended protocol

In order to give relevant predictions for the synthesis of new dyes, the use of an accurate computational method is needed. Several publications have already pursued this goal but the commonly used TD-DFT method fails at reproducing the photophysical properties. Wavefunction approaches like CIS(D) and CASPT2 have also been tried, with more success.[19-20] However, these methods were less successful for the second and third DASAs generation. To tackle this problem, we have recently shown the accuracy of a new wavefunction approach based on the EOM-CCSD scheme. The DLPNO-STEOM-CCSD method yields very precise vertical excitation energies of usual organic molecules and difficult cases like BODIPY dyes.[17]

To sample this method we have investigated molecules recently synthesized by Alaniz et al.,[21-22] where examples from the three generations of DASAs are reported (Figure S1). The computed vertical absorption energies using DLPNO-STEOM-CCSD with and without implicit solvation has excellent agreement with the measured maximum absorption (Table S1). The use of implicit solvation, here chloroform, yielded a Mean Average Error of 0.146 eV with all results red-shifted compared to the experimental ones. On the other hand, gas phase DLPNO-STEOM-CCSD results have a MAE of 0.048 eV, but the results can be either blue or red-shifted.

We have also computed the vertical excitation energies using an explicit solvation model. The use of an explicit scheme is supposed to be better at reproducing the solvent effect, as more interactions are taken into account, like hydrogen-bonds or the geometric deformation induced by the solvent. To test this hypothesis, we have also added explicit molecules of dichloromethane for the **D1-A1** DASA, performed molecular dynamics and compute the vertical excitation energies on 75 snapshots extracted from the MD. The computed absorption spectrum is then obtained using a gaussian distribution. Surprisingly, we have observed that the computed maximum absorption using explicit solvent molecules is closer to the computed vertical excitation energy in gas phase than the one computed using an implicit solvation, as reported in a previous publication.[23] Thus, we have decided to report only gas phase results in the manuscript.

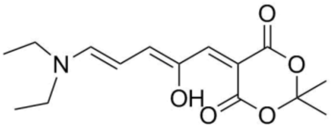

1-S8

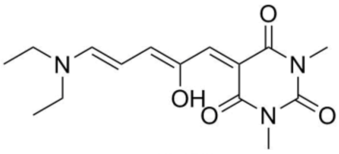

2-S8

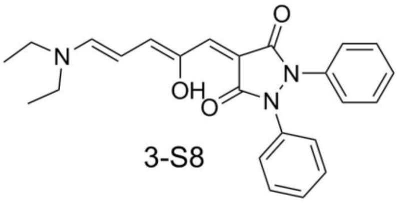

3-S8

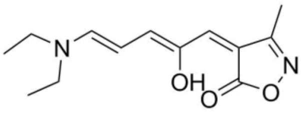

4-S8

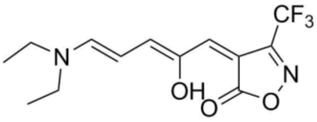

5-S8

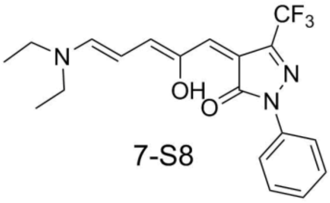

7-S8

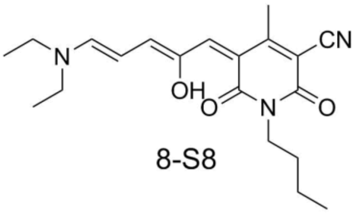

8-S8

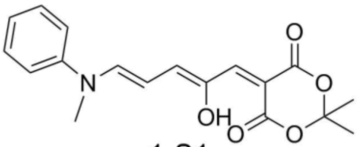

1-S1

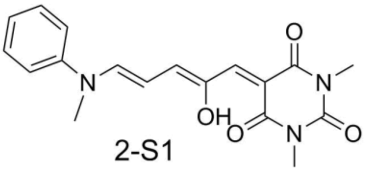

2-S1

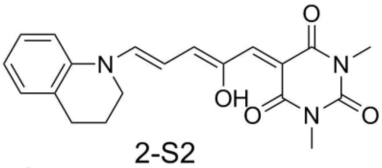

2-S2

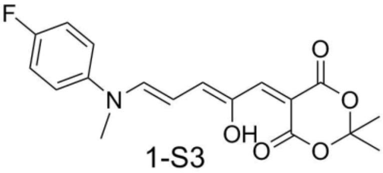

1-S3

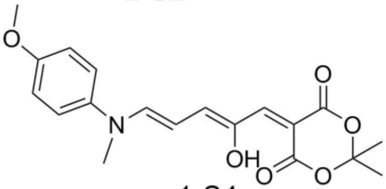

1-S4

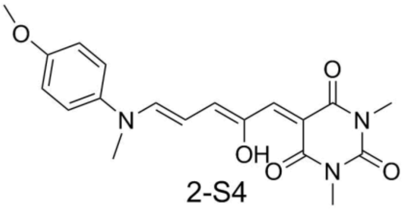

2-S4

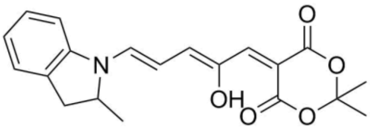

1-S7

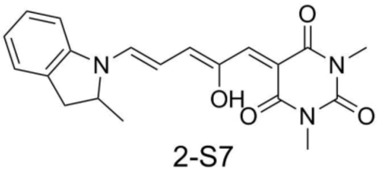

2-S7

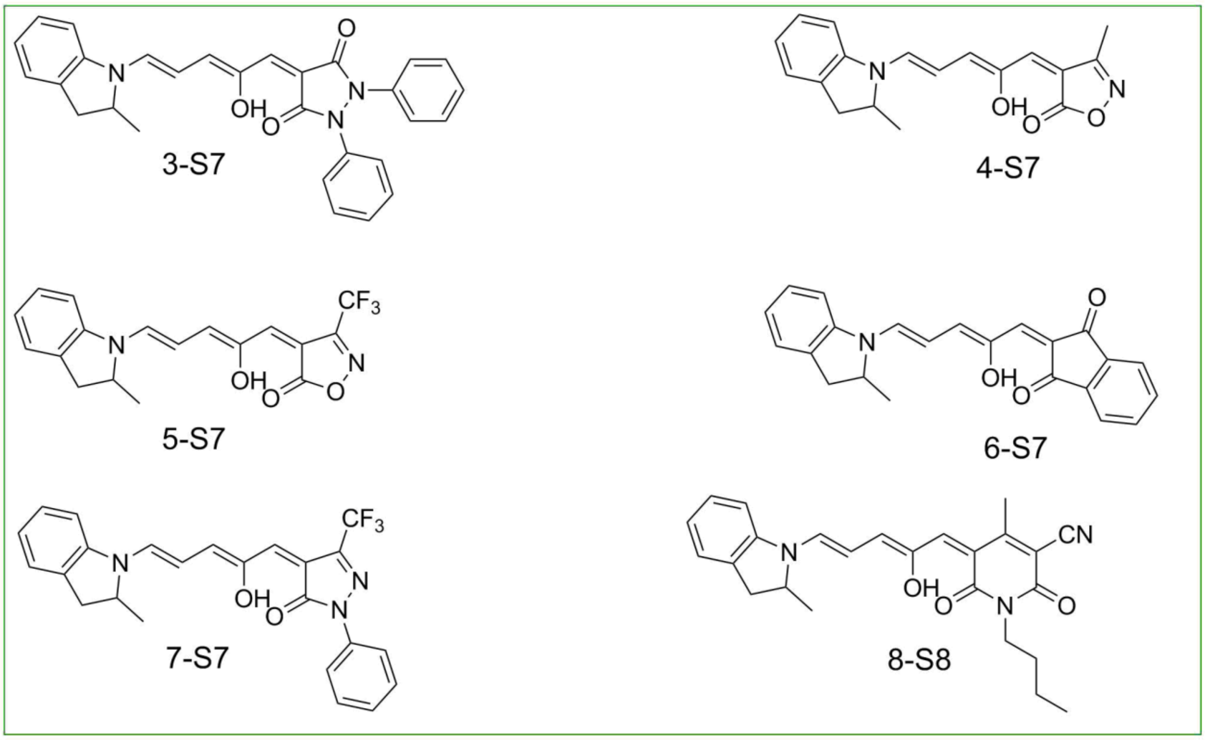

Figure S1. Structure of the DASA dyes investigated. The red square corresponds to 1st generation DASAs, the blue one to 2nd generation and the green one to 3rd generation.

Table S1. Dominant bright computed vertical transition to the first excited state of DASA dyes from Figure S1 using several methods. The def2-TZVP basis set was used requesting 3 roots for TDDFT and 5 roots for DLPNO-STEOM-CCSD. All the values listed are in eV. The red cells correspond to 1st generation DASAs, the blue ones to 2nd generation and the green ones to 3rd generation.

**Vertical absorption S_0->S_1 eV (nm)**	**STEOM-CPCM (in $CHCl_3$)**	**STEOM gas phase**	**Exp. ($CHCl_3$)[21]**	**B3LYP-CPCM (in $CHCl_3$)**	**B3LYP-gas phase**	**CASPT2 gas phase[20]**	**SOS-CIS(D) gas phase[19]**
1-S8	2.16 (574)	2.29 (541)	2.30 (539)	2.89 (429)	2.89 (429)	2.47 (502)	2.14 (579)
2-S8	2.01 (617)	2.15 (577)	2.19 (566)	2.75 (451)	2.73 (454)		2.03 (611)
3-S8	2.04 (608)	2.21 (561)	2.19 (566)	2.79 (445)	2.68 (388)		
4-S8	2.09 (593)	2.31 (537)	2.18 (569)	2.82 (440)	2.79 (444)		
5-S8	2.05 (606)	2.156 (575)	2.19 (566)	2.79 (444)	2.38 (521)		
7-S8	1.95 (636)	2.06 (602)	2.10 (690)	2.63 (471)	2.56 (484)		
8-S8	1.81 (685)	1.97 (629)	2.00 (620)	2.58 (481)	2.54 (488)		
1-S1	2.09 (610)	2.27 (546)	2.21 (561)	2.35 (527)	2.58 (480)		
2-S1	1.98 (626)	2.14 (579)	2.18 (569)	2.41 (515)	2.61 (475)		
2-S2	1.96 (633)	2.10 (590)	2.18 (570)	2.39 (519)	2.60 (477)		

1-S3	2.10 (590)	2.29 (541)	2.23 (557)	2.51 (494)	2.72 (456)		
1-S4	2.06 (602)	2.26 (549)	2.20 (563)	2.48 (500)	2.70 (459)		
2-S4	1.95 (636)	2.11 (588)	2.11 (588)	2.38 (521)	2.60 (477)		
1-S7	1.97 (629)	2.16 (574)	2.10 (590)	2.63 (471)	2.64 (470)		
2-S7	1.86 (667)	2.04 (608)	2.01 (617)	2.45 (506)	2.54 (488)	2.25 (551)	
3-S7	1.86 (667)	2.06 (602)	2.01 (617)	2.53 (490)	2.53 (489)		
4-S7	1.93 (642)	2.17 (571)	2.03 (611)	2.59 (479)	2.58 (480)		
5-S7	1.83 (678)	1.98 (626)	1.98 (626)	2.54 (488)	2.55 (487)		
6-S7	1.81 (685)	2.02 (614)	1.93 (642)	2.27 (546)	2.38 (521)	2.19 (566)	
7-S7	1.78 (697)	1.93 (642)	1.92 (646)	2.43 (510)	2.43 (510)	2.16 (574)	
8-S7	1.62 (765)	1.78 (697)	1.82 (681)	2.37 (523)	2.34 (530)		

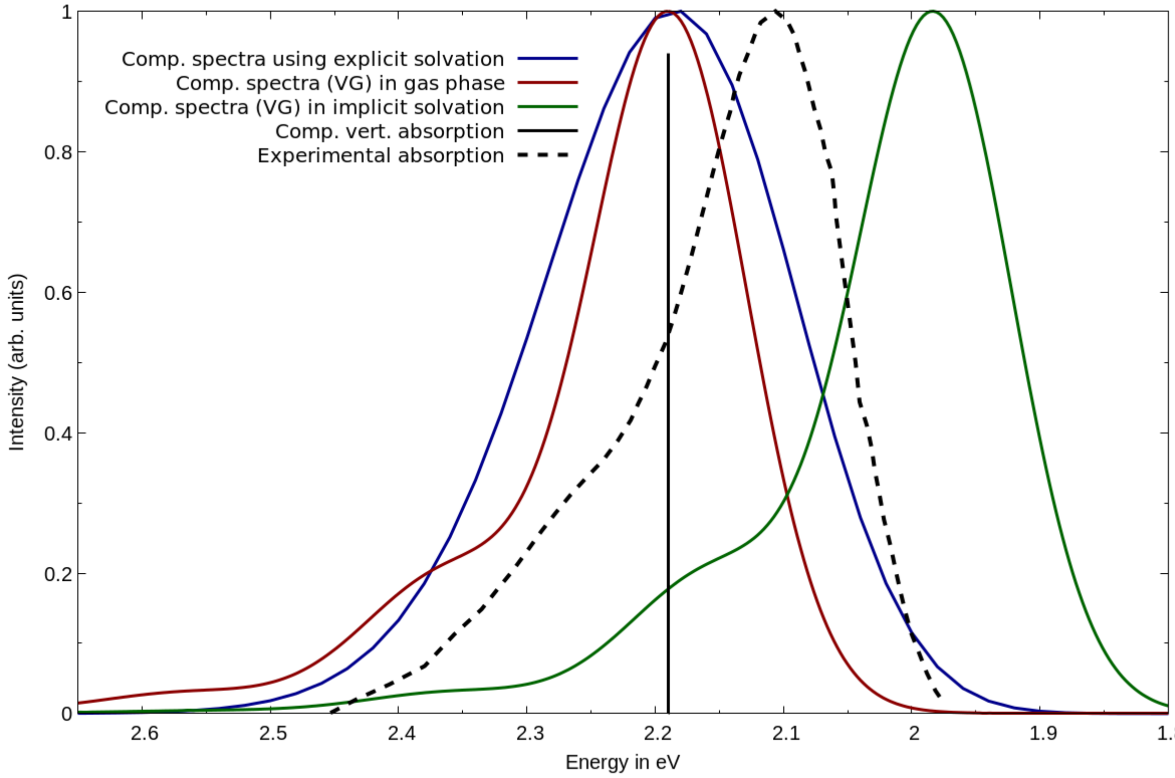

Figure S2. Comparison between computed and experimental absorption spectra of DASA **D1-A1** using different types of solvation. In blue, Gaussian distribution of the vertical excitation of 75 snapshots where the solvent has been taken into account explicitly. In red, computed spectrum using the VG algorithm in gas phase. In green, computed spectrum using the VG algorithm with the vertical excitation computed using an implicit solvation scheme (CPCM). The solid lines represent computed vibronically resolved spectra while the dashed line corresponds to experimental spectra.

AHAS vibronic spectra

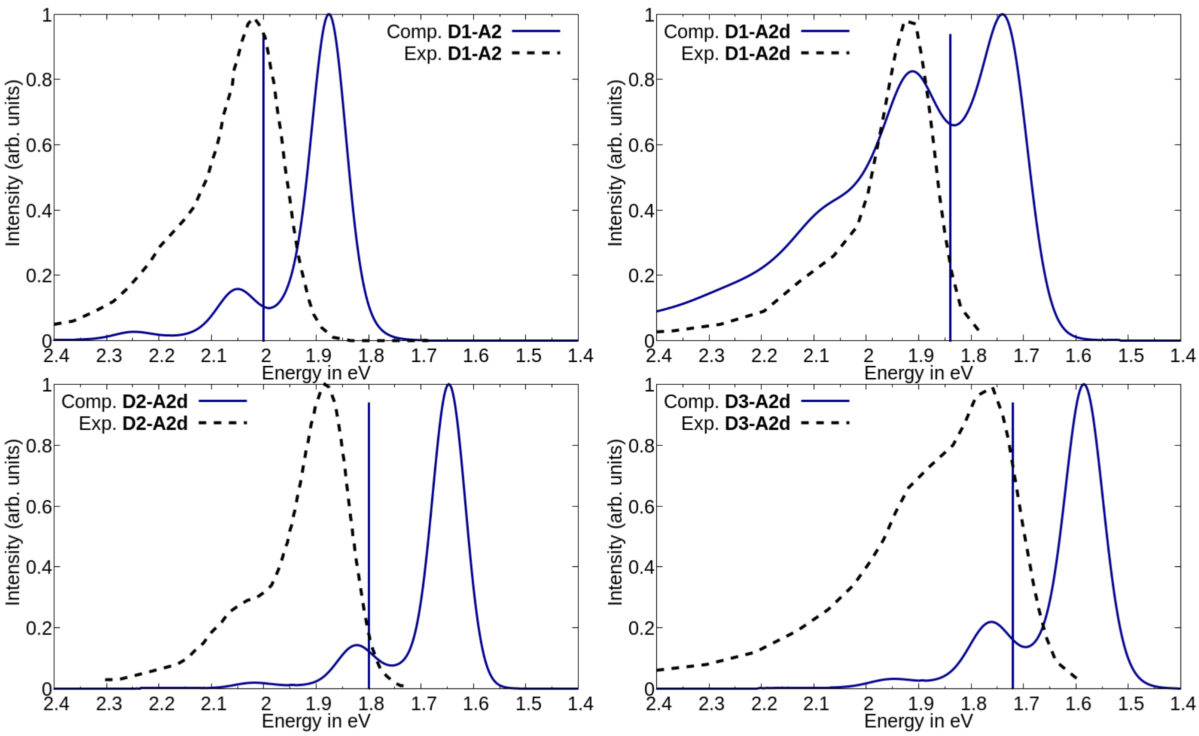

Figure S3. Comparison between computed and experimental absorption spectra of DASAs when using the AHAS algorithm. The solid lines represent computed vibronically resolved spectra while the dashed lines correspond to experimental spectra.

Frontier Orbital Analysis of the Acceptor Group

1. Principles

To achieve a red shift, there are a few general principles that need to be considered:

Rule 1: Increasing the number of conjugated double bonds will lead to a red shift, in accordance with the particle in the box model, in which the energy difference between the first two states is inversely proportional to L^2, where L is the length of the box, *i.e.*, the spread of the conjugated system.

Rule 2: Adding electron withdrawing groups (EWG) or electron donating groups (EDG) to positions where they may interact with orbital densities will stabilize (in the EWG case) or destabilize (in the EDG case) those orbitals. For a red shift, the HOMO (or another dominant orbital) needs to be destabilized and/or the LUMO (or another dominant orbital) needs to be stabilized.

Rule 3: The role of heteroatoms in a conjugated system (or in EWG/EDG that contains such a delocalized group). Consider the following sequence of molecules for small n=0,1,2…:

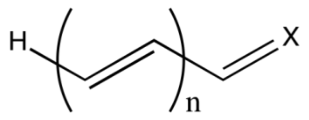

Since X is part of the conjugated system, both HOMO and LUMO will be affected by the substitution. When compared with X=CH_2, both X=O and X=S stabilize both the HOMO and the LUMO, but to a different extent. Thus, X=O leads to a blue shift, X=S leads to a red shift with respect to X=CH_2 (certainly until n=2, although the shift becomes increasingly smaller). When comparing X=O and X=S with each other, it becomes clear that X=S stabilizes the LUMO more and destabilizes the HOMO more than X=O. Thus, replacing O with S often leads to a red shift and can be the basis of a systematic investigation. For example, if n=2, the HOMO-LUMO gap is 10.99 eV for X=O and 9.93 eV for X=S. (Using Se instead of S does not have much effect, at least for the purposes considered here).

2. Donor-Acceptor Systems

2.A. A Model System

We begin by considering a model compound for these systems, **A0**, which has no experimental relevance in itself, but as we will see later on, two commonly used acceptor groups can be derived from it:

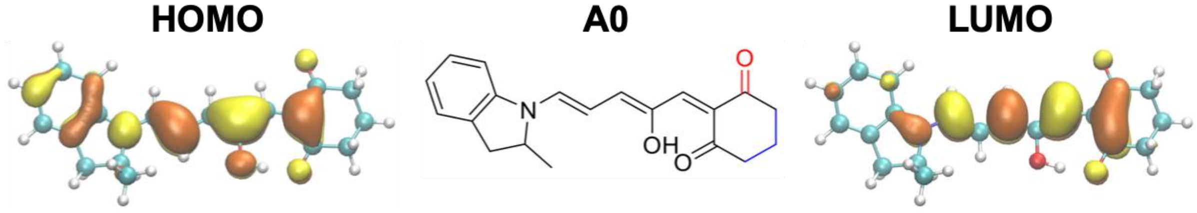

The system consists of a N containing donor group on the left, a system of conjugated double bonds in the middle and an acceptor ring on the right in which the blue group is variously substituted. The oxygen in red also forms part of the conjugated system of double bonds. This also means that both the HOMO and the LUMO will be affected to some degree if the oxygen is substituted, or if the blue part is changed, unlike in the typical usage of acceptors in which only the LUMO has contact with the acceptor. This means that the effect of substituents (**Rule 2**) is contrary to expectations in these systems and needs to be explicitly studied. Since we will focus on modifying the acceptor part, we will only show the right ring of the molecule in the subsequent discussion. To illustrate some of the rules mentioned above, we may consider the effect of modifications prescribed by them on the HOMO-LUMO gap, and on the STEOM excitation energy, which will be indicated in brackets in these figures:

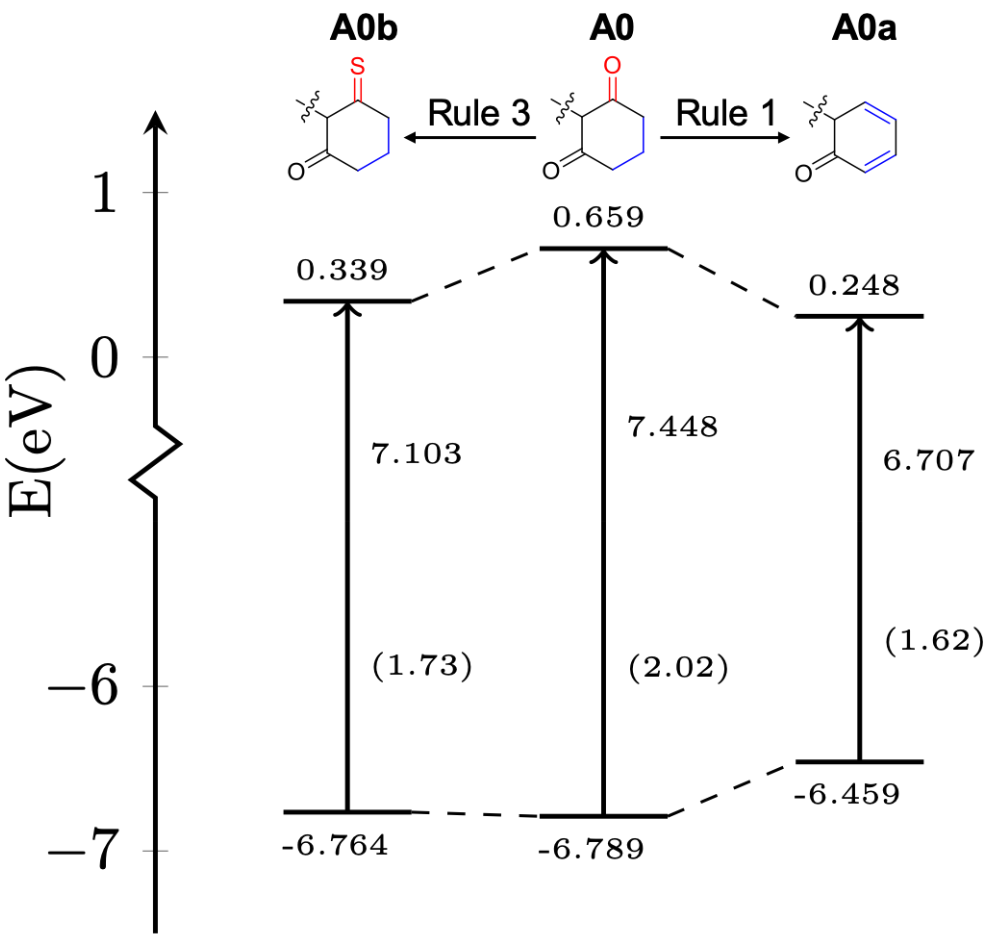

Applying **Rule 1** to the structure **A0** suggests a structure in which part of the acceptor ring is replaced by further conjugated double bonds, as in **A0a**. This yields the desired effect by stabilizing the LUMO and destabilizing the HOMO, however, it also makes the synthesis very difficult. Applying **Rule 3** seems more promising and can be achieved by substituting the oxygen marked in red by a sulfur atom (**A0b**). In this case, as it will frequently turn out to be with these systems, the HOMO is barely affected, but the LUMO is significantly stabilized by the substitution of O with S. Before attempting further applications of this rule, let us derive two commonly used acceptor groups from **A0**.

2.B. Two Common Acceptor Groups

Two common acceptors can also be derived from **A0** by applying **Rule 2** to the blue part of **A0**. It should be clear that these acceptors were not derived in this way in the literature. However, our goal is to apply design rules systematically and obtain insights that might help us come up with better alternatives to existing acceptor groups.

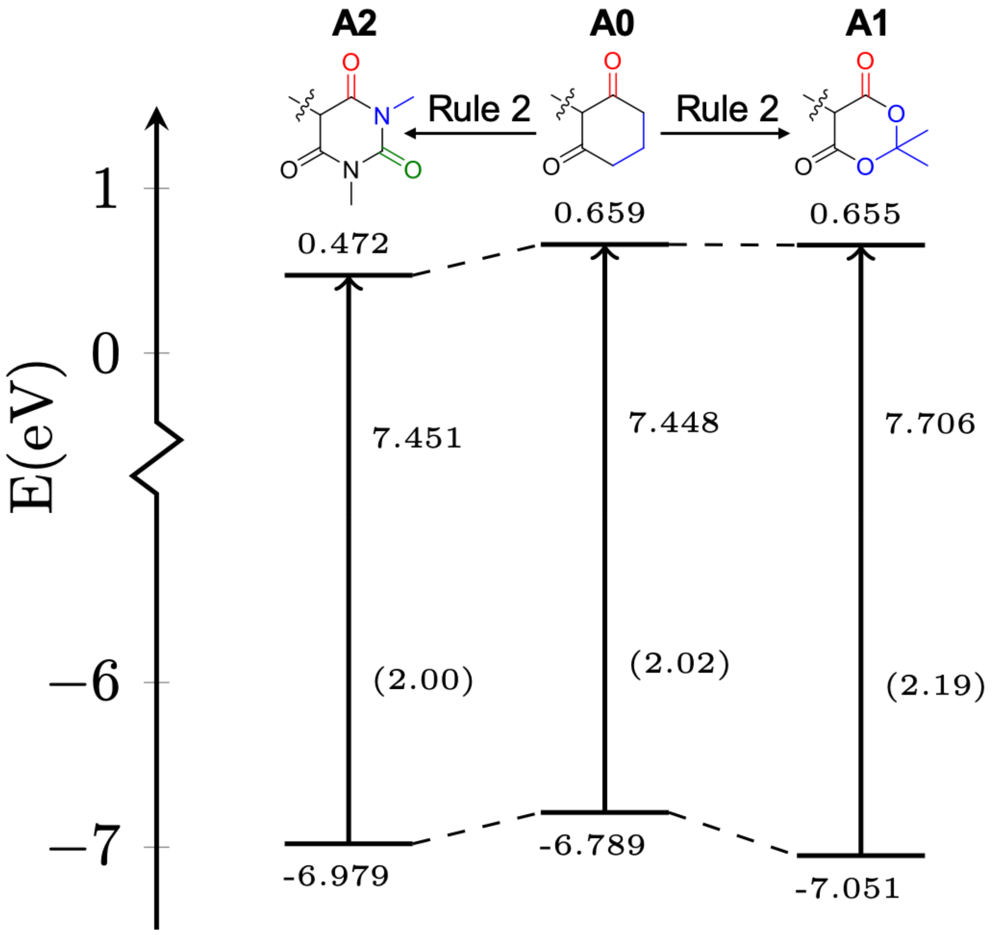

Substituting the part of **A0** highlighted in blue with electron withdrawing groups may yield many structures, two of which will be discussed here. Replacing the hydrocarbon chain with an oxygen containing group leads to structure **A1**, which is a non-planar ring structure. In the second case, the resulting **A2** structure contains two N atoms in a planar arrangement and a carbonyl group. The lone pairs of the N atoms and the π-bond of the carbonyl group are also delocalized over the participating atoms. Although both **A1** and **A2** are known and used structures in the context of donor-acceptor systems, viewing them as derivatives from **A0** highlights their deficiencies. In **A1**, only the HOMO is stabilized, while the LUMO is barely affected. Thus, **A1** is actually blue-shifted compared to **A0**. In **A2**, both the HOMO and the LUMO are stabilized to the same degree and as a result, there is barely any change in the excitation energies. **Rule 3** suggests that replacing the carbonyl oxygens in these groups leads to the closing of the HOMO-LUMO gap. We will now turn to investigate this for both **A1** and **A2**.

2.C. Modifying the A1 Acceptor Group

In **A1**, the oxygen marked in red is a continuation of the conjugated system of double bonds formed by the middle triene part of the molecule, hence **Rule 3** should apply. We will call the substituted structure **A1a**.

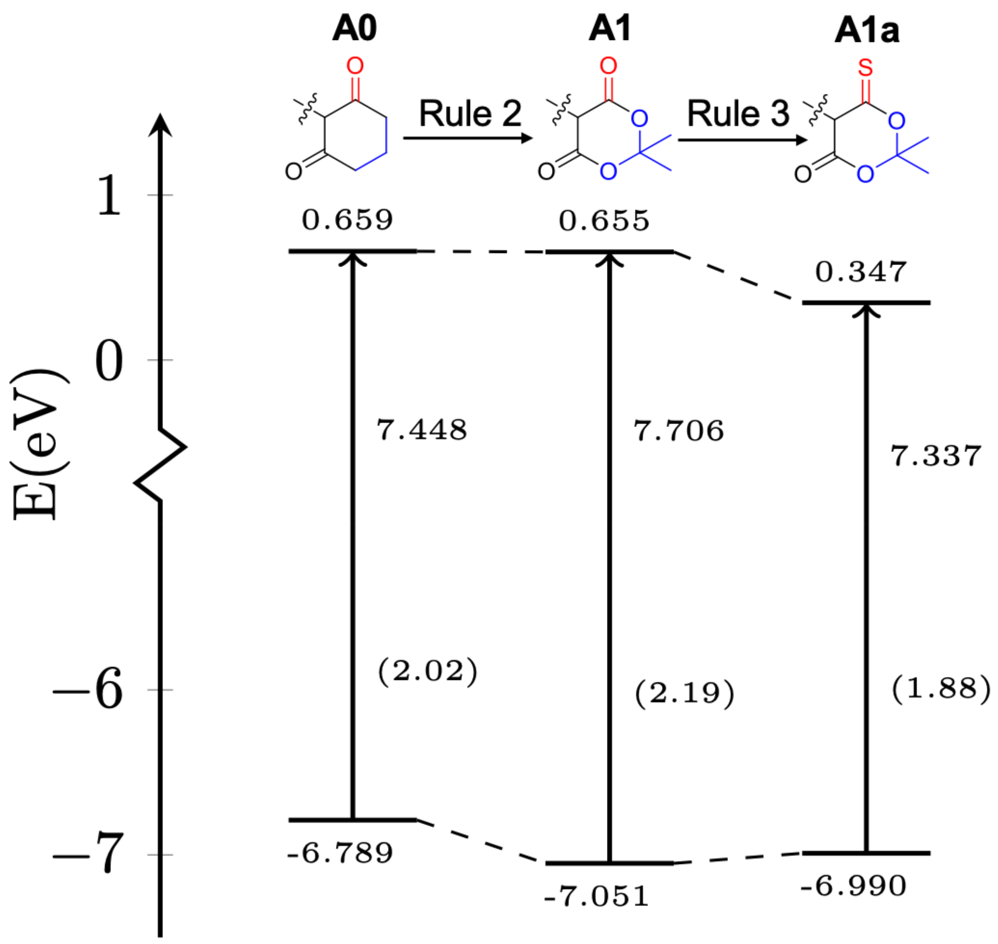

As expected, a substitution with sulfur closes the HOMO-LUMO gap. While the HOMO in **A1a** is only marginally stabilized with respect to **A1** (and still less stable than in **A0**), the red shift is due to the stabilization of the LUMO in **A1a** with respect to both **A1** and **A0**. However, placing a sulfur at that position is not the simplest option experimentally.

2.D. Modifying the A2 Acceptor Group

In **A2**, we may substitute the oxygen in red, or the oxygen that forms part of the substituent group, or both. We will denote the compound obtained from substituting the oxygen marked in red as **A2a**, the one obtained from substituting the O of the withdrawing group as **A2b**, while the structure in which both oxygens are replaced by sulfur will be referred to as **A2c**.

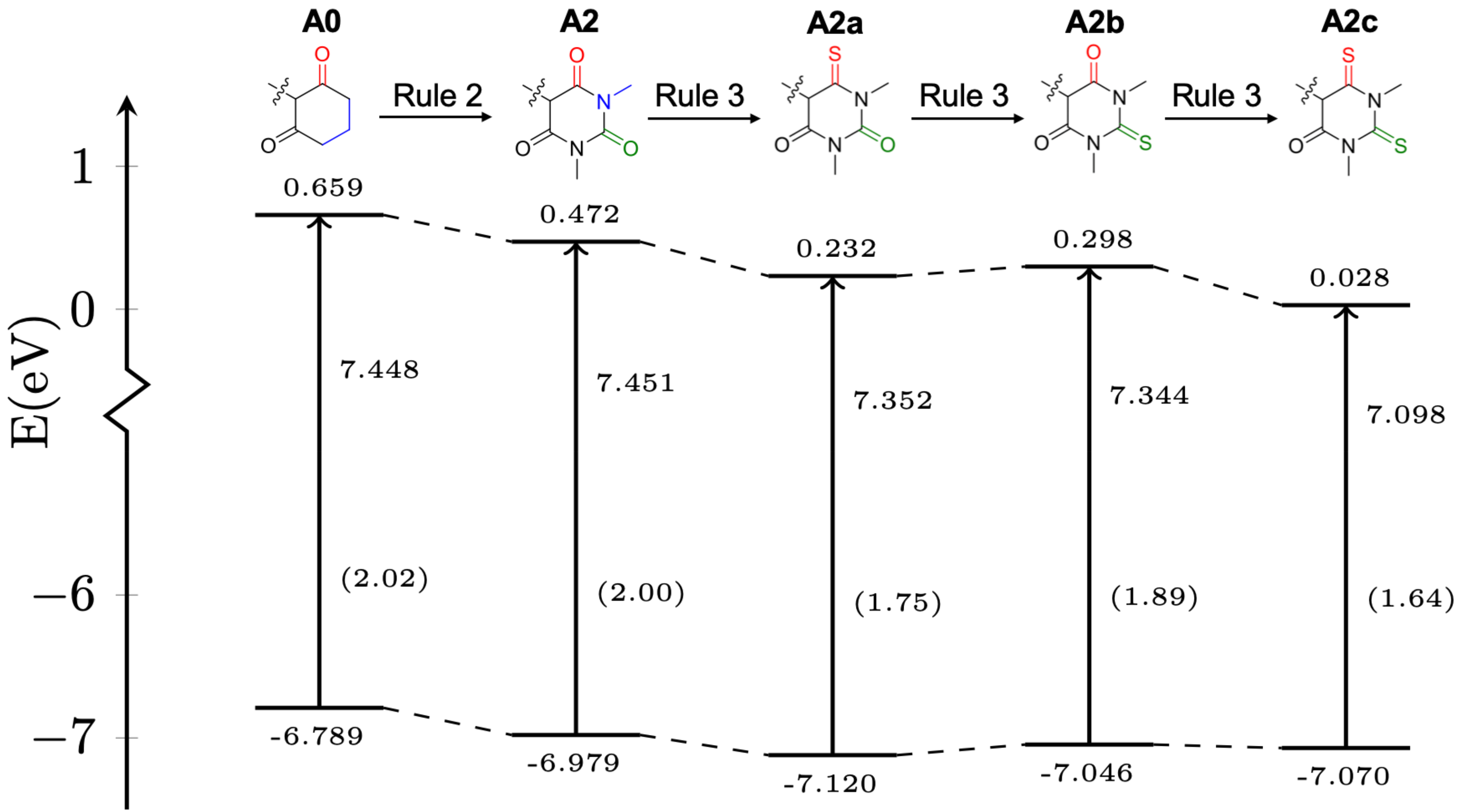

A0
A2
A2a
A2b
A2c
Rule 2
Rule 3
Rule 3
Rule 3
E(eV)
1
0
−6
−7
0.659
0.472
0.232
0.298
0.028
7.448
7.451
7.352
7.344
7.098
(2.02)
(2.00)
(1.75)
(1.89)
(1.64)
-6.789
-6.979
-7.120
-7.046
-7.070

In all the three compounds, **A2a**, **A2b** and **A2c**, both the HOMO and LUMO are stabilized compared to **A2** (and **A0**), but the LUMO is stabilized much more, leading to a red shift. In **A2a** and **A2b**, the shift is about the same magnitude, since the HOMO and the LUMO change together. In **A2c**, the HOMO does not change much compared to either **A2a** or **A2b**, while the LUMO is significantly stabilized compared to these two compounds, and especially to **A2**.

2.E. The Final Assessment

We have seen that applying **Rule 1** can be effective but can lead to compounds difficult to synthesize. **Rule 2** has essentially been exploited in the compounds studied in the literature (**A1** and **A2**). However, by studying the behavior of the frontier orbitals, it is possible to improve the red shift in these compounds further by substituting some of the carbonyl oxygens with sulfur. Thus, we are left with the compounds **A1a**, **A2a**, **A2b** and **A2c**. While the latter is the best candidate based on the predicted shift, it turns out that the simplest solution experimentally is to replace the oxygen of the substituent group in the **A2** compounds (the green oxygen). This leaves us with **A2b** as the best candidate. To ease the synthesis, we consider one final modification of this structure, by replacing the methyl groups on N with ethyl groups. The resulting structure **A2d** is commercially available and it behaves essentially the same way as (slightly better than) **A2b**, which is why we identify it as the most promising candidate both with respect to theoretical considerations and synthetic constraints.

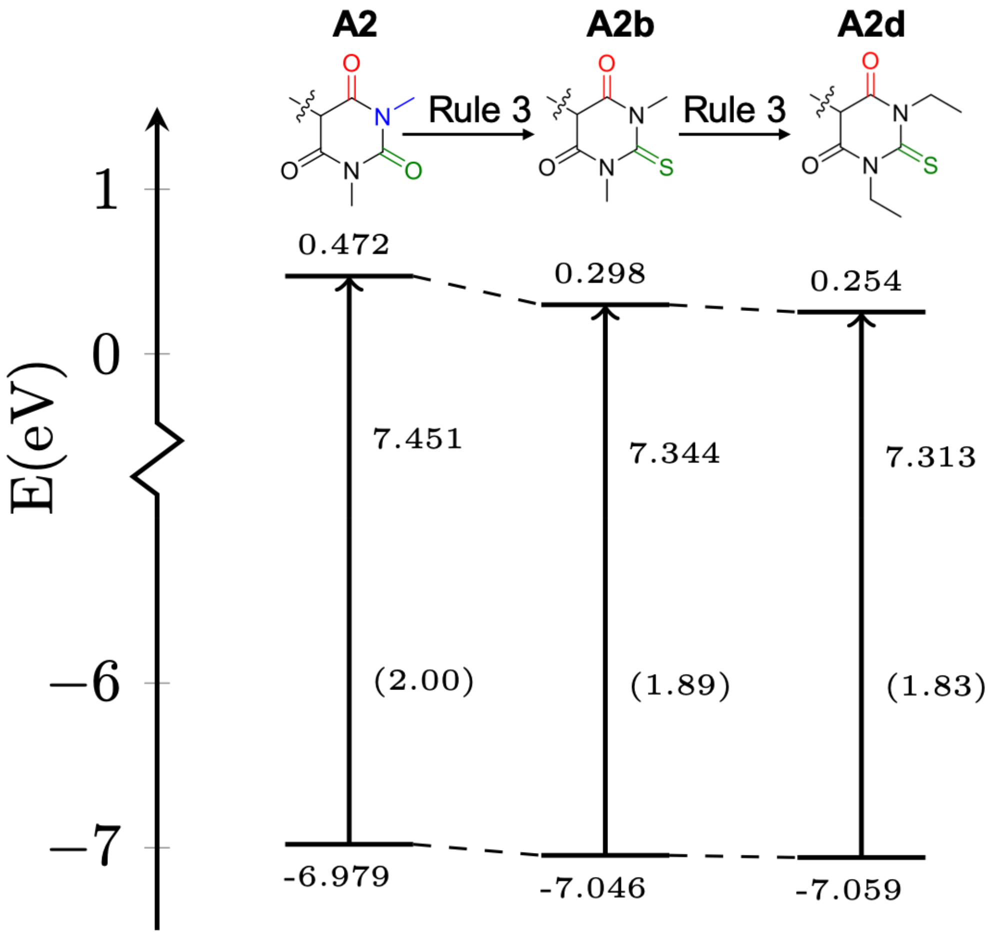

Molecular Orbitals and Difference Densities Plots

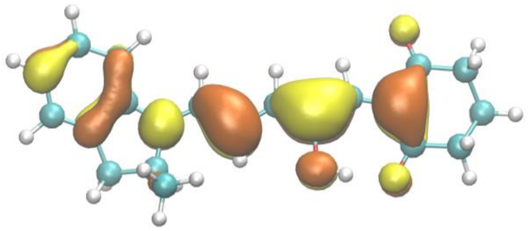

HOMO

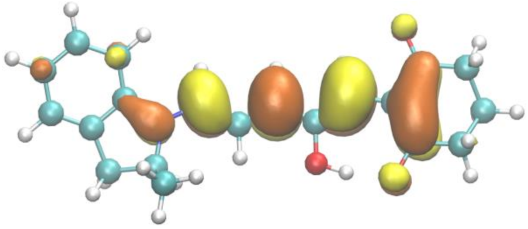

LUMO

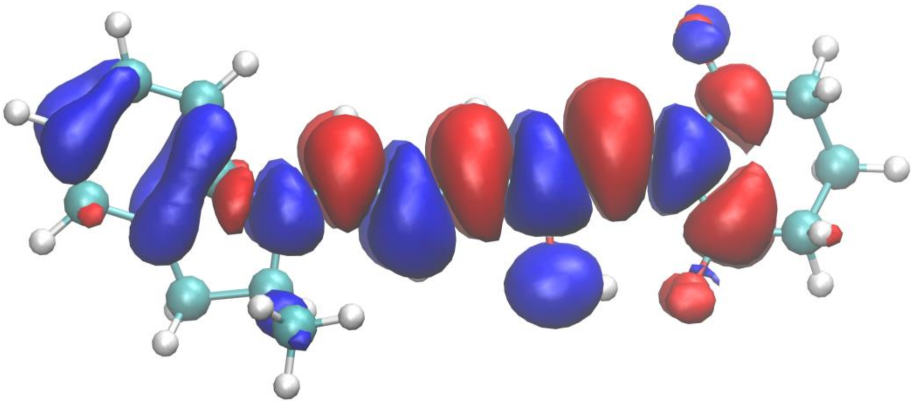

DDP

D1-A0

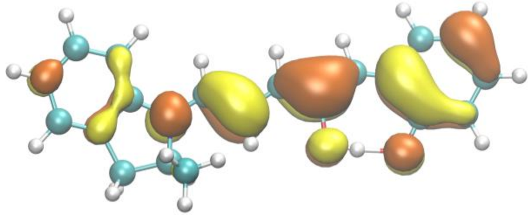

HOMO

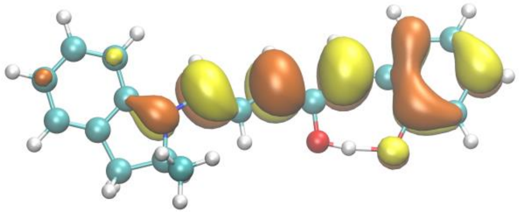

LUMO

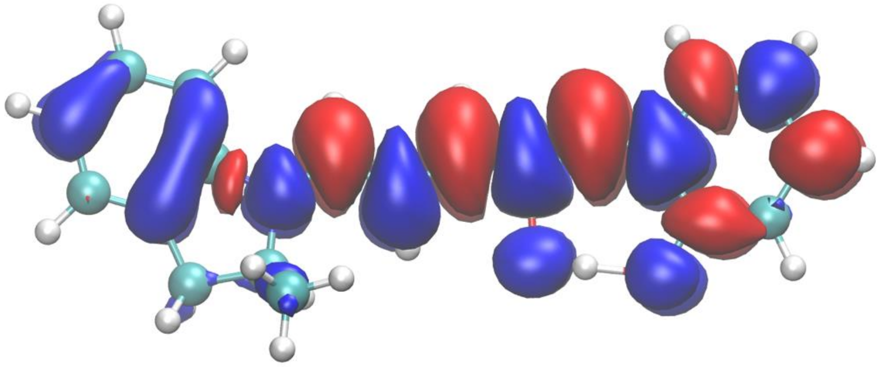

DDP

D1-A0a

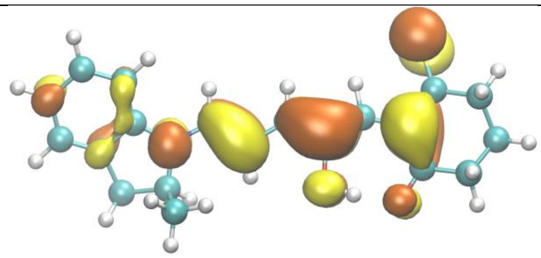

HOMO

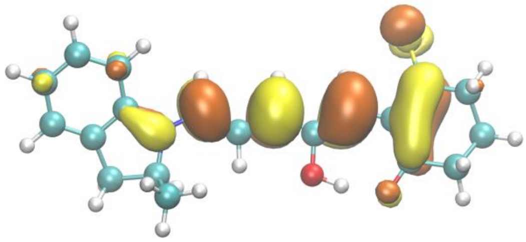

LUMO

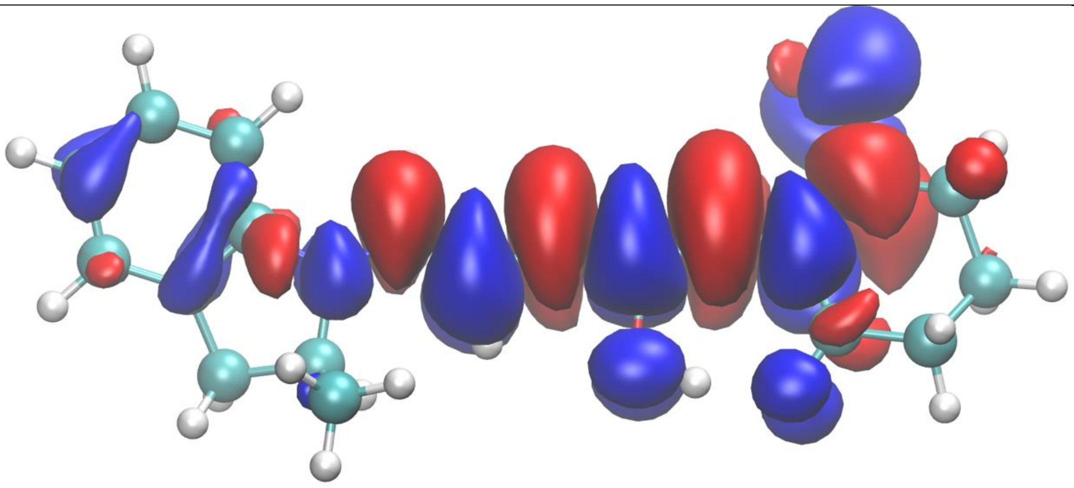

DDP

D1-A0b

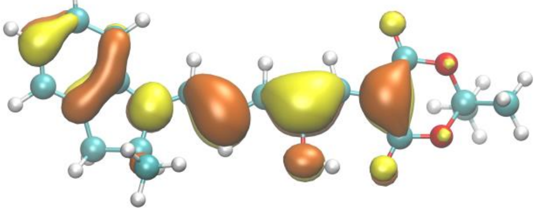

HOMO

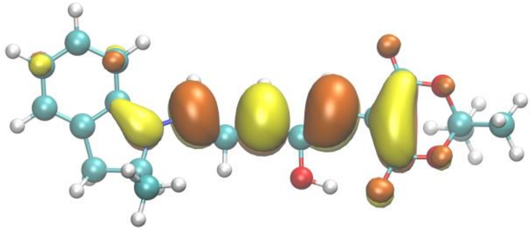

LUMO

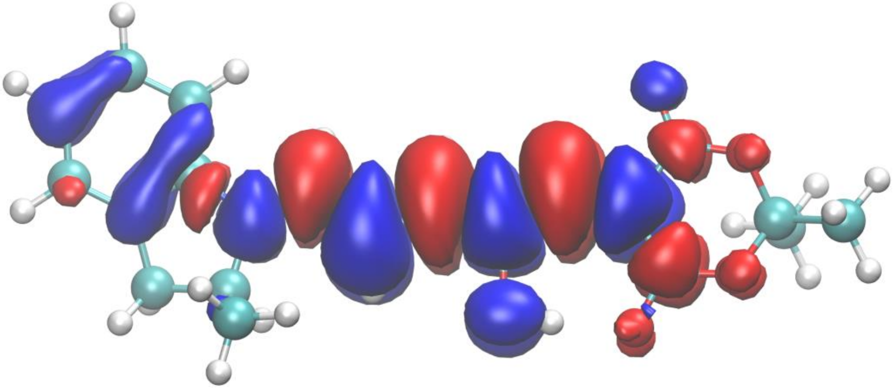

DDP

D1-A1

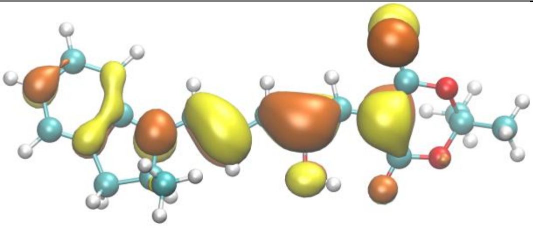

HOMO

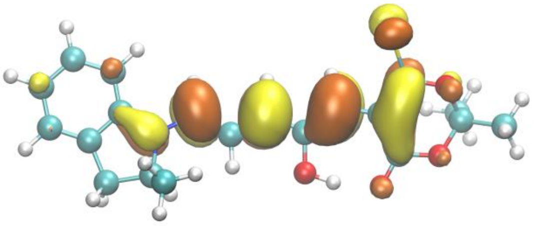

LUMO

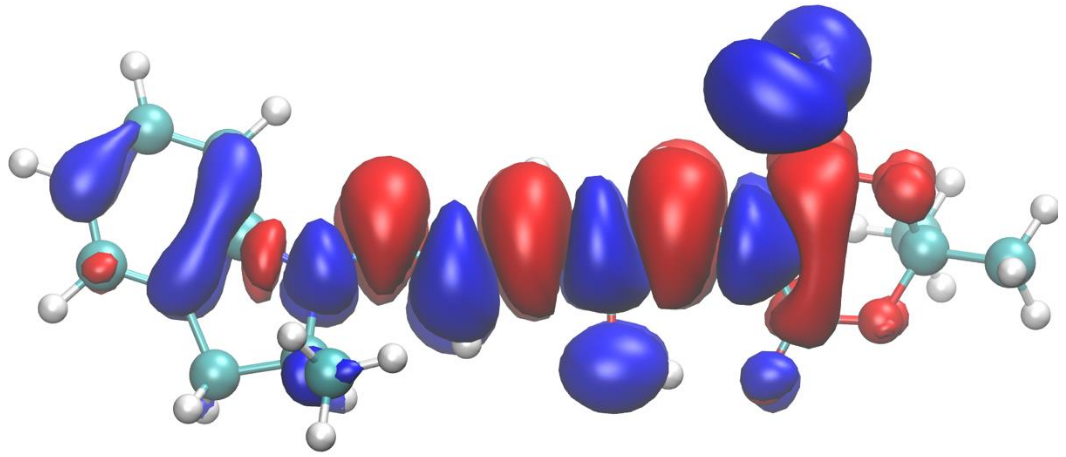

DDP

D1-A1a

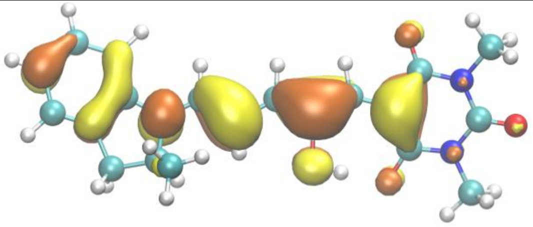

HOMO

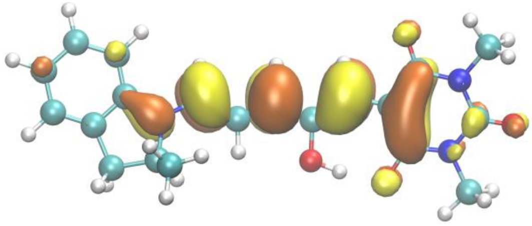

LUMO

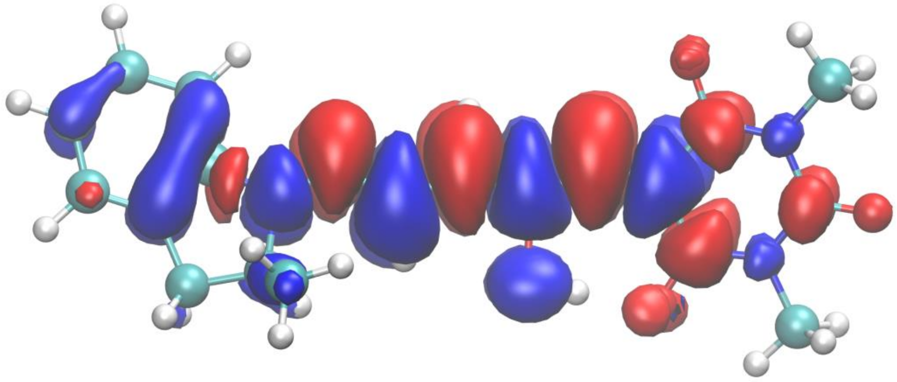

DDP

D1-A2

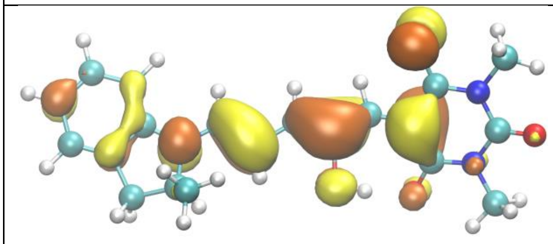

HOMO

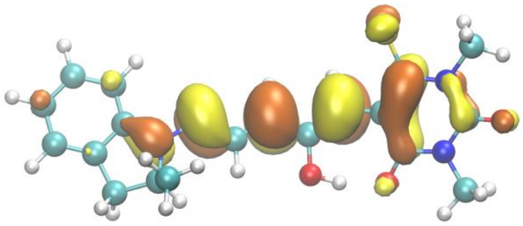

LUMO

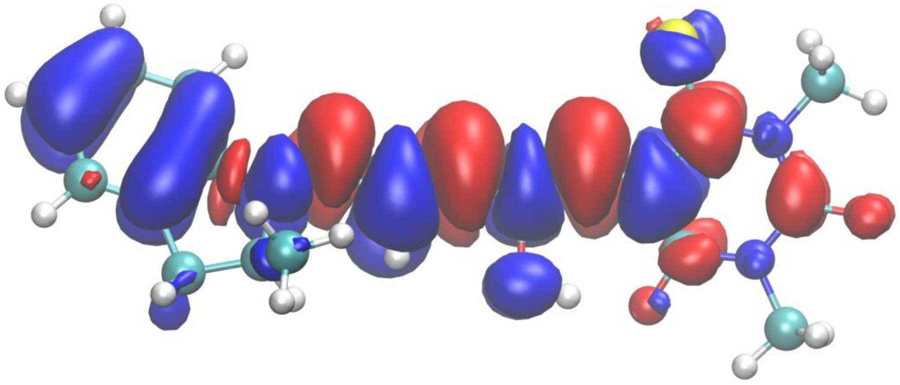

DDP

D1-A2a

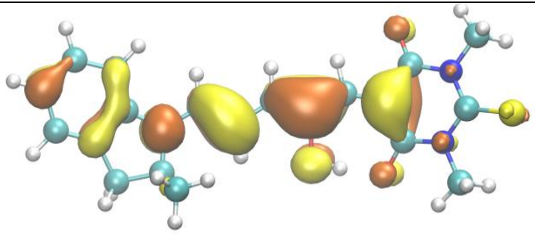

HOMO

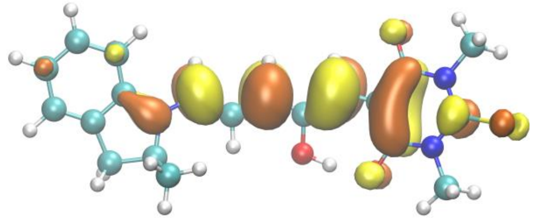

LUMO

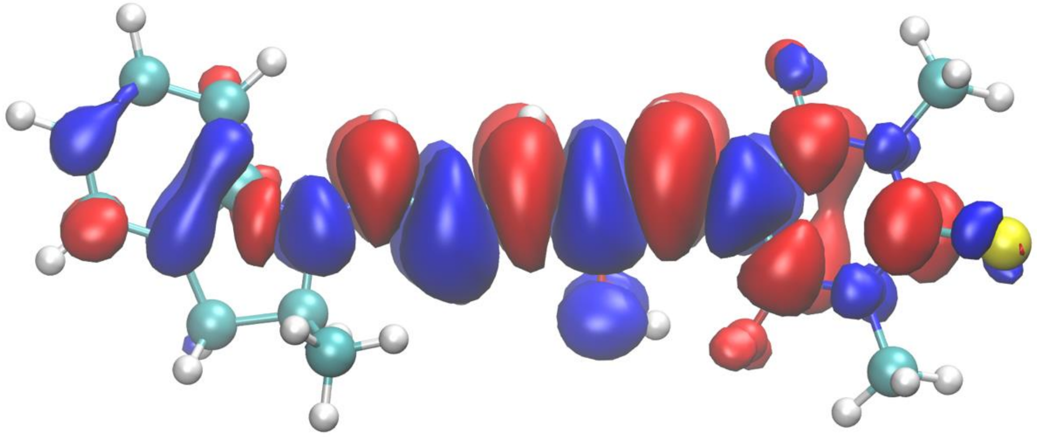

DDP

D1-A2b

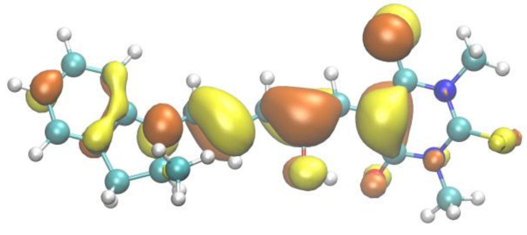

HOMO

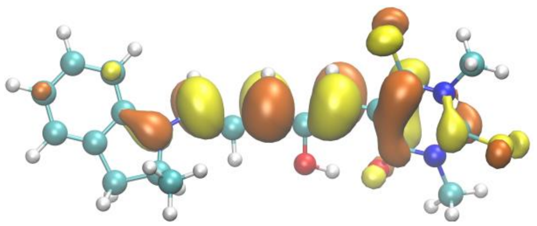

LUMO

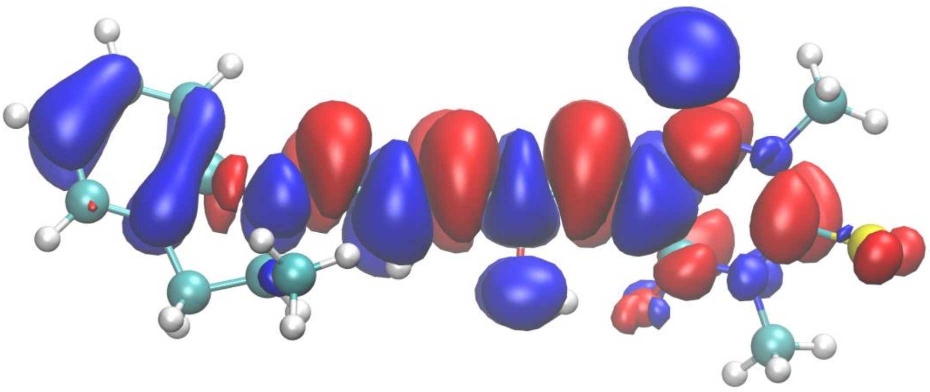

DDP

D1-A2c

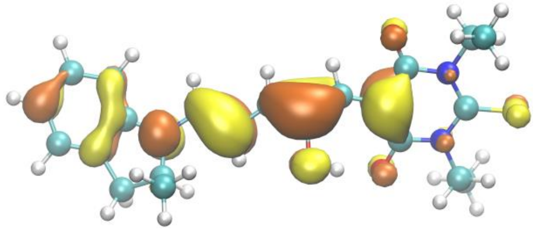

HOMO

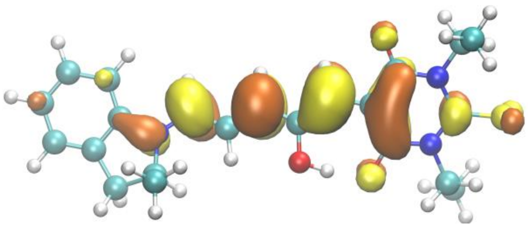

LUMO

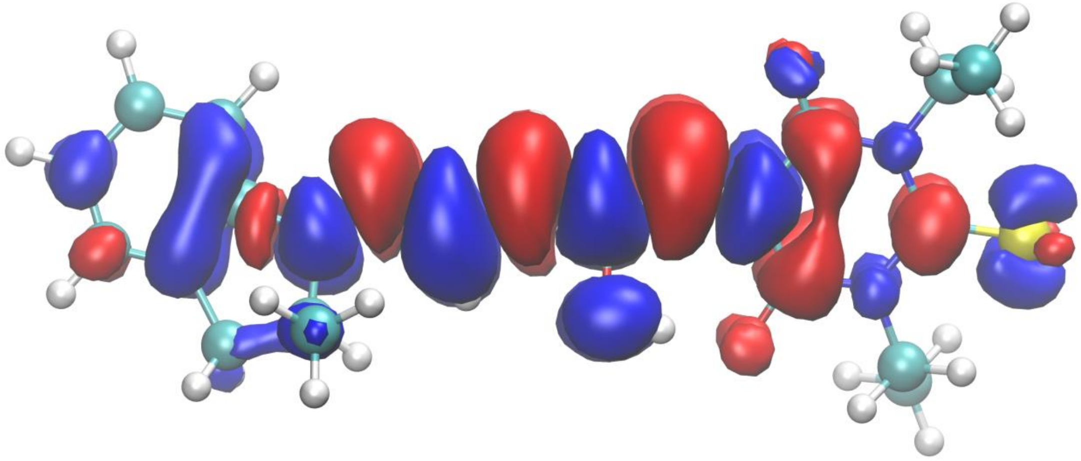

DDP

D1-A2d

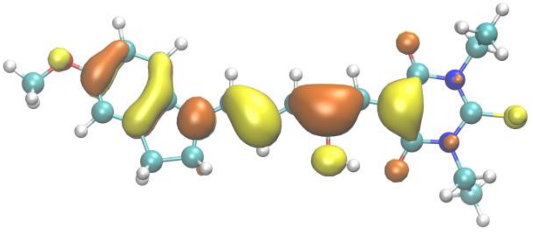

HOMO

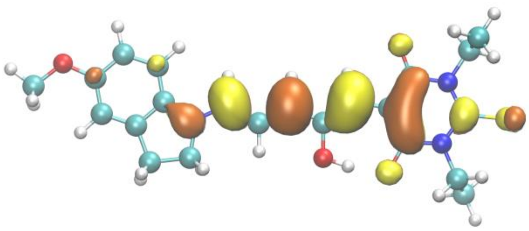

LUMO

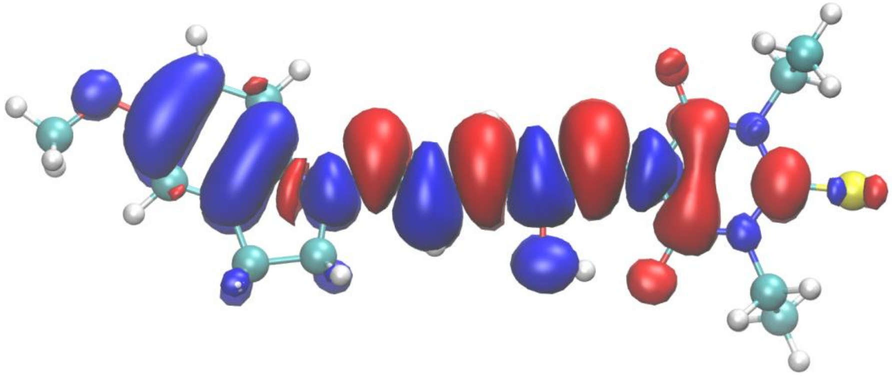

DDP

D2-A2d

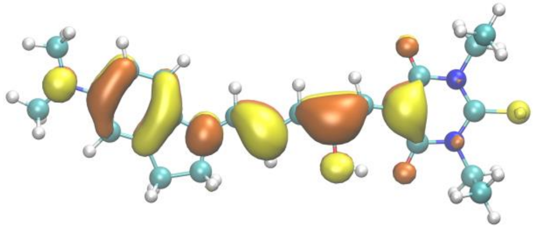

HOMO

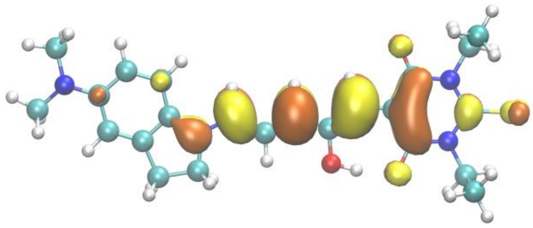

LUMO

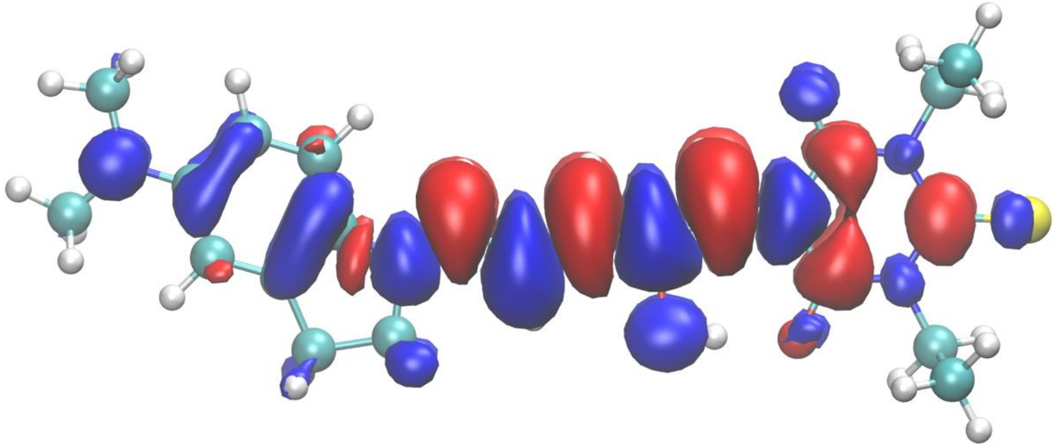

DDP

D3-A2d

Figure S4. Frontiers Orbitals and Density difference plots (DDP) for DASAs for the $S_0 \rightarrow S_1$ transition. The blue (red) zones indicate density decreases (increases) upon transition. The selected contour threshold for orbitals is 0.03 au and 0.0004 au for the DDP.

Experimental procedures

General information

All chemicals for synthesis were obtained from commercial sources and were used as received unless stated otherwise. Room temperature reactions were carried out at 22-25 °C. All chromatographic solvents were of ACS grade and were used without further purification. The starting material 1-(5-(diheptylamino)indolin-1-yl)ethan-1-one (**S6**) was prepared according to literature.[22]

Thin Layer Chromatography (TLC) was performed using silica gel 60 pre-coated aluminum plates (Macherey-Nagel 0.20 mm thickness) with a fluorescence indicator UV_{254}. For detection of components, UV light at 254 or 365 nm was used. Automated column chromatography was performed using an Interchim puriFlash XS520Plus purification system with silica gel (puriFlash 30 µm, Interchim) prepacked columns.

^1H and ^{13}C NMR spectroscopy were recorded on a Bruker AVANCE III HD spectrometer (300 MHz) at 25 °C. Coupling constants (*J*) are reported in Hz and chemical shifts values (δ) are reported in parts per million (ppm) with the solvent resonance as the internal standard ($CDCl_3$: δ 7.26 for ^1H, δ 77.16 for ^{13}C; CD_2Cl_2: δ 5.32 for ^1H, δ 53.83 for ^{13}C). The following abbreviations are used to indicate signal multiplicity: s (singlet), d (doublet), t (triplet), p (pentet), m (multiplet), dd (doublet of doublets) or ddd (doublet of doublet of doublets).

High resolution electrospray mass spectra (HRMS-ESI+) were recorded on a micrOTOF-Q spectrometer (Bruker). Accurate mass measurements were achieved by using sodium formate as an external reference.

General pump-probe absorption procedure

The pump beam was generated by light emitting diodes (LEDs) with an absolute irradiance of 27 mW/cm^2 (λ_{max} = 638) or 13 mW/cm^2 (λ_{max} = 731 nm). The probe beam was produced by Mini Deuterium Halogen Light Source (Ocean Optics DT-Mini-2-GS). Pump and probe beams were overlapped at a 90° angle inside a Peltier-based temperature-controlled cuvette holder. Sample solutions (5-10 µM) were measured after equilibrium was reached in the dark in a 10x10 mm quartz cuvette under magnetic stirring to ensure homogeneity. The probe beam was directed into the detector (OceanOptics USB4000-UV-Vis), which was connected to a PC via USB port.

Light emitting diodes for irradiation

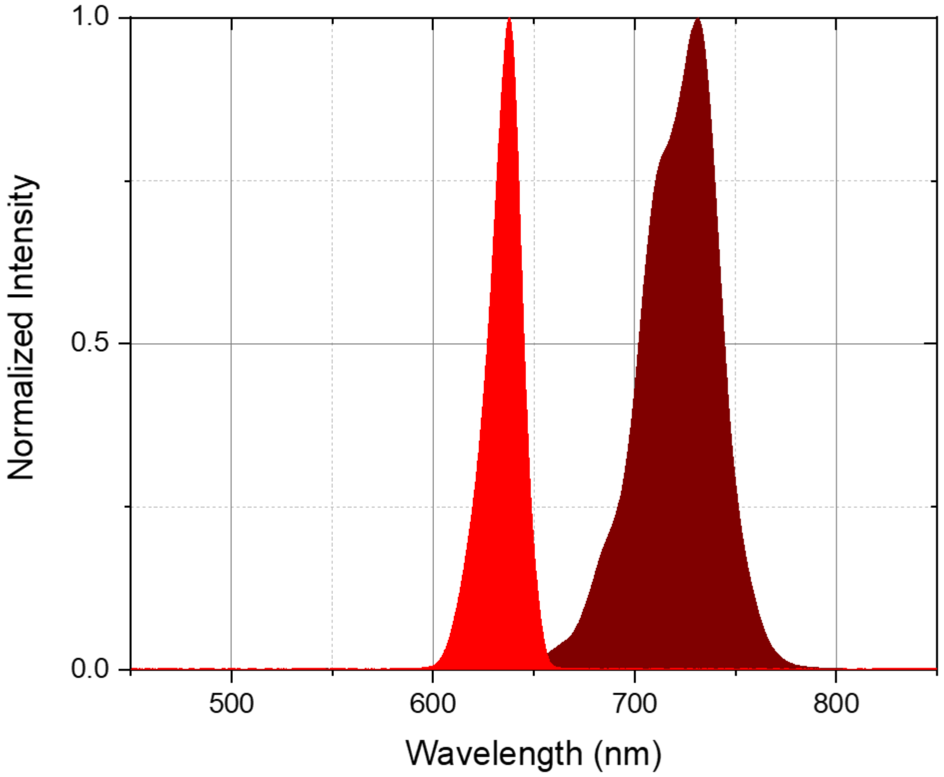

Figure S5. Emission profile for light emitting diodes used to irradiate the samples. Total irradiance (mW/cm^2): 27 (λ_{max} = 638 nm) and 13 (λ_{max} = 731 nm).

Synthetic procedure

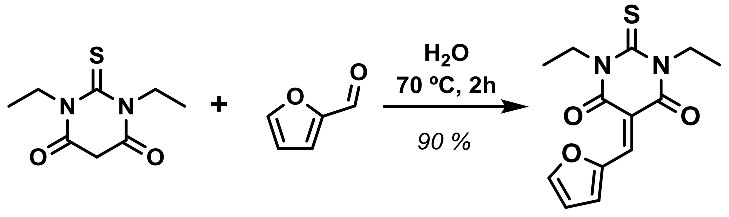

S1 **S2** **S3**

1,3-diethyl-5-(furan-2-ylmethylene)-2-thioxodihydropyrimidine-4,6(1*H*,5*H*)-dione (S3): 1,3-Diethyl-2-thiobarbituric acid **S1** (500 mg, 2.50 mmol) and furfural **S2** (207 µL, 2.50 mmol) were added sequentially to 10 mL H_2O. The heterogeneous mixture was stirred at 70 °C for 2 hours. At the completion of the reaction (monitored by TLC), the formed yellow precipitate was collected by vacuum filtration and washed twice with cold H_2O. The resultant solid was dissolved in dichloromethane (75 mL) and washed sequentially with 30 mL sat. aq. $NaHSO_3$, 30 mL H_2O, 30 mL sat. aq. $NaHCO_3$ and 30 mL brine. After drying with $MgSO_4$, the solids were removed by filtration and the solution was concentrated under reduced pressure to give 625 mg (90 %) of compound **S3** as a bright yellow powder. ^{1}**H NMR** (300 MHz, $CDCl_3$) δ 8.71 (d, *J*= 3.9 Hz, 1H), 8.44 (s, 1H), 7.89 (dd, *J*= 1.6, 0.6 Hz, 1H), 6.76 (ddd, *J*= 3.9, 1.6, 0.8 Hz, 1H), 4.62-4.53 (m, 4H), 1.35-1.28 (m, 6H). ^{13}**C NMR** (75 MHz, $CDCl_3$) δ 178.9, 160.9, 158.9, 151.8, 151.1, 142.0, 128.9, 115.6, 112.4, 44.2, 43.5, 12.6, 12.5. **HRMS** (ESI+) *m/z* calc. for C13H15N2O3S $[M+H]^+$: 279.0798, found: 279.0792.

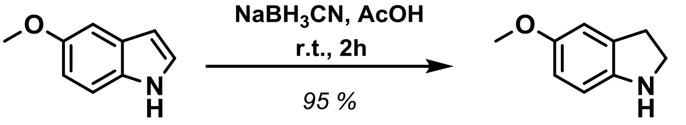

S4 **S5**

5-methoxyindoline (S5): 5-methoxyindole **S4** (200 mg, 1.36 mmol) was dissolved in glacial acetic acid (12 mL). Then, sodium cyanoborohydride (257 mg. 4.08 mmol) was added in small portions to the stirring solution at room temperature. The reaction mixture was stirred for 2 h and monitored by TLC. Upon completion of the reaction, water (1 mL) was added to the reaction mixture and all volatiles were evaporated. After that, aq. NaOH solution was added to the residue adjusting to pH > 9 and the aqueous phase was extracted with dichloromethane. The combined organic extracts were washed with water and sat. aq. $NaHCO_3$ solution, then dried over $MgSO_4$, filtered and concentrated under reduced pressure to afford 5-methoxyindoline (**S5**, 193 mg, 95 % yield) as a yellow oil. Spectral properties matched previously reported values.[24] ^{1}**H NMR** (300 MHz, $CDCl_3$) *δ* 6.76 (s, 1H), 6.61–6.58 (m, 2H), 3.75 (s, 3H), 3.53 (t, *J* = 8.3 Hz, 2H), 3.44 (s, 1H), 3.01 (t, *J* = 8.3 Hz, 2H). ^{13}**C NMR** (75 MHz, $CDCl_3$) *δ* 153.6, 145.4, 131.3, 112.2, 111.7, 110.2, 56.1, 47.9, 30.6. **HRMS** (ESI+) *m/z* calc. for C9H12NO $[M+H]^+$: 150.0913, found: 150.0917.

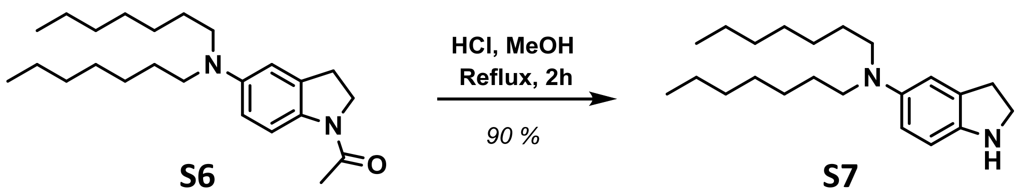

S6 **S7**

***N,N*-diheptylindolin-5-amine (S7):** 100 mg of **S6** were placed in a flask with 2.5 mL HCl (6 M) and 2.5 mL MeOH. The mixture was then heated to reflux for two hours. After cooling to room temperature, the reaction was cautiously basified with NaOH (2.5 M) and extracted with dichloromethane. The crude product was purified by flash column chromatography on silica gel using 15-25 % ethyl acetate in hexane as eluent to give the product (**S7**, 90 mg, 90 % yield) as an oil. Spectral properties matched previously reported values.[22] **^{1}H NMR** (300 MHz, $CDCl_3$) δ 6.64 (s, 1H), 6.61 (d, *J*= 8.4 Hz, 1H), 6.47 (d, *J*= 8.4 Hz, 1H), 3.51 (t, *J*= 8.1 Hz, 2H), 3.11 (t, *J*= 8.1 Hz, 4H), 3.00 (t, *J*= 8.1 Hz, 2H), 1.51 (p, *J*= 7.6 Hz, 4H), 1.34-1.25 (m, 16H), 0.92-0.85 (m, 6H). **^{13}C NMR** (75 MHz, $CDCl_3$) δ 143.3, 142.9, 131.2 113.8, 112.4, 110.9, 52.8, 47.8, 32.1, 31.0, 29.4, 27.5, 27.4, 22.8, 14.2. **HRMS** (ESI+) *m/z* calc. for C22H39N2 $[M+H]^+$: 331.3108, found: 331.3107.

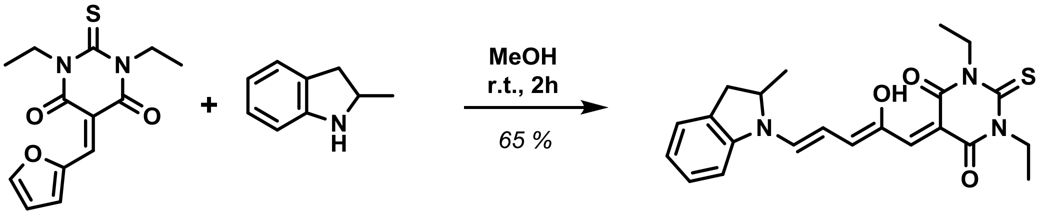

S3 **S8** **D1-A2d**

1,3-diethyl-5-((2*Z*,4*E*)-2-hydroxy-5-(2-methylindolin-1-yl)penta-2,4-dien-1-ylidene)-2-thioxodihydropyrimidine-4,6(1*H*,5*H*)-dione (D1-A2d): The furan adduct **S3** (75 mg, 0.27 mmol) was suspended in 3 mL methanol. To this suspension 2-methylindoline (**S8**, 44 µL, 0.34 mmol) was added and the mixture was stirred at room temperature. Upon completion of the reaction as assessed by TLC, the reaction mixture was cooled down to room temperature and then further to -20 °C for 30 min. The formed blue precipitate was filtered off and washed thoroughly with cold diethyl ether and cold hexane to yield dark blue crystals (**D1-A2d**, 72 mg, 65 % yield). **^{1}H NMR** (300 MHz, CD_2Cl_2) δ 12.61 (s, 1H), 7.79 (d, *J*= 12.7 Hz, 1H), 7.36-7.29 (m, 2H), 7.22-7.15 (m, 3H), 6.93 (d, *J*= 12.4 Hz, 1H), 6.42 (t, *J*= 12.2 Hz, 1H), 4.80-4.70 (m, 1H), 4.59-4.48 (m, 4H), 3.54 (dd, *J*= 16.3, 8.7 Hz, 1H), 2.86 (d, *J*= 16.4 Hz, 1H), 1.44 (d, *J*= 6.5 Hz, 3H), 1.30-1.22 (m, 6H). **^{13}C NMR** (75 MHz, CD_2Cl_2) δ 177.7, 163.3, 161.3, 149.9, 149.3, 145.2, 141.2, 140.3, 132.6, 128.8, 126.9, 126.8, 111.3, 106.2, 102.5, 58.4, 43.9, 43.8, 36.6, 19.9, 12.6, 12.4. **HRMS** (ESI+) *m/z* calc. for C22H26N3O3S $[M+H]^+$: 412.1689, found: 412.1685.

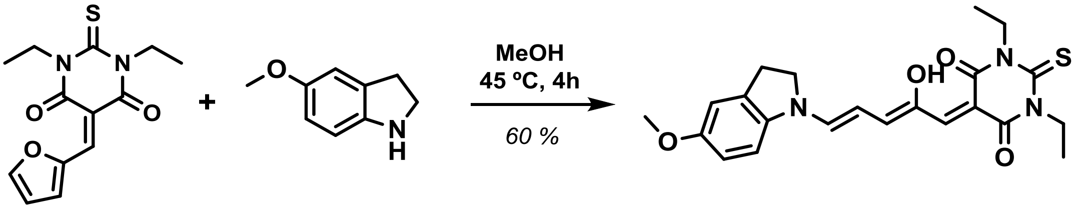

S3 **S5** **D2-A2d**

1,3-diethyl-5-((2*Z*,4*E*)-2-hydroxy-5-(5-methoxyindolin-1-yl)penta-2,4-dien-1-ylidene)-2-thioxodihydropyrimidine-4,6(1*H*,5*H*)-dione (D2-A2d): 5-Methoxyindoline (**S5**, 100 mg, 0.67 mmol) was added to a stirring flask containing the furan adduct **S3** (75 mg, 0.27 mmol) in 5 mL MeOH. The mixture was stirred at 45 °C until the reaction was completed (monitored by TLC) and the product was cooled down to room temperature and then further to -20 °C for 30 min. The green precipitate was filtered off and washed thoroughly with cold diethyl ether and cold hexane to yield green crystals (**3**, 70 mg, 60 % yield). **^{1}H NMR** (300 MHz, CD_2Cl_2) δ 12.67 (s, 1H), 7.83 (d, *J*= 12.1 Hz, 1H), 7.15 (d, *J*= 8.7 Hz, 1H), 7.09 (s, 1H), 6.94 (d, *J*= 12.6 Hz, 1H), 6.89-6.84 (m, 2H), 6.31 (t, *J*= 12.3 Hz, 1H), 4.59-4.48 (m, 4H), 4.22 (t, *J*= 7.7 Hz, 1H), 3.82 (s, 3H), 3.33 (t, *J*= 7.8 Hz, 2H), 1.29-1.22 (m, 6H). **^{13}C NMR** (75 MHz, CD_2Cl_2) δ 177.5, 163.3, 161.4, 159.6, 149.9, 149.1, 145.1, 145.1, 138.0, 135.8, 114.7, 111.9, 111.8, 106.8, 101.6, 56.2, 50.5, 43.9, 43.7, 28.0, 12.7, 12.5. **HRMS** (ESI+) *m/z* calc. for C22H26N3O4S $[M+H]^+$: 428.1639, found: 428.1632.

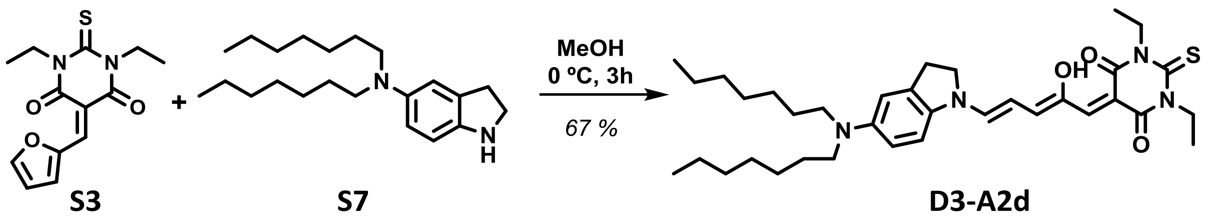

S3 **S7** **D3-A2d**

5-((2*E*,4*E*)-5-(5-(diheptylamino)indolin-1-yl)penta-2,4-dien-1-ylidene)-1,3-diethyl-2-thioxodihydropyrimidine-4,6(1*H*,5*H*)-dione (D3-A2d): *N*,*N*-diheptylindolin-5-amine (**S7**, 25 mg, 0.076 mmol) was added to a stirring flask containing the furan adduct **S3** (21 mg, 0.076 mmol) in 2 mL MeOH at 0 °C. After monitoring the reaction by TLC, the formed blue precipitate was filtered and washed thoroughly with cold diethyl ether and cold hexane to give 31 mg of dark blue crystals (**D3-A2d**, 67 % yield). **^{1}H NMR** (300 MHz, CD_2Cl_2) δ 12.76 (s, 1H), 7.81 (d, *J*= 11.9 Hz, 1H), 7.11 (d, *J*= 9.0 Hz, 1H), 6.91 (d, *J*= 12.9 Hz, 1H), 6.79 (s, 1H), 6.56 (d, *J*= 9.1 Hz, 1H), 6.51 (s, 1H), 6.35 (t, *J*= 12.4 Hz, 1H), 4.54-4.44 (m, 4H), 4.22 (t, *J*= 7.2 Hz, 2H), 3.33-3.23 (m, 6H), 1.63-1.53 (m, 4H), 1.36-1.27 (m, 16H), 1.27-1.20 (m, 6H), 0.93-0.87 (m, 6H). **^{13}C NMR** (75 MHz, CD_2Cl_2) δ 176.8, 162.9, 161.5, 149.9, 149.1, 148.5, 144.2, 136.7, 131.9, 129.9, 112.9, 112.2, 108.2, 108.0, 99.4, 51.8, 51.1, 43.7, 43.6, 32.3, 29.6, 28.1, 27.6, 27.4, 23.0, 14.3, 12.7, 12.5. **HRMS** (ESI+) *m/z* calc. for C35H53N4O3S $[M+H]^+$: 609.3833, found: 609.3814.

Characterization data

Comparison of absorption spectra of DASAs D1-A2 and D1-A2d

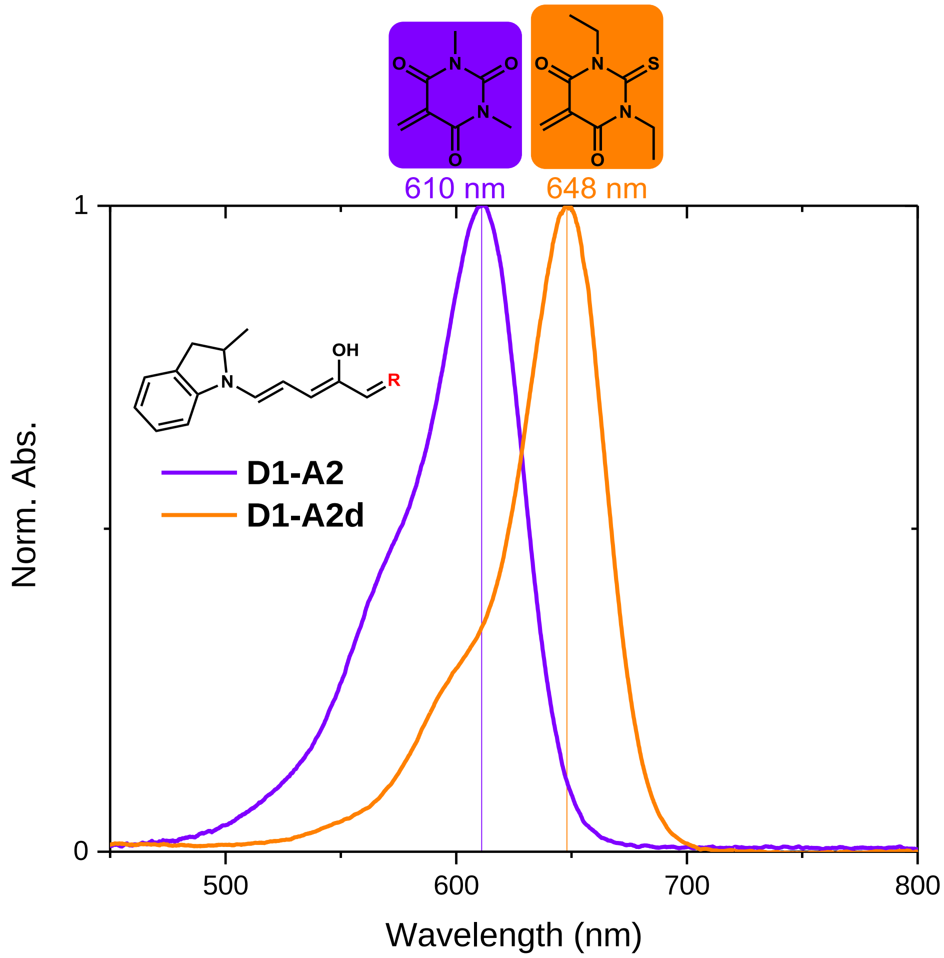

Figure S6. Comparison of the absorption spectra of the newly designed thiobarbituric acid-based DASA **D1-A2d** and its barbituric acid-based analogue **D1-A2** in toluene. Absorption maxima provided under the corresponding acceptor group.

Comparison of absorption spectra of DASAs D1-A2d, D2-A2d and D3-A2d

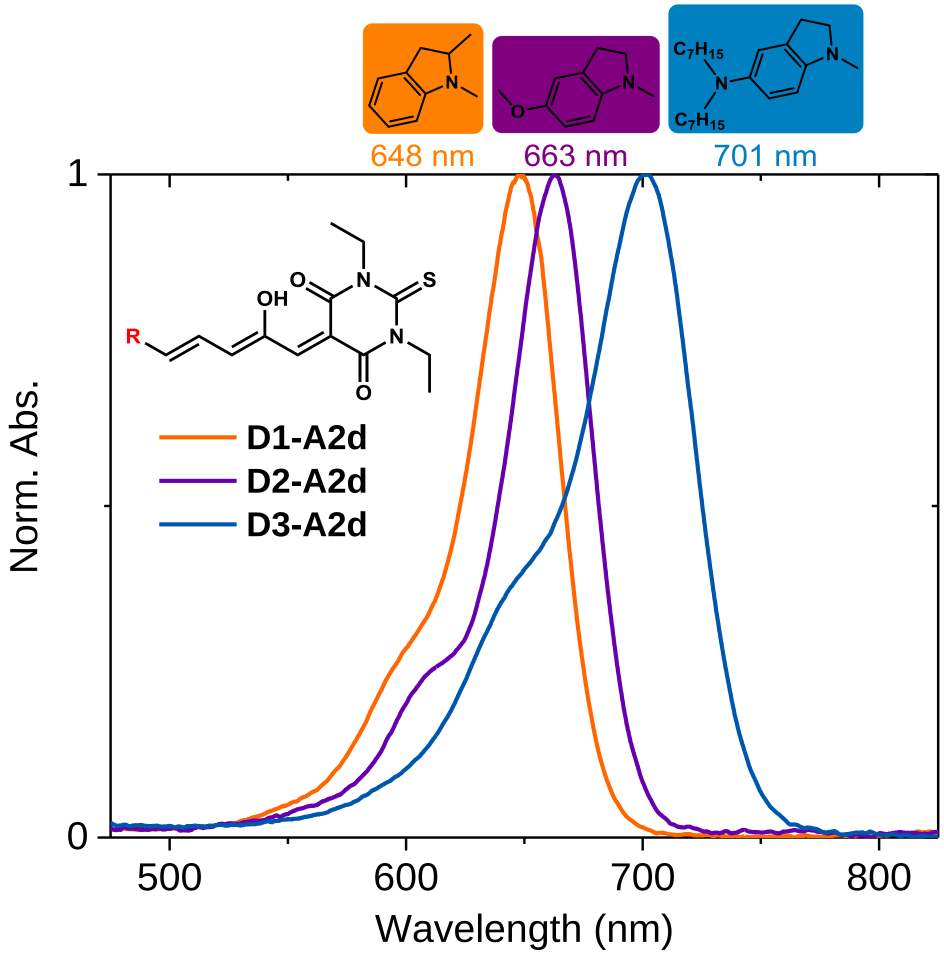

Figure S7. Comparison of the absorption spectra of DASAs with the newly designed thiobarbituric acid acceptor and different indolenine donor groups (in toluene). Absorption maxima provided under the corresponding donor moiety.

Solvatochromic effect

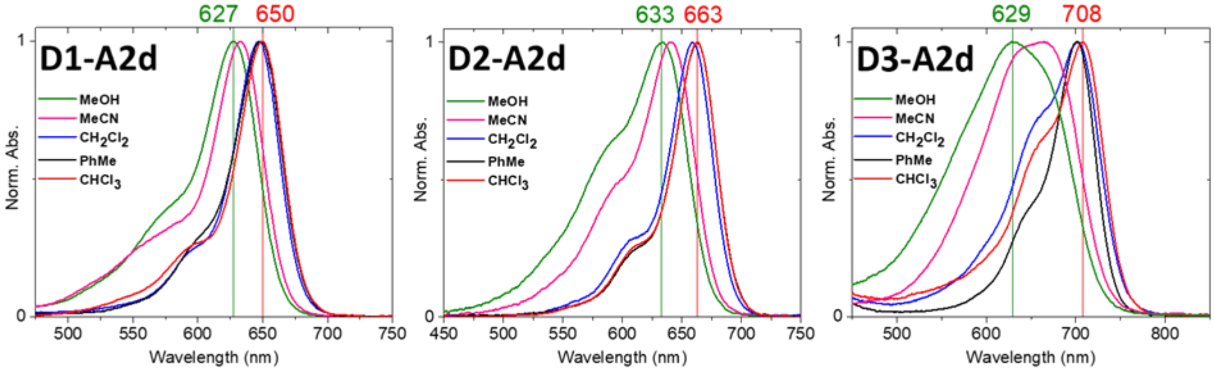

Figure S8. Comparison of absorption spectra of **D1-A2d**, **D2-A2d** and **D3-A2d** in different solvents.

Molar absorptivity

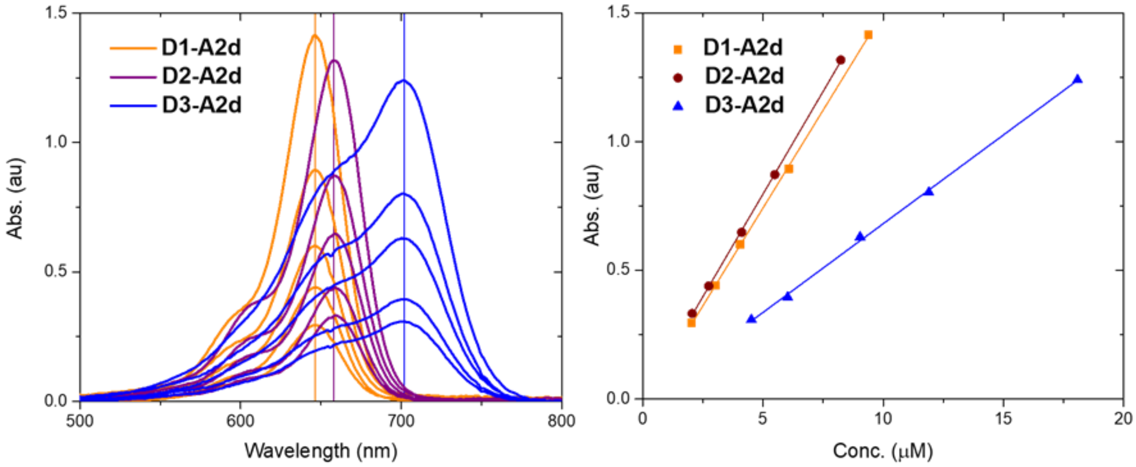

DASA	λ (nm)	ε (M^{-1} cm^{-1})
**D1-A2d**	647	152000 ± 1000
**D2-A2d**	659	160000 ± 1000
**D3-A2d**	702	69000 ± 1000

Figure S9. Molar extinction coefficients (*ε*) of DASAs **D1-A2d**, **D2-A2d** and **D3-A2d** in dichloromethane.

Pump-probe absorption experiments of DASA D1-A2d

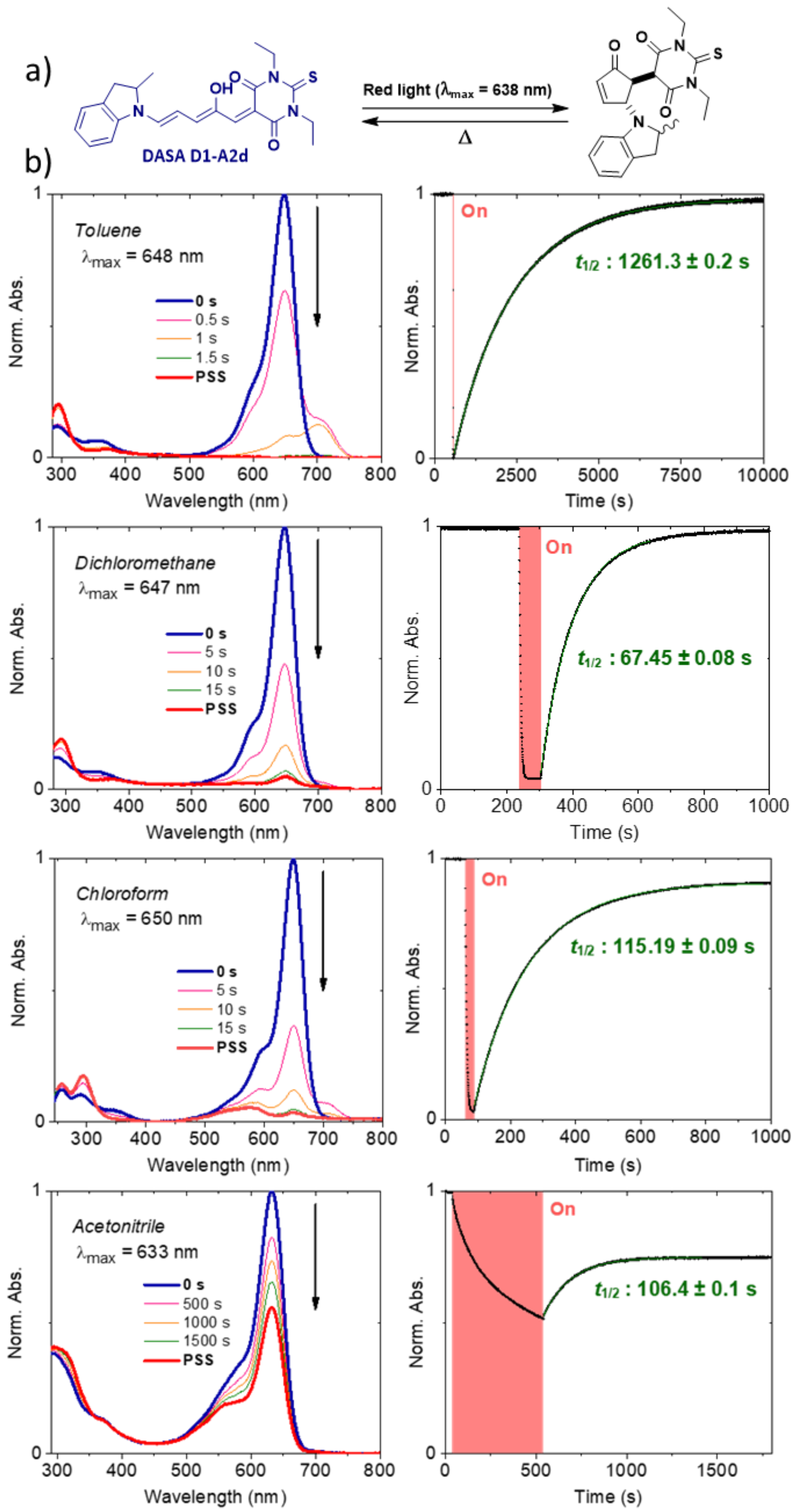

Figure S10. Photochromism of DASA **D1-A2d** in different solvents irradiating with red light (λ_{max} = 638 nm). (a) Chemical structures of both isomers. (b) Photochromic behavior at 20 °C showing full absorption profile over time until the photostationary state is reached (left) and peak absorption over time (right). Periods of red light irradiation are represented by red blocks.

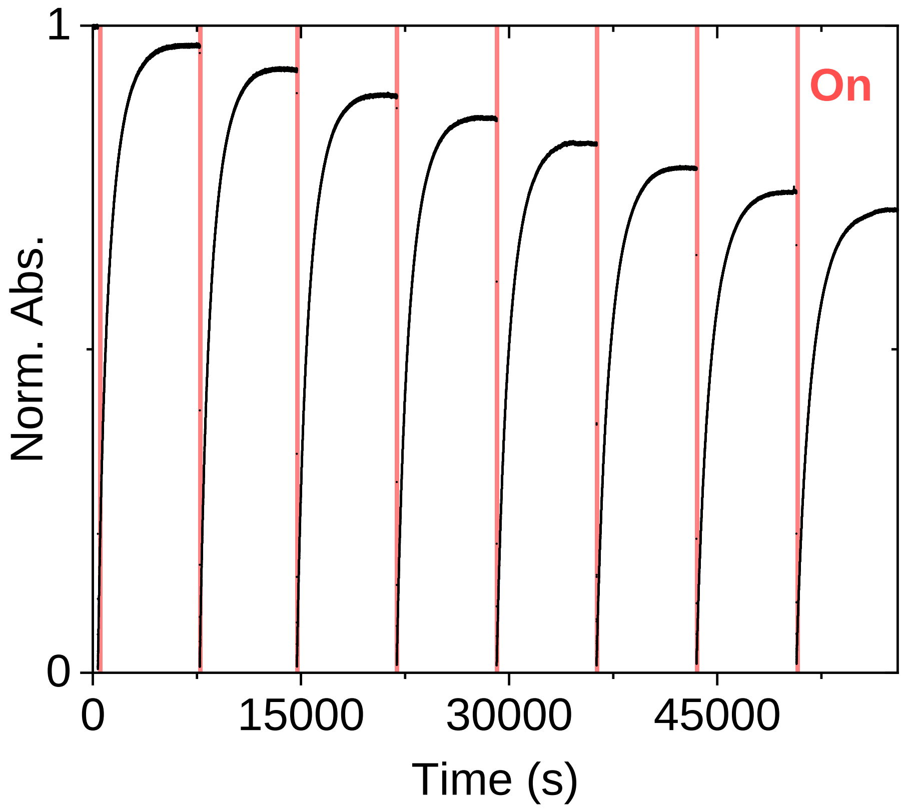

Figure S11. Photochromic cycling of DASA **D1-A2d** in toluene (20 °C). The absorption was measured at the absorption maximum (648 nm) and the red blocks indicate periods of irradiation (λ_{max} = 638 nm). Approximately 3-5 % permanent bleaching is observed per cycle.

Pump-probe absorption experiments of DASA D2-A2d

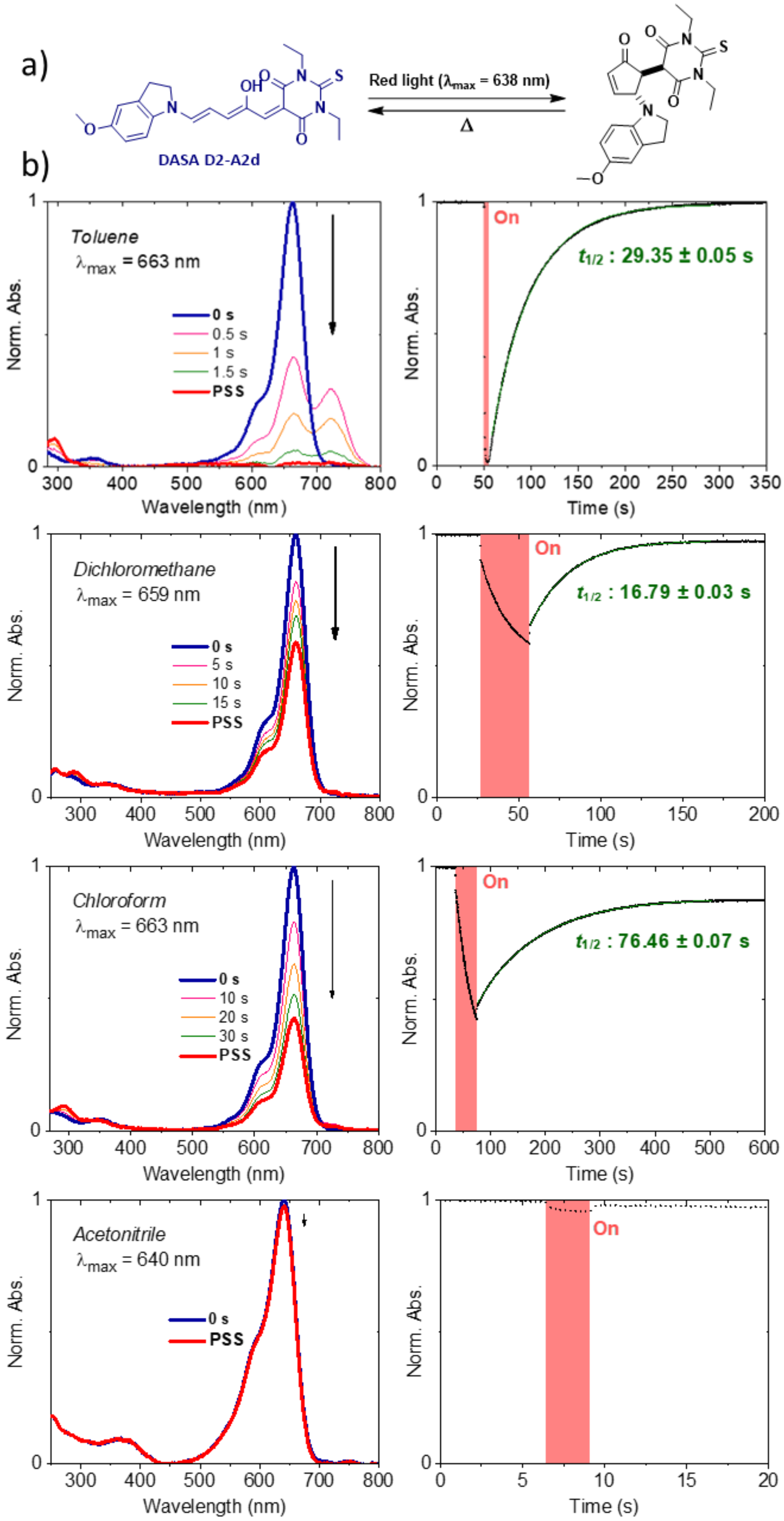

Figure S12. Photochromism of DASA **D2-A2d** in different solvents irradiating with red light (λ_{max} = 638 nm). (a) Chemical structures of both isomers. (b) Photochromic behavior at 20 °C showing full absorption profile over time until the photostationary state is reached (left) and peak absorption over time (right). Periods of red light irradiation are represented by red blocks.

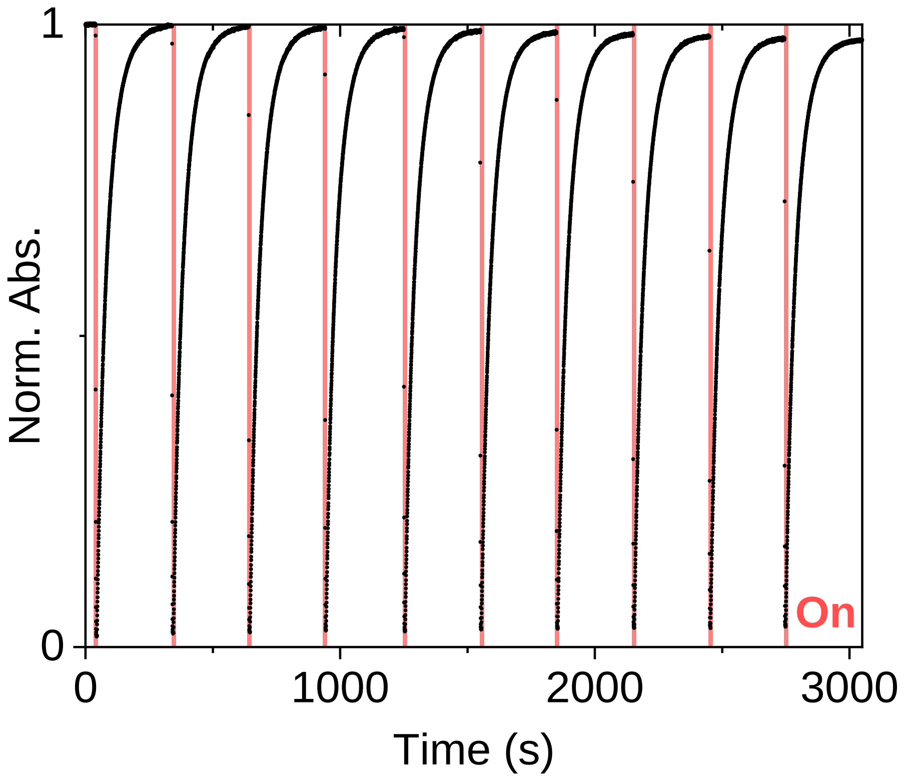

Figure S13. Fatigue resistance of DASA **D2-A2d** in toluene (20 °C). The absorption was measured at the absorption maximum (663 nm) and the red blocks indicate periods of irradiation (λ_{max} = 638 nm). This compound is relatively fatigue resistant showing less than 3 % fatigue over ten photoswitching cycles.

Pump-probe absorption experiments of DASA D3-A2d

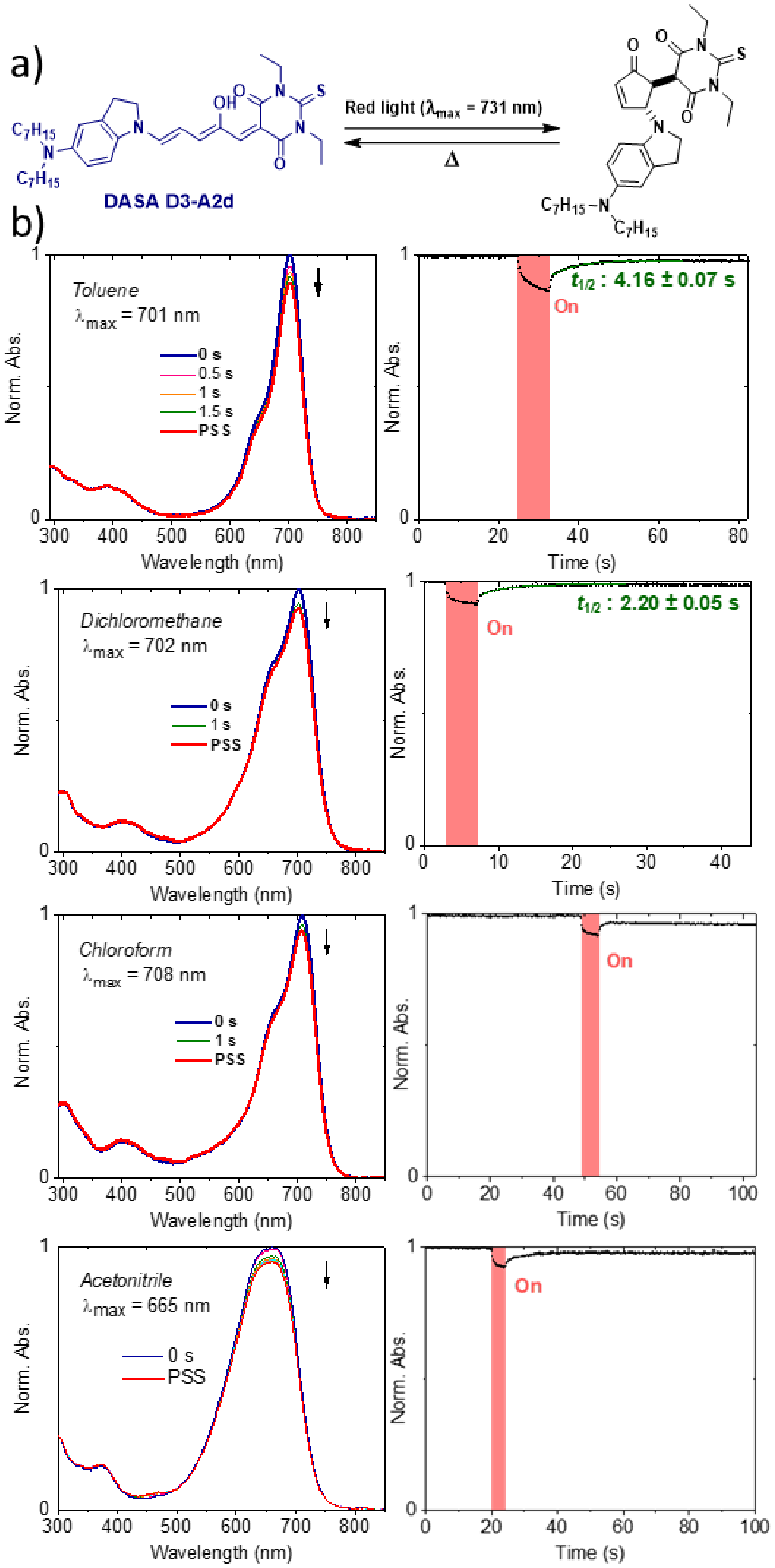

Figure S14. Photochromism of DASA **D3-A2d** in different solvents irradiating with red light (λ_{max} = 731 nm). (a) Chemical structures of both isomers. (b) Photochromic behavior at 20 °C showing full absorption profile over time until the photostationary state is reached (left) and peak absorption over time (right). Periods of red light irradiation are represented by red blocks.

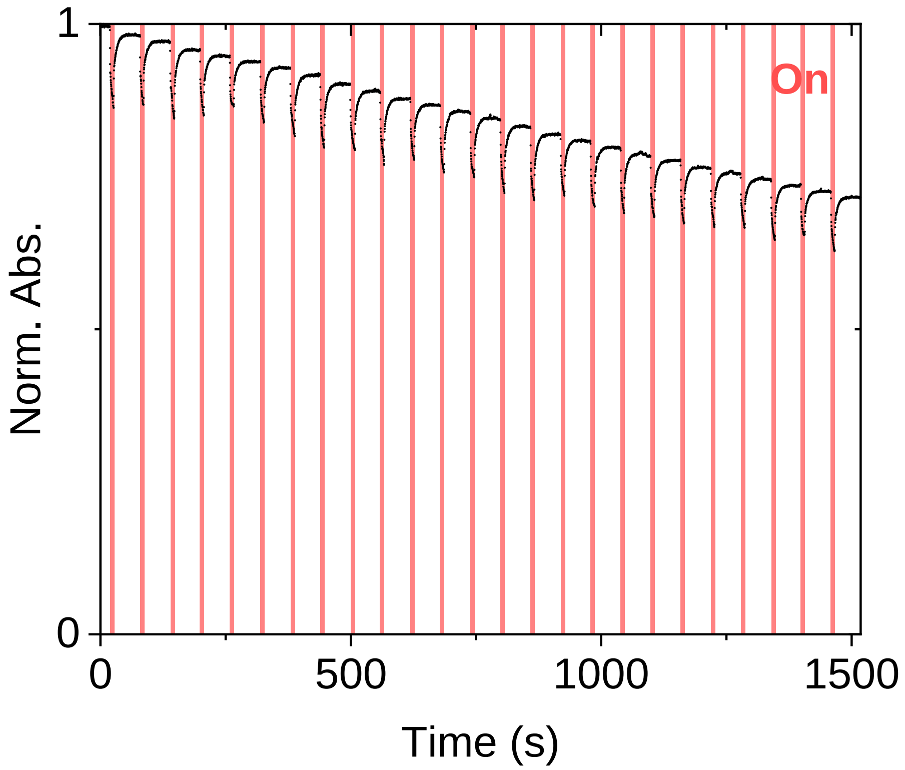

Figure S15. Photochromic cycling of DASA **D3-A2d** in toluene (20 °C). The absorption was measured at the absorption maximum (701 nm) and the red blocks indicate periods of irradiation (λ_{max} = 731 nm).

Table S2. Photoswitching properties for DASAs **D1-A2d, D2-A2d** and **D3-A2d** in different solvents.

	Solvent	Abs. Max. (nm)	PSS % [a]	Thermal Relax. % [b]	$t_{1/2}$ (s) [c]
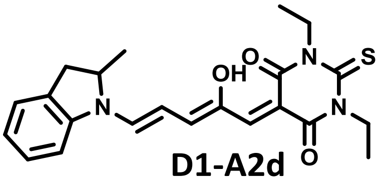 **D1-A2d**	PhMe	648	100	97	1261
	CH_2Cl_2	647	96	97	67
	$CHCl_3$	650	96	91	115
	MeCN	633	47 [f]	47	106
	MeOH	627	- [d]	- [d]	- [d]
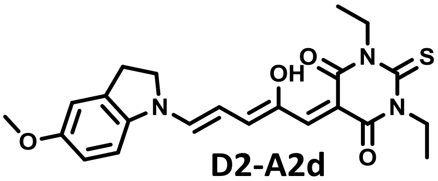 **D2-A2d**	PhMe	663	98	100	29
	CH_2Cl_2	659	42 [f]	93	17
	$CHCl_3$	663	58 [f]	78	76
	MeCN	640	4	- [e]	- [e]
	MeOH	633	- [d]	- [d]	- [d]
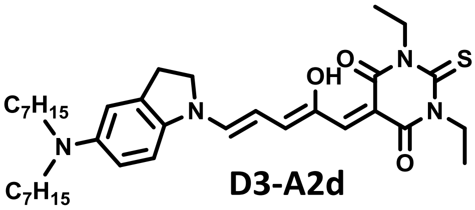 **D3-A2d**	PhMe	701	13	85	4
	CH_2Cl_2	702	8	88	2
	$CHCl_3$	708	8	- [e]	- [e]
	MeCN	665	7	- [e]	- [e]
	MeOH	629	- [d]	- [d]	- [d]

a) PSS is defined here as a measurement of the percentage of bleaching.

b) Thermal relaxation is defined as percent recovery from PSS to the original equilibrium.

c) Apparent half-life times $t_{1/2}$: time from PSS to halfway to dark equilibrium.

d) Spontaneous bleaching.

e) Not determined (change in equilibrium too small).

f) Irradiation during longer periods of time resulted in significant decomposition.

Thermodynamic equilibrium NMR studies

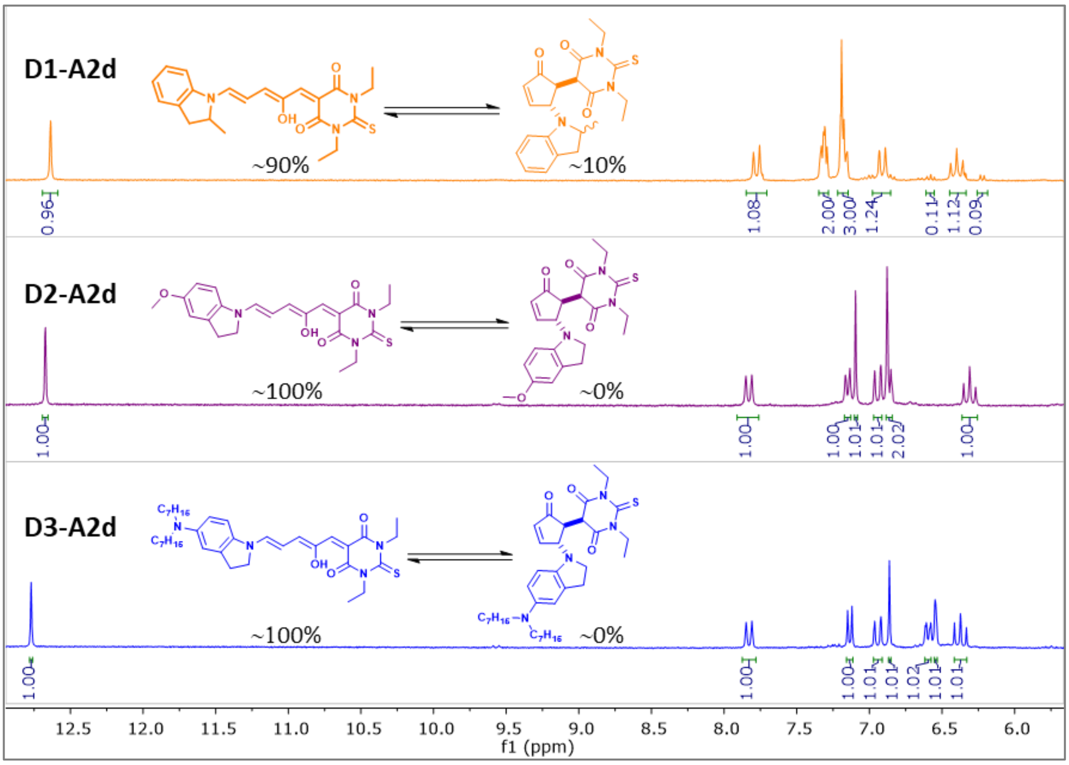

Figure S16. Thermodynamic equilibrium NMR studies of **D1-A2d**, **D2-A2d** and **D3-A2d** in CD_2Cl_2. Dark-adapted ^1H NMR spectra of the samples were recorded after being stored overnight at room temperature in the absence of light. The expanded section of the spectra used to estimate the equilibrium is displayed, showing an equilibrium of ~90% in the open form for **D1-A2d** and near to 100% for **D2-A2d** and **D3-A2d**.

NMR and HRMS spectra

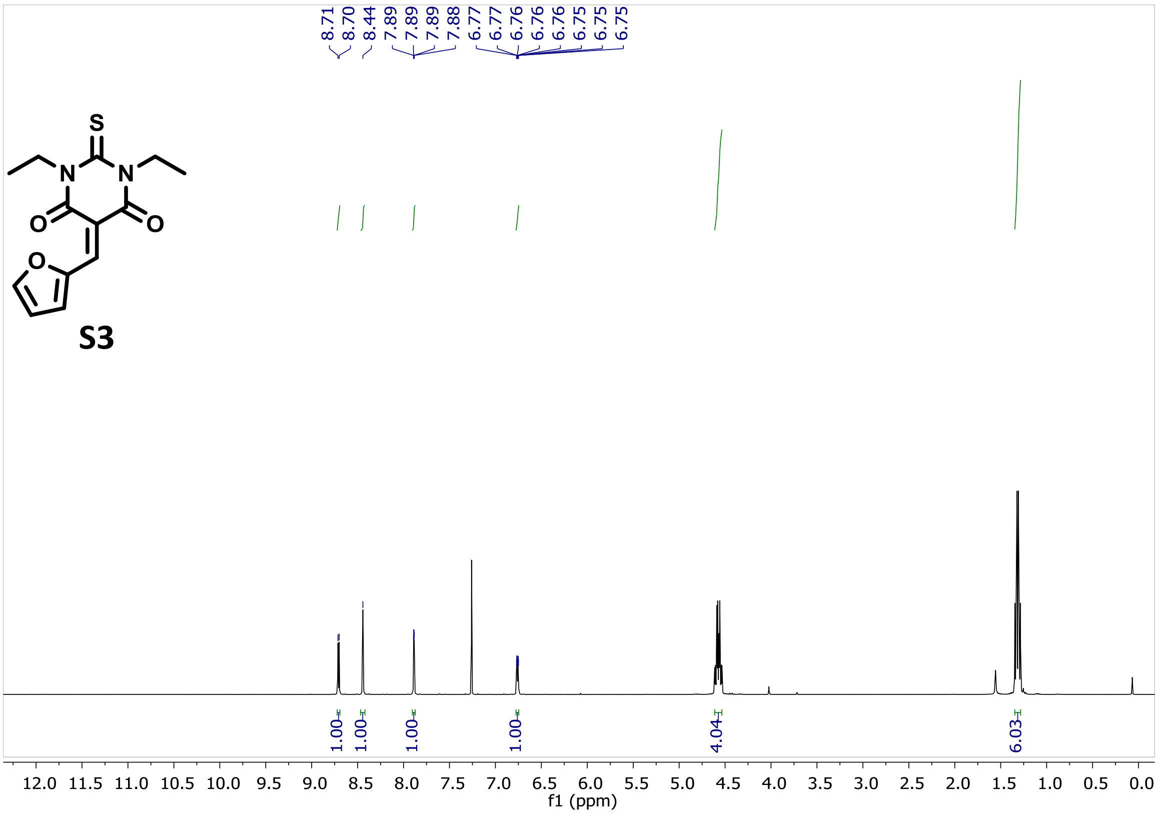

Figure S17. ^{1}H NMR spectrum of **S3** (300 MHz, $CDCl_3$).

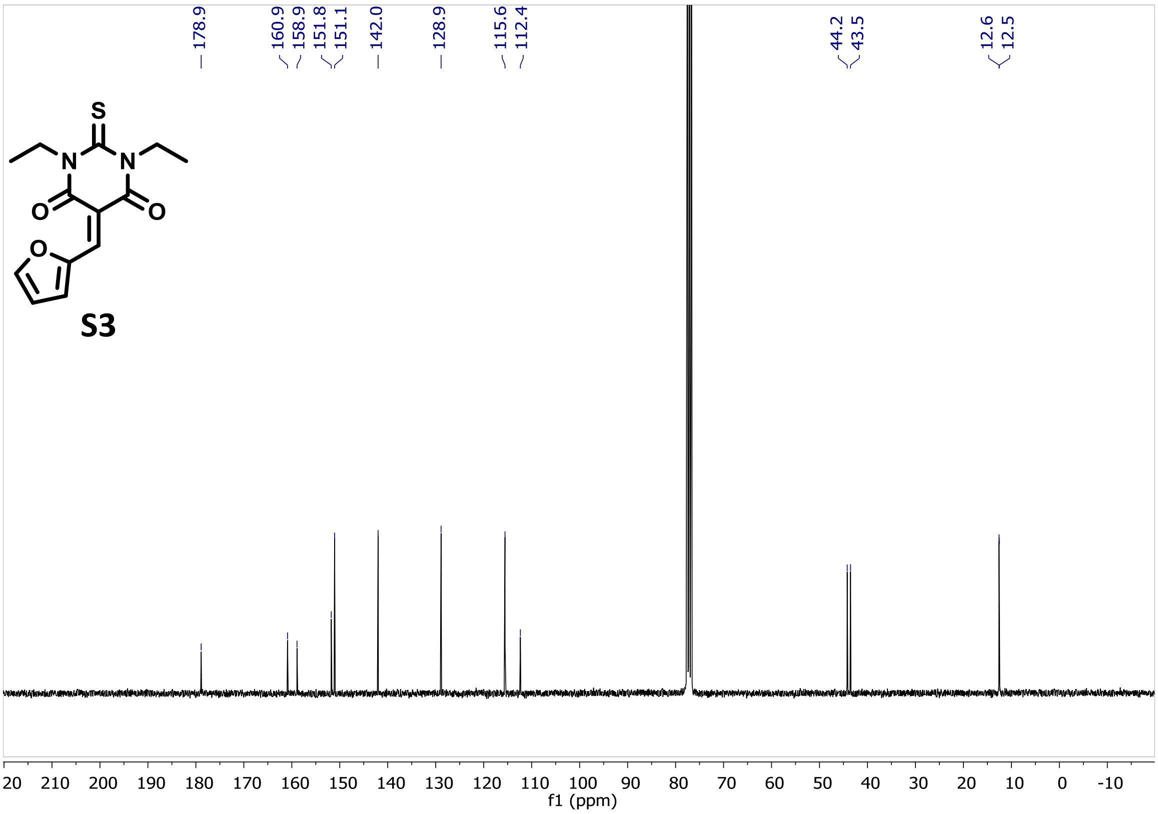

Figure S18. ^{13}C NMR spectrum of **S3** (75 MHz, $CDCl_3$).

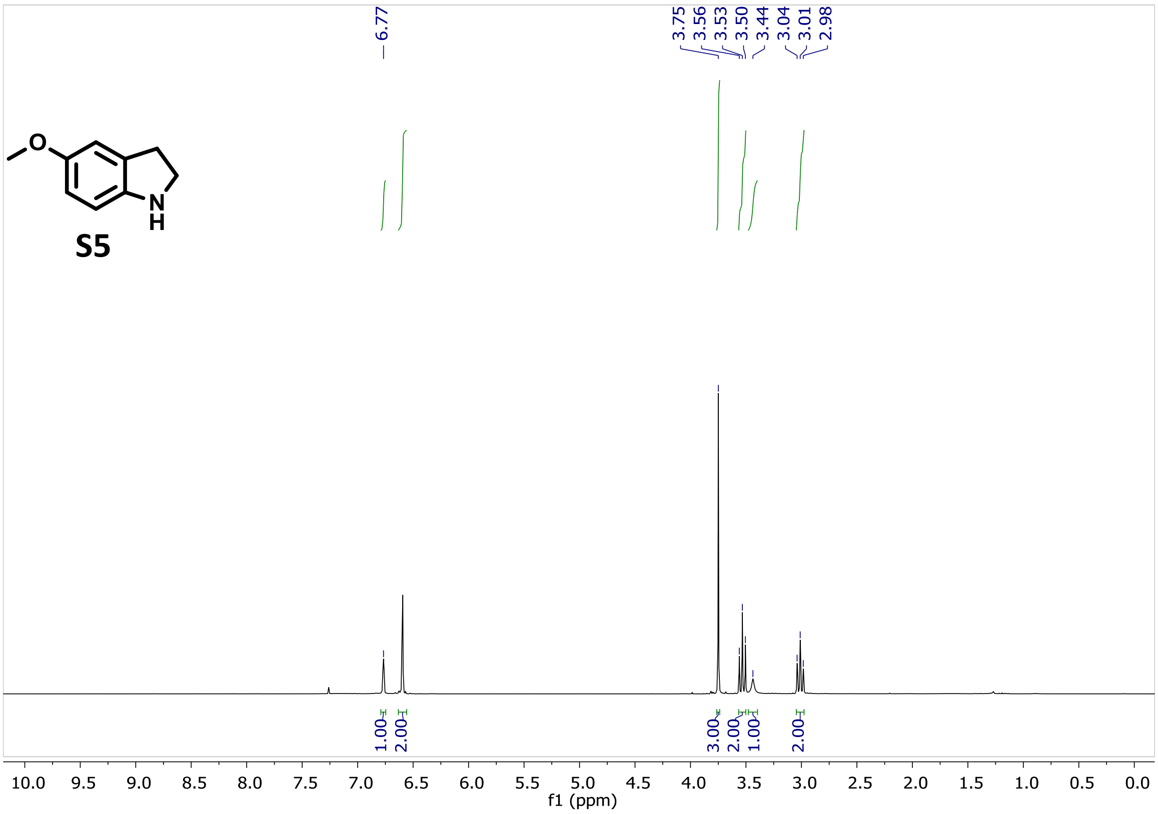

Figure S19. ^{1}H NMR spectrum of **S5** (300 MHz, $CDCl_3$).

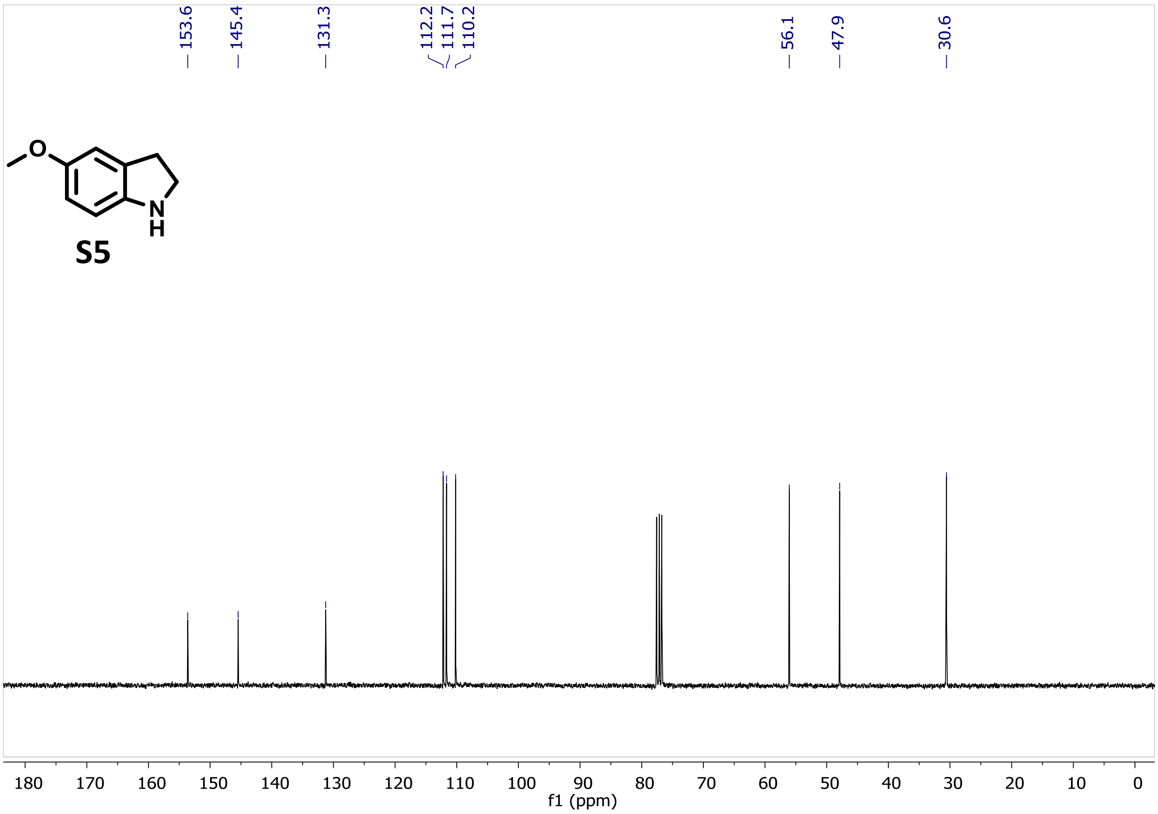

Figure S20. ^{13}C NMR spectrum of **S5** (75 MHz, $CDCl_3$).

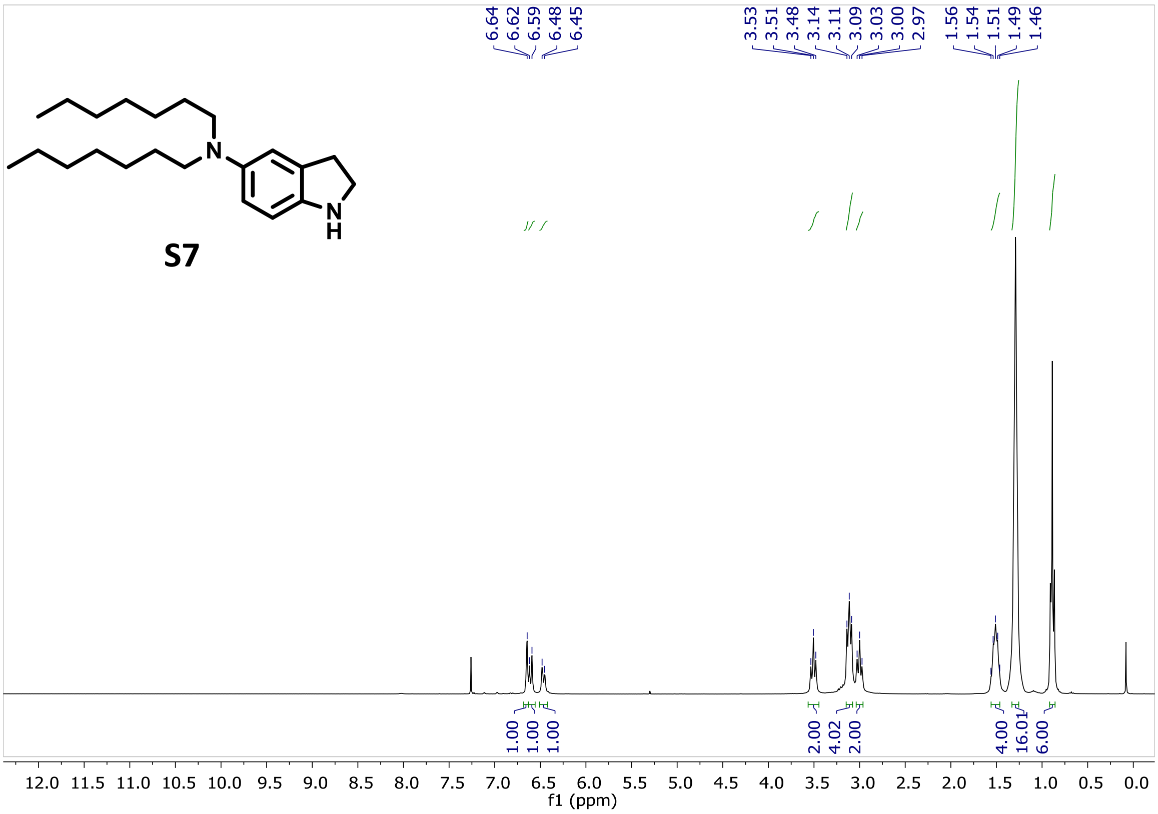

Figure S21. ^{1}H NMR spectrum of **S7** (300 MHz, $CDCl_3$).

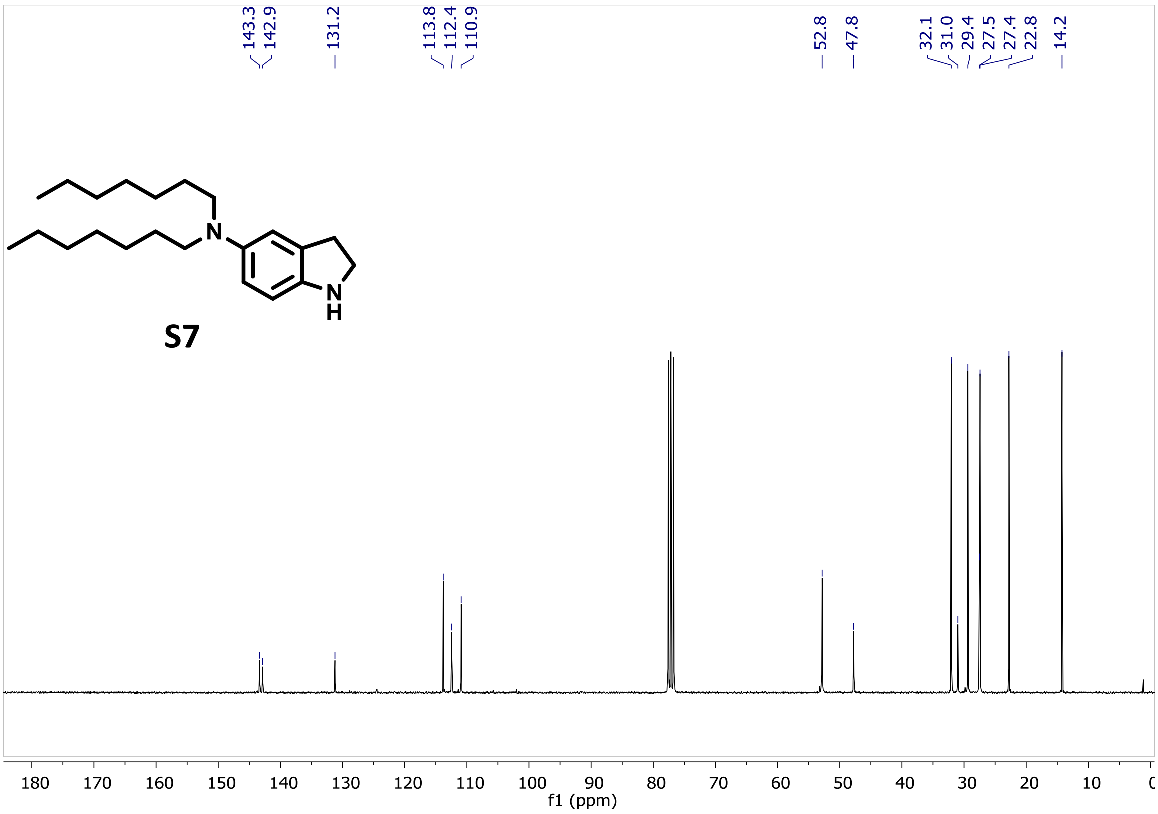

Figure S22. ^{13}C NMR spectrum of **S7** (75 MHz, $CDCl_3$).

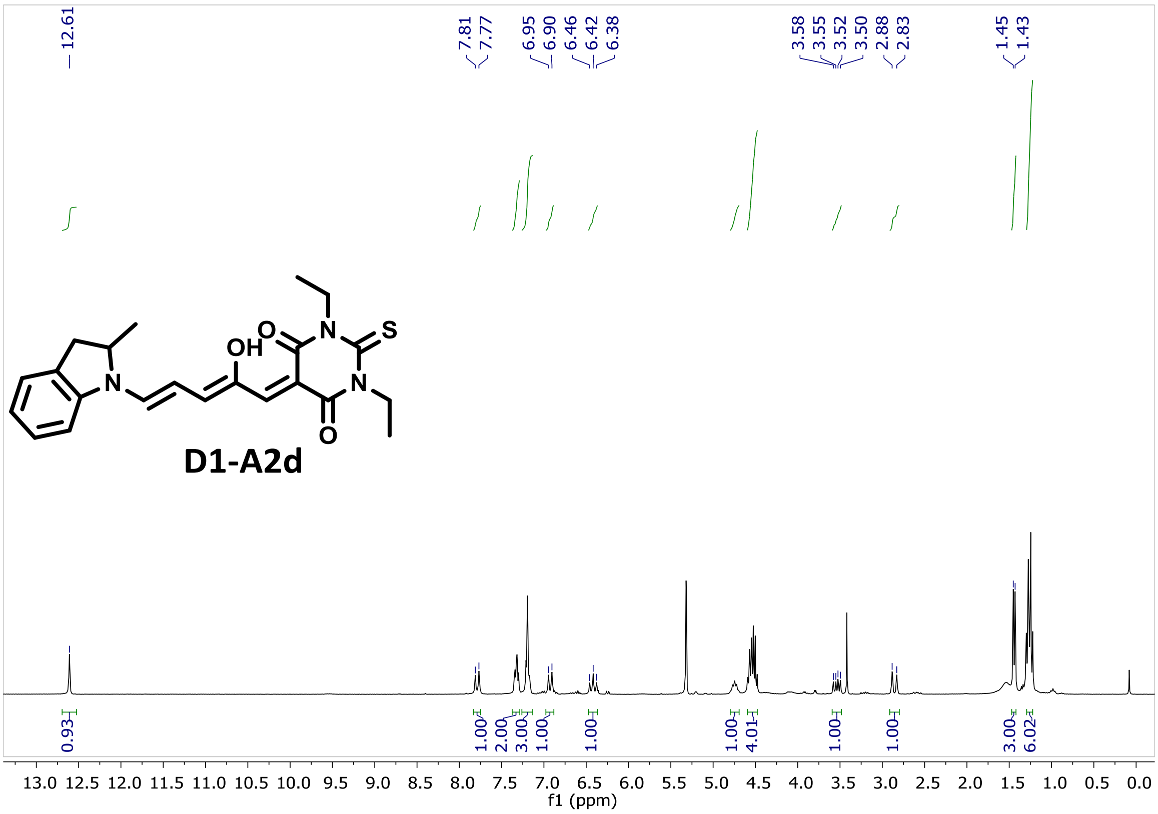

Figure S23. ^{1}H NMR spectrum of **D1-A2d** (300 MHz, CD_2Cl_2).

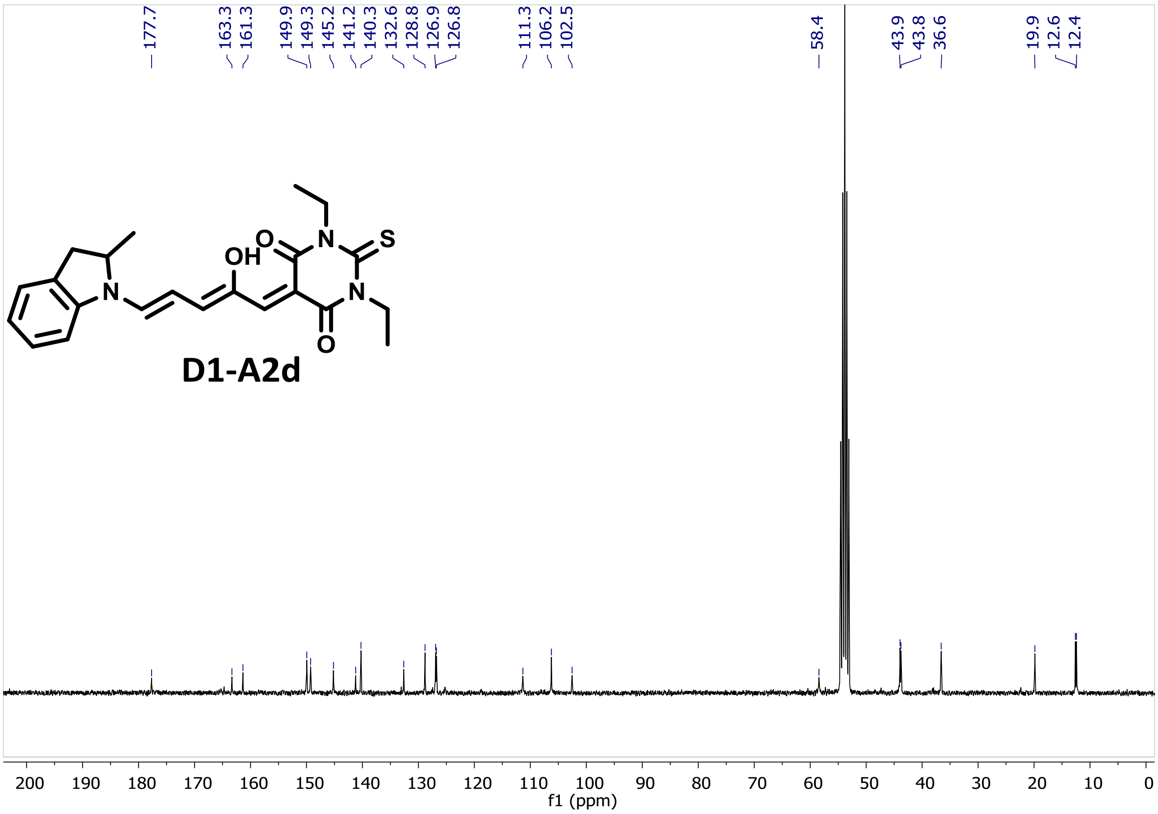

Figure S24. ^{13}C NMR spectrum of **D1-A2d** (75 MHz, CD_2Cl_2).

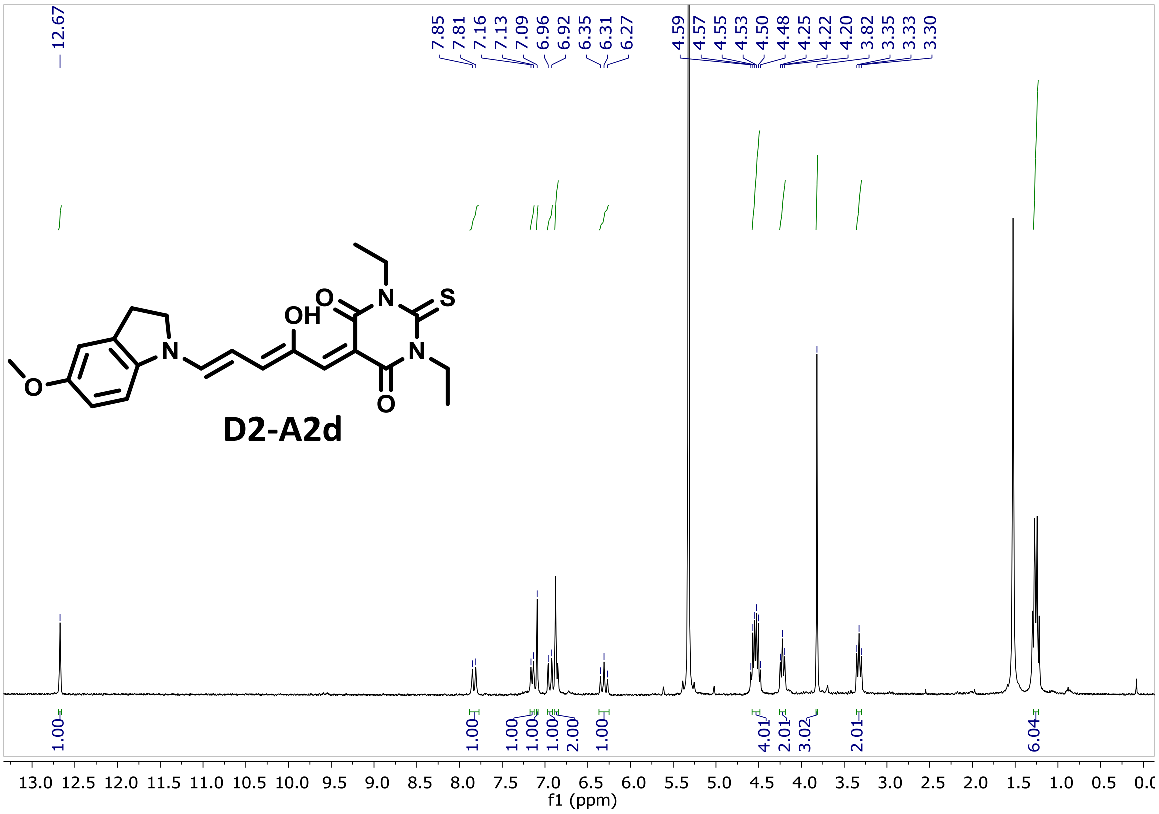

Figure S25. ^{1}H NMR spectrum of **D2-A2d** (300 MHz, CD_2Cl_2).

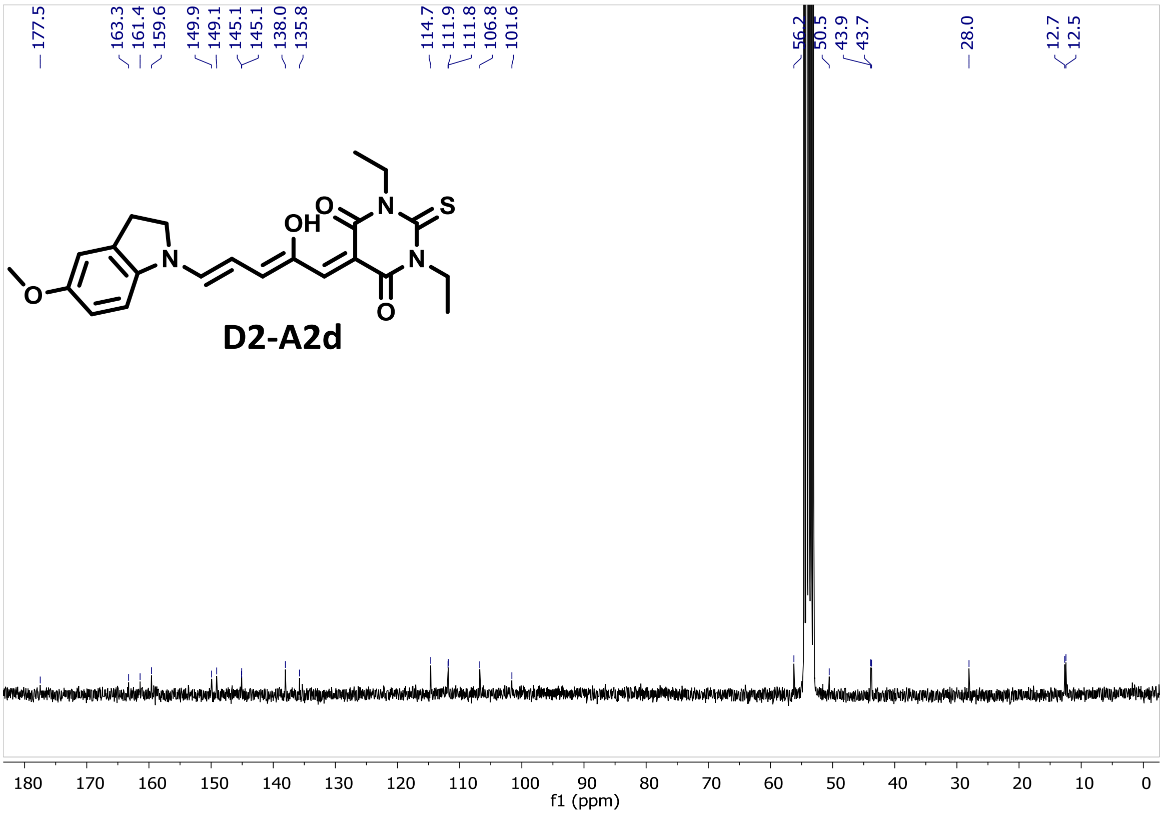

Figure S26. ^{13}C NMR spectrum of **D2-A2d** (75 MHz, CD_2Cl_2).

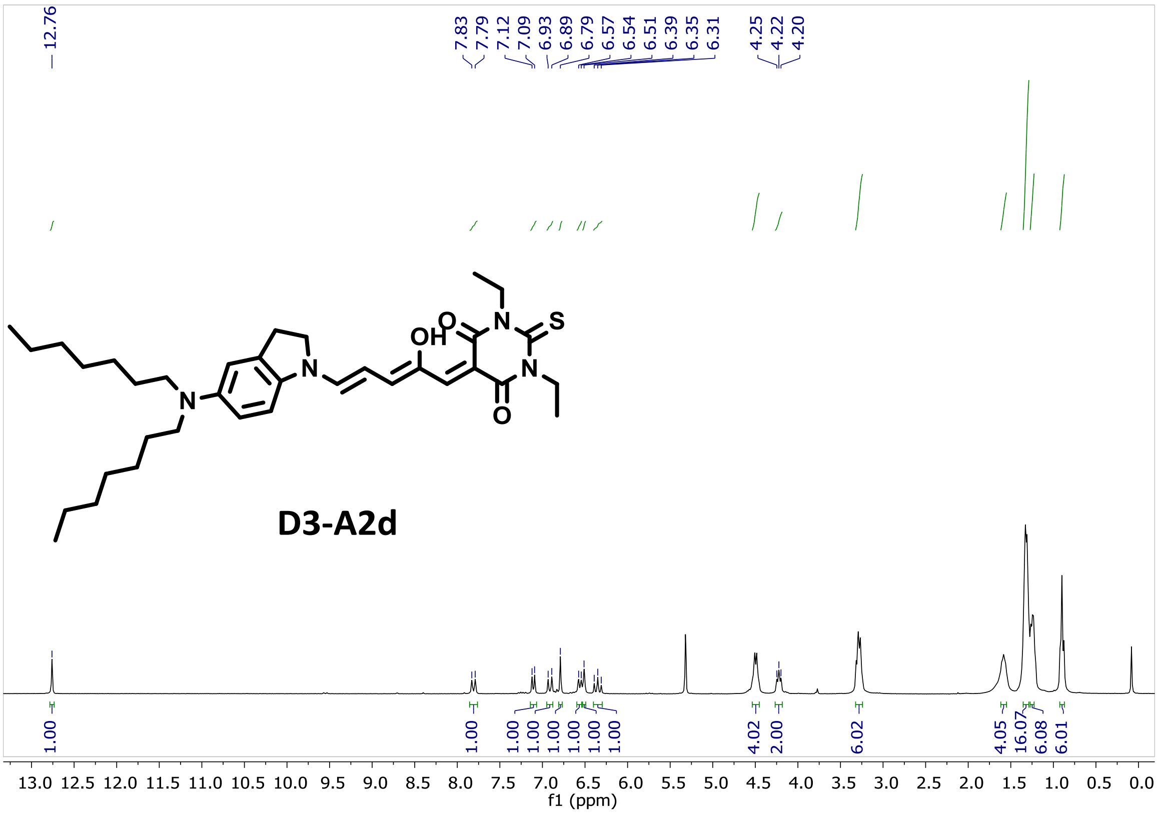

Figure S27. ^{1}H NMR spectrum of **D3-A2d** (300 MHz, CD_2Cl_2).

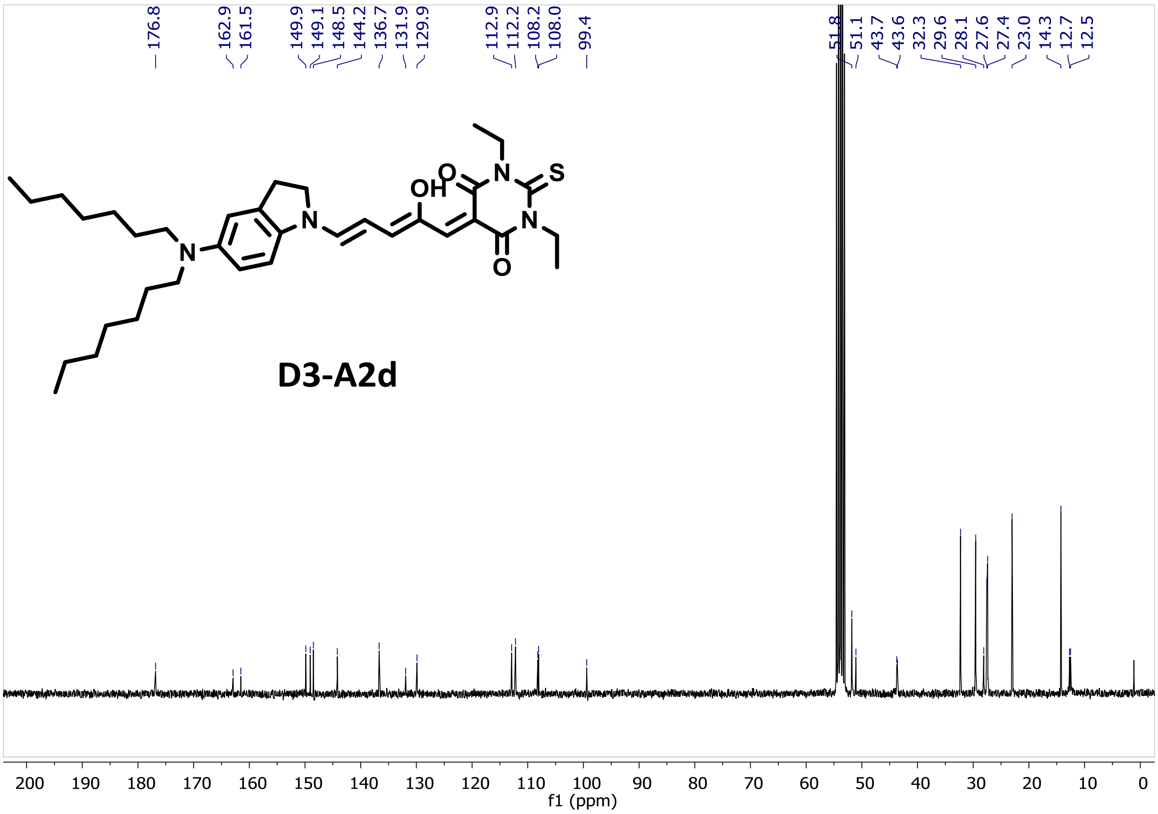

Figure S28. ^{13}C NMR spectrum of **D3-A2d** (75 MHz, CD_2Cl_2).

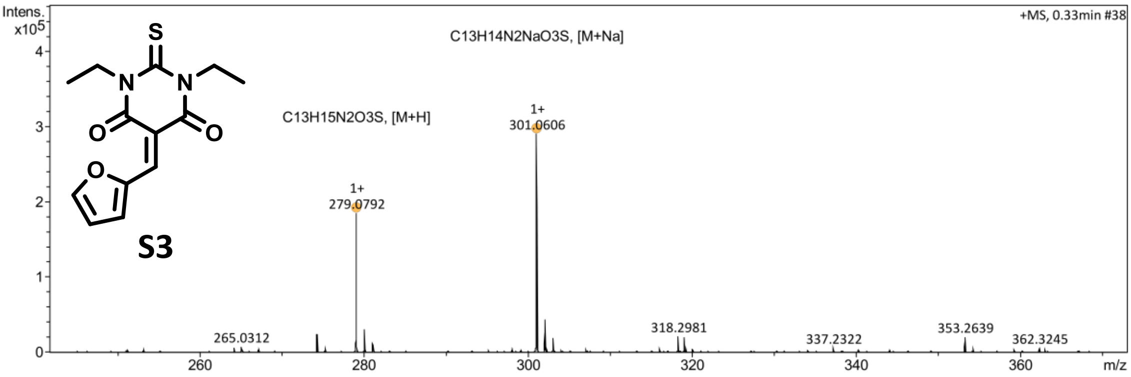

Ion Formula	m/z	z	|err| [ppm]	Mean err [ppm]	mSigma
C13H14N2NaO3S	301.061734	1+	3.8	3.9	3.1
C13H15N2O3S	279.079790	1+	2.3	2.5	4.1

Figure S29. HRMS (ESI+) spectrum of **S3**.

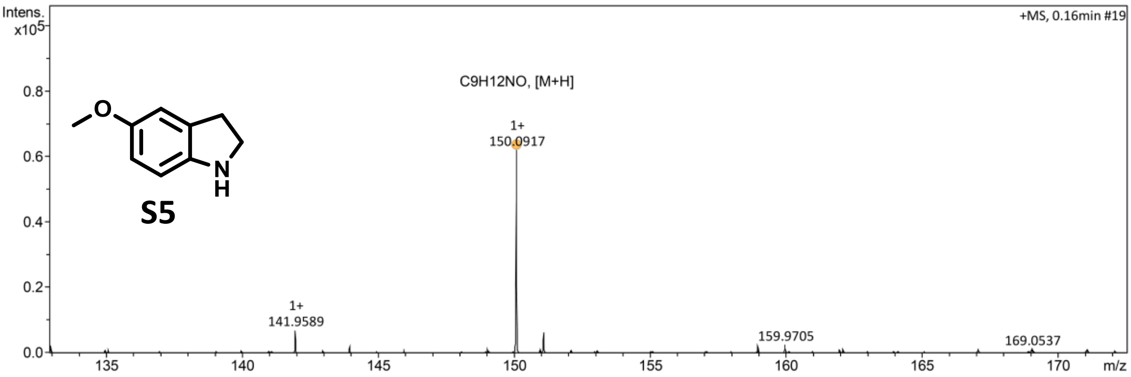

Ion Formula	m/z	z	|err| [ppm]	Mean err [ppm]	mSigma
C9H12NO	150.091340	1+	2.4	2.7	1.8

Figure S30. HRMS (ESI+) spectrum of **S5**.

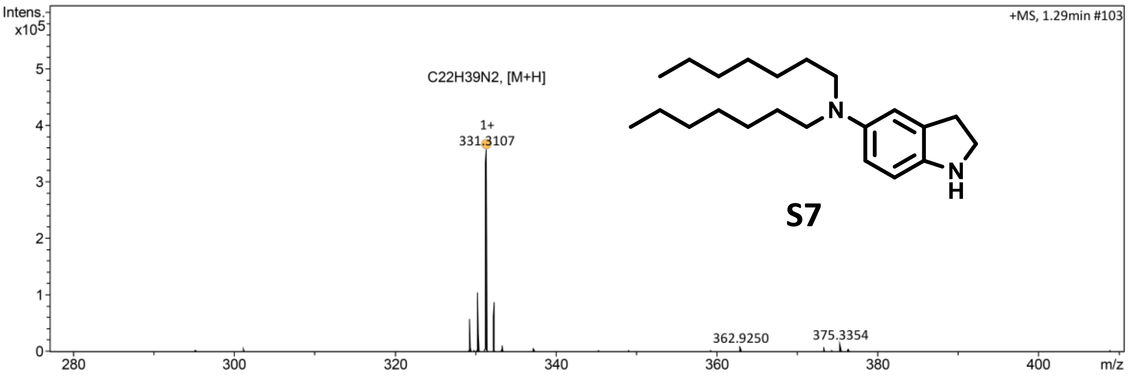

Ion Formula	m/z	z	|err| [ppm]	Mean err [ppm]	mSigma
C22H39N2	331.310776	1+	0.3	1.2	3.0

Figure S31. HRMS (ESI+) spectrum of **S7**.

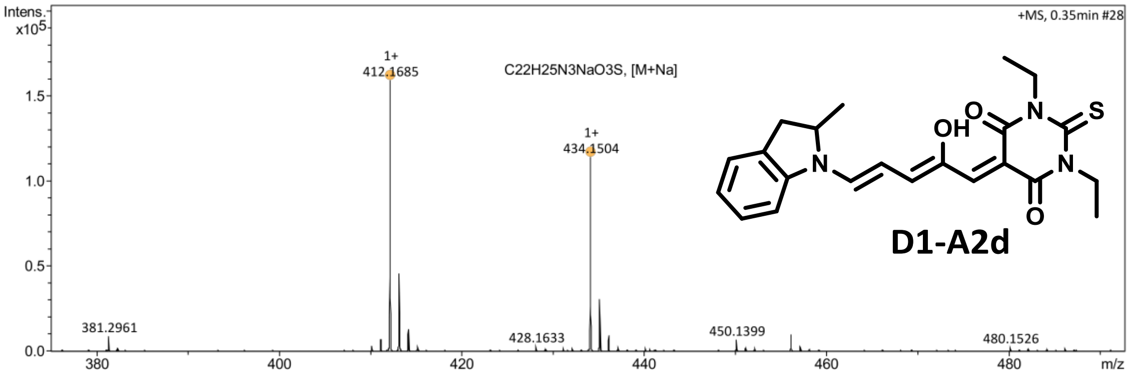

Ion Formula	m/z	z	|err| [ppm]	Mean err [ppm]	mSigma
C22H25N3NaO3S	434.150883	1+	1.2	0.7	4.2
C22H26N3O3S	412.168939	1+	1.0	1.3	12.9

Figure S32. HRMS (ESI+) spectrum of **D1-A2d**.

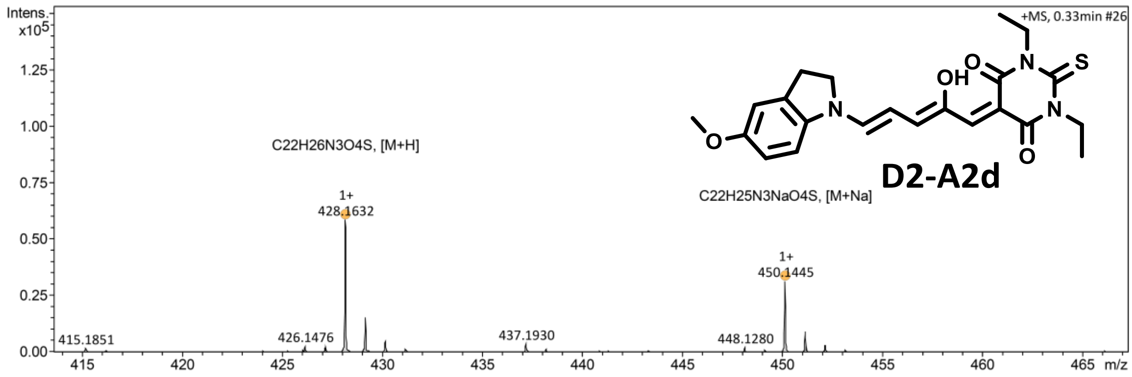

Ion Formula	m/z	z	|err| [ppm]	Mean err [ppm]	mSigma
C22H25N3NaO4S	450.145798	1+	2.9	1108.5	15.1
C22H26N3O4S	428.163854	1+	1.6	1.4	2.5

Figure S33. HRMS (ESI+) spectrum of **D2-A2d**.

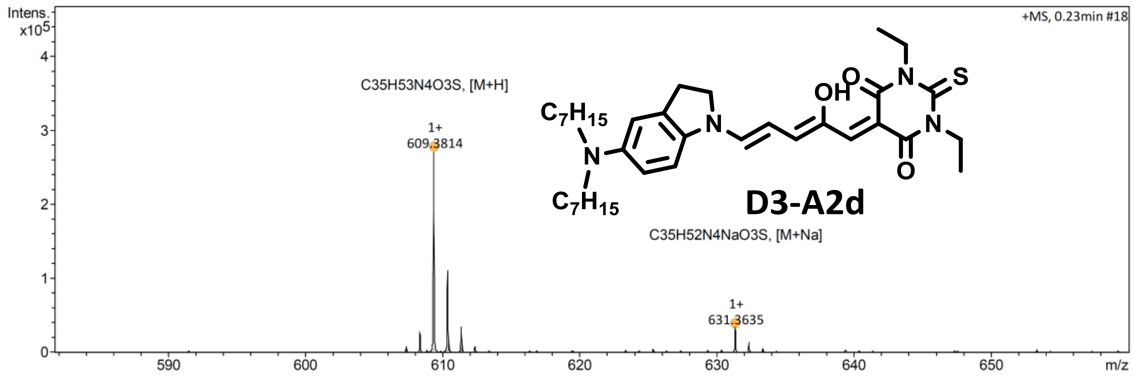

Ion Formula	m/z	z	|err| [ppm]	Mean err [ppm]	mSigma
C35H52N4NaO3S	631.365233	1+	2.7	3.4	3.1
C35H53N4O3S	609.383289	1+	3.1	4.3	2.3

Figure S34. HRMS (ESI+) spectrum of **D3-A2d**.

Cartesian Coordinates

Table S3. Cartesian coordinates of the DASAs studied. Geometries optimized at the DFT/D3-B3LYP level of theory with def2-TZVP basis set.

47			
D1-A1			
C	9.56782466509790	-5.05772815735444	-0.59373968548055
C	9.83750562568241	-3.73232889885954	-0.93970842641710
C	8.27806654665812	-5.44074920077801	-0.24015124417618
C	7.23688382063721	-4.51636913098676	-0.20428350005042
C	8.80997081335760	-2.80813246665897	-0.92226454401820
C	7.52467946932835	-3.20056005878754	-0.54437932659162
N	6.66419724096281	-2.09383520655270	-0.55702610694173
C	8.79747991890816	-1.34604165341157	-1.28997916660398
C	7.44666969423494	-0.84551290034283	-0.72573353320991
H	8.84281834469879	-1.22278356915788	-2.37550609265504
H	9.63358104109257	-0.79185081296105	-0.86392178256605
C	7.61055100108943	-0.11828980254060	0.60597178125705
H	6.92014701513131	-0.21199341132690	-1.43996691334333
H	6.64805947668053	0.13154838303505	1.05026981913193
H	8.16818268008123	0.80711997148263	0.45412499733656
H	8.16404421421660	-0.74362102354002	1.30917091055705
C	5.33013561465361	-2.14659534432731	-0.38769454204437
C	4.45205778414166	-1.09888527238516	-0.33022303578887
C	3.07825648035309	-1.34502161348772	-0.14860607730248
C	2.09527955291236	-0.38063364442400	-0.01080197691806
C	0.78276679181918	-0.85386302798012	0.20751503817125
C	-0.44517464295780	-0.25545938980403	0.43740752999053
H	8.07727539324178	-6.46948672444803	0.03010069760976
H	6.24687490009494	-4.82535127241940	0.10113760785381
H	4.93653039573055	-3.15129283848238	-0.29362711516141
H	4.78554302906630	-0.07317895115493	-0.40379114774675
H	2.75140527965586	-2.37562116144267	-0.09479126383959
O	2.45617636085575	0.91699227060006	-0.07367440961567
H	0.71671432534252	-1.93444085768998	0.21755245110978
H	10.36389482890194	-5.79013137852407	-0.59702195786396
H	10.83906118854819	-3.43309809265962	-1.22263925538797
C	-1.55056196941595	-1.19408310701004	0.69727586024133
C	-0.68064596434910	1.17251666186737	0.55218707717671
O	0.16908858287191	2.04852262827051	0.49882604066639
O	-1.44512381340993	-2.39328372462736	0.76587663503939
H	1.66449742157221	1.47792644469953	0.13262263988475
O	-1.95277234456309	1.57008744110362	0.79402145168408
C	-3.04229541673748	0.68732948388571	0.48673101228868
O	-2.77587738739095	-0.63821427054719	0.93213307107422
C	-3.29175097941368	0.69584110503138	-1.01445746964917
C	-4.22551345177298	1.17291898356563	1.29282159066210
H	-3.98723773754197	1.11310792034942	2.35370534266093
H	-5.09015234936861	0.54441283260136	1.08520192491350

H	-4.45827972939534	2.20450204088481	1.03103312990319
H	-2.41433460172416	0.33677276062508	-1.55336887512841
H	-3.52056867688958	1.70798951360043	-1.34672549154456
H	-4.13156043268923	0.04285852307024	-1.24876366916762
45			
D1-A1a			
C	9.86166658750116	-4.88636810611598	-0.44241804803238
C	10.02182813810911	-3.54963009099195	-0.81097969704614
C	8.60355949277191	-5.36875874852635	-0.09612385892421
C	7.48687927613480	-4.53627174572440	-0.09032504402211
C	8.91956284549898	-2.71648745915587	-0.82352183738670
C	7.66528696832531	-3.20719014069220	-0.45529943457911
N	6.71385867688234	-2.17980444597322	-0.51008896793736
C	8.79483538904367	-1.26997742758590	-1.22579983263942
C	7.38828851676578	-0.87392894688136	-0.71795625307739
H	8.86943189723151	-1.16503264717507	-2.31155997314022
H	9.56550165049120	-0.63788587351050	-0.78462918731528
C	7.45268730516121	-0.07521872518920	0.58023853232446
H	6.82834747721099	-0.32167377168581	-1.47294094451414
H	6.46246768545876	0.11313378129467	0.99185948876371
H	7.93474546845304	0.88580905718396	0.39340732893224
H	8.03935341939941	-0.61746806795594	1.32462541991209
C	5.38264510663033	-2.33359807352486	-0.36839549443153
C	4.43953145432892	-1.34239007398035	-0.37966048732935
C	3.07031580379822	-1.62606717191471	-0.22727939829110
C	2.08108596952789	-0.65339377866347	-0.18402332391848
C	0.75325910465173	-1.08997265765370	0.00294440047486
C	-0.47627872463808	-0.44703864029070	0.13357297569567
H	8.48754995045878	-6.40566338894914	0.19212141106593
H	6.52222302857869	-4.92287206974808	0.20779743753641
H	5.06198755096256	-3.35985087777754	-0.23772589291519
H	4.72223876954333	-0.30554990615728	-0.49611835656032
H	2.75550183976641	-2.65646970321976	-0.12313146819075
O	2.45916770130800	0.62951334450307	-0.31572296075339
H	0.65930867322061	-2.16801214540935	0.06569759529740
H	10.71643545389529	-5.54904131706344	-0.42260661456891
H	10.99766731605336	-3.17103434620919	-1.08883008678688
C	-1.63224084631503	-1.36609979044631	0.30223339703272
C	-0.68244327525568	0.99145905706189	0.15184846991637
O	0.22622101319772	1.82553874628526	0.03103355963678
O	-1.49562236090435	-2.57977242668829	0.34380324099191
H	1.65044386506027	1.20977864517573	-0.20102119776303
C	-2.08123344534081	1.52841314078616	0.37113771732688
C	-3.18278114913825	0.61263940381174	-0.14293095468098
C	-3.02595614515599	-0.78031980184918	0.45111954829659
H	-3.13652419666025	0.55985516369540	-1.23483028771238
H	-4.16000850924375	1.02680885948734	0.11438260074643
H	-2.11725255583777	2.51926771657334	-0.08234750673625
H	-2.19517550462764	1.67410057134278	1.45299482590634
H	-3.23750956753666	-0.75198601660914	1.52744764808688
H	-3.72325711476703	-1.49845910388310	0.01944151130936

51

D1-A1b

C	9.90695825964070	-4.86013775195507	-0.20619087138371
C	10.03398511629013	-3.56648057133444	-0.71825351626336
C	8.66560085221769	-5.32161659940315	0.21610499047899
C	7.53190680903323	-4.51359327897275	0.15180663196970
C	8.91694504024163	-2.75951082795200	-0.79951270273644
C	7.67553574461247	-3.22829908321232	-0.35859077221324
N	6.70873970216186	-2.23178170138084	-0.50156327724687
C	8.75386840077177	-1.36295235368509	-1.34272070131987
C	7.35624695615267	-0.94133446670593	-0.83016760163240
H	8.79117421031455	-1.36950877040486	-2.43579333759936
H	9.52637541149875	-0.67715563965696	-0.99474083544448
C	7.45079194329995	-0.03717170091101	0.39527250789673
H	6.76830023403012	-0.45680254919994	-1.61100715506876
H	6.47234601408939	0.16744554689122	0.82648781548227
H	7.90767060109235	0.91325685573187	0.11259337962408
H	8.07342081889285	-0.50690327260475	1.15951237038079
C	5.36992444994531	-2.39766855152160	-0.34122611477829
C	4.42289998958730	-1.42175665566412	-0.37062085395570
C	3.03595405448018	-1.70188888868121	-0.25812268846014
C	2.06325474107882	-0.73280306338093	-0.19648702982142
C	0.69570616478937	-1.15387676384091	-0.13954757360354
C	-0.50520654717599	-0.50614412061251	-0.05793278312660
H	8.57511972032295	-6.32191735689750	0.62031663505804
H	6.58130381031059	-4.87914969545113	0.51499726980373
H	5.06754662948841	-3.42647015131235	-0.18668464275105
H	4.70367020857019	-0.38378620358759	-0.48346381435228
H	2.71632885677366	-2.73565433705314	-0.21426737170530
O	2.44749270000925	0.55640086638338	-0.21443088373117
H	0.62486946039079	-2.23188481069611	-0.19039899495688
H	10.77528945130261	-5.50083955207480	-0.12912094086973
H	10.99759076705443	-3.20223338143723	-1.05327717027944
C	-1.80288283873370	-1.28091191216819	-0.13072607405694
C	-0.68217761688899	0.93871655986390	0.08320425265067
O	0.19796187343437	1.78558520727408	0.00142739917484
H	1.64488261730783	1.14049545149661	-0.16510876990764
O	-1.92649203554888	1.40721074436933	0.30954455653173
C	-3.07390458847875	0.57265793967475	0.64317600170520
O	-2.69471400599441	-0.75337224273962	0.86174115466721
C	-4.06597962311565	0.72348137480448	-0.50375962962882
C	-3.62087758965391	1.09458639490545	1.95758688870758
H	-2.85464324853684	1.00635260370313	2.72651405378886
H	-4.48807099243991	0.50395118872558	2.25163427405322
H	-3.91525517596222	2.13886353844003	1.85987820262820
H	-3.64461820539725	0.36685966204618	-1.44245334348866
H	-4.32878208036565	1.77289935406334	-0.63131211357408
H	-4.96728687674512	0.15330159161270	-0.27894148256161
C	-1.72792329917153	-2.77252279079485	0.11382589870858
H	-2.25094354926843	-1.12298793090491	-1.11988960886758
H	-1.28851826850646	-2.98312356895153	1.09009458508546

H	-1.14161422202547	-3.27422786854500	-0.65453713638206
H	-2.73722084517698	-3.18241646629167	0.08953092337151

48
D1-A2

C	9.93436260751520	-4.73793777225214	-0.51525984650950
C	10.03142365941920	-3.35249878012066	-0.65121060611620
C	8.68654943182440	-5.33898436625261	-0.38004154521143
C	7.51675426256283	-4.58174565667477	-0.36459631382652
C	8.87881896700689	-2.59068106504314	-0.64516435797951
C	7.63204383007853	-3.20197280002035	-0.48998210927680
N	6.61980964403203	-2.23191543533642	-0.49671314690441
C	8.69339078148305	-1.10952449896228	-0.83833440755062
C	7.22057961612391	-0.87522206619687	-0.43447011856879
H	8.86873827215143	-0.83297666481100	-1.88158149214709
H	9.36853171678193	-0.51068579548960	-0.22642210441928
C	7.08960470959174	-0.30241684151357	0.97466102859545
H	6.70706734256550	-0.23593418501255	-1.15145436153091
H	6.04861455774253	-0.25357933560919	1.29100560743267
H	7.50691009845785	0.70535154950069	1.00802285507809
H	7.63932481615743	-0.92492238844753	1.68341989798199
C	5.29104821264234	-2.46573283446151	-0.46103591049724
C	4.30989069451925	-1.51713055437728	-0.55670137030714
C	2.94001697229397	-1.81163770927019	-0.42570245469552
C	1.94955058827373	-0.85037232163325	-0.57765107642123
C	0.62757735459715	-1.20312460223885	-0.23059440968174
C	-0.56768294976288	-0.50042515060854	-0.12705357698670
H	8.61900150986309	-6.41421861825150	-0.27669884010473
H	6.55745067307682	-5.06705545452340	-0.25071545740787
H	5.01574472995359	-3.50730034003056	-0.35066132578755
H	4.56841483048377	-0.48205449638716	-0.73266416657754
H	2.63119008694818	-2.81321989516656	-0.15611381135430
O	2.33589503160598	0.37152884254680	-0.97907436324984
H	0.52466292871392	-2.23459711329062	0.08300478484010
H	10.82898344168962	-5.34592248681319	-0.51596336950680
H	10.99883636237548	-2.87958502156525	-0.76743784911889
C	-1.68395589096279	-1.26355833437190	0.45087444051491
C	-0.76878622436132	0.87827066370982	-0.52476504700182
O	0.10559821377900	1.59923561331086	-1.02345662861519
O	-1.60422352364298	-2.43560470092310	0.78320852234595
H	1.52965130075387	0.95655460721414	-1.04294382647245
N	-2.03804112266716	1.41171996720125	-0.34882250890961
C	-3.10492813001117	0.73838337190475	0.23879861035725
N	-2.88250778998682	-0.56450107677457	0.63994012842931
O	-4.18116595657759	1.28193315839009	0.38867816926083
C	-2.31328128027793	2.79189186466175	-0.75890035622086
H	-1.41591157700820	3.18633099975545	-1.21971837128398
H	-2.59252277796639	3.38800748887280	0.10838208320605
H	-3.14283764080978	2.80751893307276	-1.46282765820661
C	-4.01317197977494	-1.24305608716480	1.27454549179829
H	-3.64151100727129	-2.16643227655578	1.70382773315414
H	-4.78742869934902	-1.45979273267213	0.53875941416012

H	-4.43625069463386	-0.59690760131833	2.03941402129352

48
D1-A2a

C	9.91629940612932	-4.74399086675700	-0.50897294532194
C	10.02274820531901	-3.35839113465888	-0.63188384399457
C	8.66377069707627	-5.33974639687651	-0.39452041368237
C	7.49748582828389	-4.57668949108393	-0.38927035848176
C	8.87385175683162	-2.59075134332429	-0.63496834678000
C	7.62208202171432	-3.19674621197888	-0.50284690001334
N	6.61529709081053	-2.21836503856754	-0.51679825002056
C	8.70060735431875	-1.10678699995018	-0.81514792999602
C	7.22702794601974	-0.86769566906180	-0.42085806317354
H	8.88586661402424	-0.82179617308239	-1.85437096848962
H	9.37465536318168	-0.51871723660186	-0.19185228079917
C	7.08574043372855	-0.32923891908149	1.00081807167433
H	6.72559230416039	-0.20894375021504	-1.12815709555432
H	6.04185426900633	-0.28519922335238	1.30865874800809
H	7.50572123782283	0.67586345698972	1.06312568955382
H	7.62670186744597	-0.97128841493973	1.69877733670224
C	5.28611062946670	-2.43672577376007	-0.50379457526835
C	4.31519334139978	-1.47417762402423	-0.61034846457221
C	2.94632631876987	-1.76047276916910	-0.48969593440876
C	1.94737472786136	-0.79911052415853	-0.62918758062802
C	0.64426106806713	-1.17703429899578	-0.25523438763586
C	-0.57095980083128	-0.50354436619965	-0.10106112248241
H	8.59010757468963	-6.41535257448467	-0.29999036573891
H	6.53446902496531	-5.05818627467856	-0.29138016805126
H	4.99581433848752	-3.47545561787489	-0.40358645686138
H	4.58644832394498	-0.44173065719228	-0.78218737244791
H	2.63655973847863	-2.76174821292140	-0.21962719397586
O	2.32493947004239	0.43466388709539	-1.00661098342708
H	0.58672635814965	-2.20542720211556	0.07719138095530
H	10.80782487341903	-5.35655870326176	-0.50268161037102
H	10.99400245757682	-2.88944395344827	-0.73046756366597
C	-1.63141915644087	-1.18668859188824	0.62979170672971
C	-0.81130379007137	0.82215775265421	-0.63861775173260
O	0.00792237710337	1.47325793940997	-1.30042770356941
S	-1.43480079578445	-2.65620534742814	1.39718772410438
H	1.49836210599272	0.96719983677184	-1.17099138251773
N	-2.05725718928156	1.38445742770736	-0.40508140823957
C	-3.09538416292040	0.75189377110608	0.26030804349887
N	-2.85712752203037	-0.54433298884975	0.70554608570759
O	-4.15647400026188	1.31869095088725	0.43243300401962
C	-2.34731206591754	2.74129985364467	-0.87816512938122
H	-1.44897904998848	3.12908716414649	-1.34195109904180
H	-2.65249312810674	3.36051024524298	-0.03719483768973
H	-3.16430482825210	2.71734559570437	-1.59741178350654
C	-3.98764854909805	-1.20072348987964	1.36784711947485
H	-4.07193082521395	-2.21754064869484	0.99477280844614
H	-4.87655515942360	-0.62278974966254	1.14648392660775
H	-3.82196510066615	-1.24530164314034	2.44423062603814

48			
D1-A2b			
C	9.90149728812197	-4.70811925178302	-0.10285724802027
C	9.99813024501927	-3.36891692784463	-0.48244213295869
C	8.65543556984071	-5.27238780683625	0.15376482831435
C	7.48835021065171	-4.52064367334838	0.05247376118747
C	8.84599497307678	-2.61406933967132	-0.59890709052373
C	7.60388326128665	-3.18823150555324	-0.32554830489344
N	6.58618163456114	-2.24325632117494	-0.50333583157797
C	8.65825246946570	-1.18818904880265	-1.04769066612732
C	7.16468289035316	-0.89963208606924	-0.76401224795819
H	8.89108660171262	-1.08142839820538	-2.10990976809456
H	9.29786334543831	-0.49114095790382	-0.50504559905445
C	6.97709384624667	0.03034681800654	0.43007518799806
H	6.66714690059459	-0.48438757501708	-1.64121012353653
H	5.92789802565308	0.15219609737193	0.69395616343252
H	7.38562687337548	1.01344429590954	0.19024066933880
H	7.50922560757621	-0.36237664288211	1.29892590206700
C	5.26510362456304	-2.50101799095676	-0.46763820697080
C	4.26894274727885	-1.59665691139612	-0.70828990914397
C	2.90664194286391	-1.91523491695875	-0.59043377080818
C	1.91454004960369	-0.96540529704073	-0.79163398376499
C	0.59646669141511	-1.30625505564512	-0.43276512961335
C	-0.57508675587683	-0.57472589785945	-0.26236239657523
H	8.58803021394191	-6.31133685752336	0.44948000553772
H	6.53084220812152	-4.97221462264245	0.27156685793817
H	5.00678605650252	-3.52328420562745	-0.22177541119318
H	4.50585273471802	-0.57738429301947	-0.97673816589141
H	2.60543014254405	-2.90799128999019	-0.28223253424777
O	2.30605338816623	0.23284673963690	-1.25477583937194
H	0.46840408027411	-2.35327310287830	-0.18731257078706
H	10.79531897871903	-5.30935729259882	-0.00602859123022
H	10.96476408674922	-2.92830891255643	-0.69241629425344
C	-1.74067837189232	-1.35784416694186	0.14005902712347
C	-0.68870412404911	0.85640253669562	-0.38990336816289
O	0.20501804169696	1.59448315677243	-0.82630826896859
O	-1.74287227255618	-2.57625086092011	0.21336797551313
H	1.53887690734194	0.86680182371229	-1.17736470396750
N	-1.87727515443307	1.44274761385181	0.04573113851379
C	-2.96777557163035	0.73868876943037	0.52482905502818
N	-2.90703737115954	-0.63209149821609	0.44993263325311
S	-4.28972529565058	1.52279768158763	1.15611734830494
C	-1.95668402984768	2.90674636758860	0.02806923711502
H	-1.05753625462675	3.27845217729545	-0.44648376685963
H	-2.04187601968004	3.28560003386875	1.04467786640609
H	-2.84052238090320	3.21600134869079	-0.52394937273178
C	-4.09410995698457	-1.40902398837206	0.81327999842283
H	-3.90438457721884	-2.43697900003029	0.52794623217173
H	-4.95765578843794	-1.00473403491508	0.29208324398116
H	-4.28164771252743	-1.34192572923756	1.88460416563955
48			

D1-A2c			
C	9.99166785344890	-4.59401664510913	-0.46338042578389
C	10.03493071619907	-3.20174242602054	-0.54462357783894
C	8.76760421582619	-5.25440118030264	-0.40645218419641
C	7.56635816513127	-4.54904236621016	-0.42042478025665
C	8.85071981746330	-2.49034573516352	-0.56589581278131
C	7.62841923983372	-3.16268472283007	-0.49408702099195
N	6.57470415096656	-2.23712733185379	-0.51836444638470
C	8.60683834215178	-1.01189624715463	-0.70882360164577
C	7.11246318541704	-0.86099284549193	-0.35552193040619
H	8.80829918062873	-0.68670943664078	-1.73313829173566
H	9.22931238471837	-0.40869976541235	-0.04755201068782
C	6.89702174995803	-0.38818914954605	1.08021551072824
H	6.60050451794897	-0.20175597767298	-1.05439311514625
H	5.84409661774724	-0.42680404285519	1.35741942074763
H	7.24594275489996	0.63973968577348	1.19117456119874
H	7.45989659500681	-1.01935624131236	1.77035943407972
C	5.26015542658568	-2.51744182363359	-0.55896847710236
C	4.26024612787361	-1.58725082435319	-0.67986247637914
C	2.89976833493061	-1.90882790866506	-0.60055188447744
C	1.89615886527583	-0.95203401006945	-0.74068281205318
C	0.59109313290418	-1.33725024559291	-0.39133086517817
C	-0.60412772720861	-0.63170693209506	-0.22268577178064
H	8.74419985055079	-6.33435754258222	-0.34319547698999
H	6.62443995882409	-5.07722355376222	-0.36799320140659
H	5.00951689244513	-3.56900612668072	-0.49207545298600
H	4.50353083551109	-0.54423485458150	-0.82515817472764
H	2.59825093988652	-2.91864769124385	-0.35430401765809
O	2.28225360615936	0.29412832781432	-1.07085837349881
H	0.51779092617681	-2.36972339907986	-0.07560823372492
H	10.91171814597591	-5.16231990948261	-0.44419801346388
H	10.98455330442609	-2.68380795298632	-0.59736171823745
C	-1.68042024383464	-1.26023112988888	0.51971511128007
C	-0.80370211683117	0.68313577716498	-0.78331626724587
O	-0.02477200660668	1.22152024265704	-1.58245711208133
S	-1.57758274075258	-2.79294407886976	1.17362467285319
H	1.46427384574802	0.79641858522114	-1.33864858348528
N	-1.94553782354825	1.37702066581540	-0.39792826815715
C	-2.90321837734998	0.86391493404783	0.45184394578830
N	-2.82836500092721	-0.49214319134365	0.71480604277141
S	-4.07648469449860	1.83790338390376	1.11051934053327
C	-2.08613394886387	2.76019267052304	-0.86578520532993
H	-1.37719459709169	2.90448193573650	-1.67121312542231
H	-1.88021850652830	3.45374740335238	-0.05162571511056
H	-3.10534555201036	2.92167909766011	-1.20537404077825
C	-3.98530327480470	-1.11303833164508	1.36846323033625
H	-4.01976472034328	-2.15231420534271	1.06100359819693
H	-4.87508997415088	-0.57567556041594	1.06312492412630
H	-3.89162837526913	-1.06648932377910	2.45338067249048
54			
D1-A2d			

C	9.95086166616435	-4.84675047420054	-1.28447957198936
C	10.07205083197661	-3.48858214825804	-0.98732866949425
C	8.69484151114830	-5.44277872392222	-1.33454058811996
C	7.53723123871710	-4.70896975159548	-1.08466510664663
C	8.93289504932860	-2.74909394762218	-0.73881219631650
C	7.67725454918671	-3.36171902175465	-0.77842961151714
N	6.68444486217707	-2.42713544748846	-0.45398246713983
C	8.76739640895848	-1.28521827177449	-0.42601592690311
C	7.32241530749249	-1.20992623189745	0.10624066374926
H	8.89264923536366	-0.68181799112050	-1.32903976697740
H	9.47863174448760	-0.92014015907794	0.31517936899977
C	7.27106392348214	-1.27376587814991	1.62949708098406
H	6.79726630032124	-0.33317741745449	-0.26859318255528
H	6.25282584477537	-1.42598687243083	1.98612723588510
H	7.65733105193020	-0.34917212013059	2.06056890833868
H	7.88611190174276	-2.10254775155702	1.98552728505622
C	5.35206809909288	-2.61941243510526	-0.49141567461479
C	4.41392204447339	-1.67296104703267	-0.17935719993664
C	3.02965356300195	-1.89744239305878	-0.23496060647841
C	2.11248107506847	-0.87518213208118	-0.02644197430781
C	0.74104293217349	-1.17456444108810	-0.14272559223518
C	-0.41210623249026	-0.39487187842607	-0.12423262506856
H	8.61077717799369	-6.49577262395815	-1.56932584054562
H	6.56924225759549	-5.18953691975875	-1.11616568592003
H	5.03885224113262	-3.60250239142166	-0.82054326148535
H	4.72353366298659	-0.67875520878815	0.10908571509984
H	2.64649331951242	-2.88497981688495	-0.45751086101030
O	2.60801219778960	0.33644999046258	0.28666172604731
H	0.52970957537540	-2.23123362293839	-0.25247995053517
H	10.83628411227921	-5.43705745045109	-1.47781359791658
H	11.04725928769489	-3.01881712328151	-0.95134456147873
C	-1.67127128616626	-1.13233735757633	-0.10055098260322
C	-0.43945254448978	1.04237024664785	-0.19043928941995
O	0.57062687566060	1.75884877198769	-0.25107778547727
O	-1.73892113883123	-2.34970778555933	-0.03460489251098
H	1.88970004259543	1.01277309201221	0.13044260109723
N	-1.68446408694942	1.66754987301799	-0.22686298873213
C	-2.89939450559997	1.00184336978592	-0.21840058190761
N	-2.85610458446151	-0.37057743138285	-0.14598923551956
S	-4.34063078985543	1.83566175246982	-0.29916228644197
C	-1.66057705145728	3.14181155738316	-0.34039319567485
H	-2.54968775626092	3.51012767503196	0.15908837501042
C	-1.62003198180384	3.60309309453053	-1.79110821077000
H	-0.77667587542917	3.47609276622181	0.19263377478946
C	-4.11457217080794	-1.14365321231281	-0.08700248828712
C	-4.61048934475934	-1.32633129994980	1.33994439935210
H	-3.89698721848829	-2.10584151920745	-0.53946018335231
H	-4.84506331892102	-0.61008195652572	-0.68606405178624
H	-3.86945210237092	-1.85540522483132	1.93957139585680
H	-5.52799801438605	-1.91776349749686	1.33751603145625
H	-4.82992414201920	-0.36328531809496	1.80062102789600

H	-0.72349871051270	3.23382509509134	-2.28917026406463
H	-1.60835204869699	4.69405903256670	-1.83273650985678
H	-2.50055498692192	3.25249997843748	-2.32935812402124
55			
D2-A2d			
C	9.76548341814975	-5.25069376879235	-0.67879006914499
C	10.01078060786923	-3.89331490050691	-0.45076896106704
C	8.44956658415714	-5.71783305584696	-0.76899387289397
C	7.37275557691830	-4.85604407317557	-0.63592073014116
C	8.93176694946554	-3.03423135692925	-0.31951374497743
C	7.62832398206644	-3.50674350864550	-0.40936872354274
N	6.71281509183066	-2.45346956939998	-0.24996191482050
C	8.92441248344319	-1.54907700287775	-0.06135846134303
C	7.42770320868672	-1.17276632805866	-0.12020487873555
H	9.50366089660104	-1.00115142537819	-0.80561647973081
H	9.35305527046179	-1.31720847428852	0.91586931755781
C	5.37427245160168	-2.54658237301518	-0.26822447880787
C	4.50035012632599	-1.49412501424798	-0.17424130498313
C	3.11092947730400	-1.68449349583455	-0.20023278446066
C	2.18456513975075	-0.65153707921065	-0.12728424085271
C	0.82486570295286	-1.00880006348170	-0.17734720022386
C	-0.37144720691169	-0.29850358349734	-0.14979903189543
H	8.29167642786077	-6.77243848342077	-0.94908585316338
H	6.36559278281336	-5.24122894698734	-0.71410041905183
H	4.98709348670218	-3.55322503078802	-0.36722259414960
H	4.86544038236761	-0.48033974184189	-0.08492708576886
H	2.72153416237405	-2.69083123853657	-0.28589282637031
O	2.65502709343942	0.60316399564438	-0.01701903093833
H	0.66257548324955	-2.07613743804494	-0.25489117383665
O	10.73757830199314	-6.19033400564893	-0.82862373223938
H	11.01769411197976	-3.50877172545693	-0.37856463458201
C	-1.57578862338559	-1.11802489888204	-0.22941893559172
C	-0.49825016323694	1.13086177569051	-0.05534707740973
O	0.45579347520995	1.92031660767598	-0.00393759416485
O	-1.56445527105046	-2.33845200902315	-0.27751615495233
H	1.87865895525638	1.22957932454890	-0.00139047380566
N	-1.78718413970910	1.66396700845144	-0.00755056984253
C	-2.94990725102660	0.92404046263510	-0.13915726429883
N	-2.80661029228731	-0.43629855451176	-0.26808977645837
S	-4.44894324717704	1.65447893266526	-0.14108881780659
C	-1.87155974895150	3.13181139554837	0.14679130489607
H	-2.78536852481120	3.33795278437968	0.69365204703353
C	-1.86698880315193	3.85288422804803	-1.19272388051996
H	-1.01311581718524	3.42642539852408	0.74214739526063
C	-4.00055779466313	-1.29391265701117	-0.40182685621038
C	-4.54388169476365	-1.72967575167905	0.95060025866773
H	-3.68804147819111	-2.15699788729638	-0.98155376741394
H	-4.74182317191721	-0.72578356855670	-0.95428541665826
H	-3.79447256666902	-2.30095081007917	1.49888657962282
H	-5.41975171407673	-2.36513786742564	0.80649081370198
H	-4.84418220799238	-0.86478840917398	1.54198024381153

H	-0.94606215772025	3.64769668415246	-1.73888616591925
H	-1.93409876122158	4.92991156421083	-1.02750360856770
H	-2.72113118018766	3.54758609208763	-1.79686139578519
C	12.09110270632258	-5.78193292461715	-0.72466173465600
H	12.68687757207768	-6.68153939421541	-0.85740692701692
H	12.30197486187951	-5.34952279055443	0.25813821424287
H	12.35005957557932	-5.05728405937134	-1.50267821141595
H	7.19285179022113	-0.54198807954074	-0.98065544452830
H	7.08678367937526	-0.65404490841183	0.77640812594878
59			
D3-A2d			
C	9.85933817851793	-5.14175968309457	-0.14729507550931
C	10.05317852179321	-3.77191937708754	-0.43404627380416
C	8.53646593015819	-5.58807780037638	0.05681843025343
C	7.45327745780573	-4.72210638694628	0.00558830604550
C	8.97260017064889	-2.91635661330644	-0.48230219833306
C	7.67957249058895	-3.38059283801631	-0.26449536414765
N	6.75222246333131	-2.33111046222371	-0.37657823872552
C	8.94275015134123	-1.43667625188085	-0.77375913780764
C	7.44935416837133	-1.06158048745726	-0.64310857887448
H	9.31864473863575	-1.22266797304636	-1.77602851491465
H	9.55777196490397	-0.87216842600871	-0.07152068274904
C	5.42193757115268	-2.42775892720870	-0.25821128028106
C	4.52766800914267	-1.39043616766125	-0.35761403338584
C	3.14830386536657	-1.60260332912869	-0.23882448755900
C	2.19125279129684	-0.59912514625640	-0.34690510182413
C	0.84567132853234	-0.98836776838246	-0.24018141341681
C	-0.37376921351705	-0.31949686891499	-0.32943168260423
H	8.34262534852467	-6.62909322115864	0.26130229544345
H	6.45787753305430	-5.11079791203326	0.17192852612616
H	5.05267163516692	-3.42897219532230	-0.07278122272477
H	4.86936084692879	-0.38048984497416	-0.53775561929236
H	2.78815706821346	-2.60799328915642	-0.06155690880300
O	2.62588461491128	0.65557574523374	-0.56079571761804
H	0.71603091738504	-2.04796363086787	-0.06190269065691
N	10.92908893628934	-6.01358626207586	-0.05922483285362
H	11.04298870641833	-3.38159983264662	-0.61701535063766
C	-1.55143472172211	-1.16417522025305	-0.17496069141742
C	-0.54204470269517	1.08914047436457	-0.55617739069542
O	0.38894905228264	1.89968935254801	-0.67527188748330
O	-1.50328317722203	-2.36592065054684	0.04161499014037
H	1.83106919932236	1.25602608839744	-0.61978193428157
N	-1.84672799451263	1.58021678497795	-0.64190442185068
C	-2.98740453226588	0.81517377279729	-0.47235233132945
N	-2.80560130629872	-0.53306599021356	-0.28286937284404
S	-4.50718492217123	1.50355841385166	-0.49099261449216
C	-1.97437563218417	3.03385681386135	-0.87903890469326
H	-1.12575560612976	3.31916520296491	-1.49269703577493
C	-1.98851020578123	3.82822118721926	0.41840877440509
H	-2.89429536090844	3.18284656176264	-1.43389221127630
C	-3.97496029655351	-1.41889072097797	-0.12012555787140

C	-4.39319073433897	-1.55236205836515	1.33645833161884
H	-3.67906917736141	-2.38420135662183	-0.51960494303024
H	-4.77764546234827	-1.00135220701907	-0.71881264416371
H	-3.58437853366148	-1.97858659088976	1.93041166951189
H	-5.25716780328194	-2.21565482993736	1.41060469008834
H	-4.67239529384298	-0.58228821279751	1.74773234776882
H	-2.82869158080467	3.52787619998915	1.04417500982133
H	-2.09384952235801	4.89163299955981	0.19482068974344
H	-1.05844241979848	3.68525309418181	0.96897282481943
C	12.23554973813797	-5.58001159415570	-0.51004804476568
C	10.67827572529561	-7.43942210080027	0.00344078982879
H	12.25346976739307	-5.32761998412865	-1.57927637821384
H	12.95357290125130	-6.37760994250406	-0.33518379229182
H	12.57370114106113	-4.70592942852047	0.05040868077154
H	11.62756889960061	-7.96240402938881	0.09042025687350
H	10.15625593443178	-7.81719401450323	-0.88619234779644
H	10.08039034139940	-7.69409240214146	0.88102712201284
H	7.04738949830233	-0.61162529815236	-1.55207001148613
H	7.26469059279941	-0.36956536456007	0.18113318700801

References

1. Neese, F., Software update: the ORCA program system, version 4.0. *Wiley Interdisciplinary Reviews: Computational Molecular Science* **2018,** *8* (1), e1327.
2. Perdew, J. P.; Burke, K.; Ernzerhof, M., Generalized gradient approximation made simple. *Physical Review Letters* **1996,** *77* (18), 3865-3868.
3. Adamo, C.; Barone, V., Toward reliable density functional methods without adjustable parameters: The PBE0 model. *Journal of Chemical Physics* **1999,** *110* (13), 6158-6170.
4. Weigend, F.; Ahlrichs, R., Balanced basis sets of split valence, triple zeta valence and quadruple zeta valence quality for H to Rn: Design and assessment of accuracy. *Phys Chem Chem Phys* **2005,** *7* (18), 3297-305.
5. Grimme, S.; Antony, J.; Ehrlich, S.; Krieg, H., A consistent and accurate ab initio parametrization of density functional dispersion correction (DFT-D) for the 94 elements H-Pu. *J Chem Phys* **2010,** *132* (15), 154104.
6. Becke, A. D.; Johnson, E. R., A density-functional model of the dispersion interaction. *J Chem Phys* **2005,** *123* (15), 154101.
7. Dutta, A. K.; Nooijen, M.; Neese, F.; Izsak, R., Automatic active space selection for the similarity transformed equations of motion coupled cluster method. *J Chem Phys* **2017,** *146* (7), 074103.
8. Dutta, A. K.; Nooijen, M.; Neese, F.; Izsak, R., Exploring the Accuracy of a Low Scaling Similarity Transformed Equation of Motion Method for Vertical Excitation Energies. *J Chem Theory Comput* **2018,** *14* (1), 72-91.
9. Nooijen, M.; Bartlett, R. J., Similarity transformed equation-of-motion coupled-cluster theory: Details, examples, and comparisons. *J Chem Phys* **1997,** *107* (17), 6812-6830.
10. Nooijen, M.; Bartlett, R. J., A new method for excited states: Similarity transformed equation-of-motion coupled-cluster theory. *J Chem Phys* **1997,** *106* (15), 6441-6448.
11. Sous, J.; Goel, P.; Nooijen, M., Similarity transformed equation of motion coupled cluster theory revisited: a benchmark study of valence excited states. *Molecular Physics* **2013,** *112* (5-6), 616-638.

12. Dutta, A. K.; Neese, F.; Izsak, R., Towards a pair natural orbital coupled cluster method for excited states. *J Chem Phys* **2016,** *145* (3), 034102.
13. Weigend, F., Accurate Coulomb-fitting basis sets for H to Rn. *Phys Chem Chem Phys* **2006,** *8* (9), 1057-65.
14. Liakos, D. G.; Sparta, M.; Kesharwani, M. K.; Martin, J. M.; Neese, F., Exploring the Accuracy Limits of Local Pair Natural Orbital Coupled-Cluster Theory. *J Chem Theory Comput* **2015,** *11* (4), 1525-39.
15. Barone, V.; Cossi, M., Quantum Calculation of Molecular Energies and Energy Gradients in Solution by a Conductor Solvent Model. *The Journal of Physical Chemistry A* **1998,** *102* (11), 1995-2001.
16. Avila Ferrer, F. J.; Santoro, F., Comparison of vertical and adiabatic harmonic approaches for the calculation of the vibrational structure of electronic spectra. *Phys Chem Chem Phys* **2012,** *14* (39), 13549-63.
17. Berraud-Pache, R.; Neese, F.; Bistoni, G.; Izsak, R., Computational Design of Near-Infrared Fluorescent Organic Dyes Using an Accurate New Wave Function Approach. *J Phys Chem Lett* **2019**, 4822-4828.
18. Bannwarth, C.; Ehlert, S.; Grimme, S., GFN2-xTB-An Accurate and Broadly Parametrized Self-Consistent Tight-Binding Quantum Chemical Method with Multipole Electrostatics and Density-Dependent Dispersion Contributions. *J Chem Theory Comput* **2019,** *15* (3), 1652-1671.
19. Laurent, A. D.; Medved, M.; Jacquemin, D., Using Time-Dependent Density Functional Theory to Probe the Nature of Donor-Acceptor Stenhouse Adduct Photochromes. *Chemphyschem* **2016,** *17* (12), 1846-51.
20. Garcia-Iriepa, C.; Marazzi, M., Level of Theory and Solvent Effects on DASA Absorption Properties Prediction: Comparing TD-DFT, CASPT2 and NEVPT2. *Materials (Basel)* **2017,** *10* (9).
21. Hemmer, J. R.; Page, Z. A.; Clark, K. D.; Stricker, F.; Dolinski, N. D.; Hawker, C. J.; Read de Alaniz, J., Controlling Dark Equilibria and Enhancing Donor-Acceptor Stenhouse Adduct Photoswitching Properties through Carbon Acid Design. *J Am Chem Soc* **2018,** *140* (33), 10425-10429.
22. Hemmer, J. R.; Poelma, S. O.; Treat, N.; Page, Z. A.; Dolinski, N. D.; Diaz, Y. J.; Tomlinson, W.; Clark, K. D.; Hooper, J. P.; Hawker, C.; Read de Alaniz, J., Tunable Visible and Near Infrared Photoswitches. *J Am Chem Soc* **2016,** *138* (42), 13960-13966.
23. García-Iriepa, C.; Marazzi, M.; Sampedro, D., From Light Absorption to Cyclization: Structure and Solvent Effects in Donor-Acceptor Stenhouse Adducts. *ChemPhotoChem* **2019,** *3* (9), 866-873.
24. Lerch, M. M.; Di Donato, M.; Laurent, A. D.; Medved, M.; Iagatti, A.; Bussotti, L.; Lapini, A.; Buma, W. J.; Foggi, P.; Szymanski, W.; Feringa, B. L., Solvent Effects on the Actinic Step of Donor-Acceptor Stenhouse Adduct Photoswitching. *Angew Chem Int Ed Engl* **2018,** *57* (27), 8063-8068.
